# Study of mouse behavior in different gravity environments

**DOI:** 10.1038/s41598-021-82013-w

**Published:** 2021-01-29

**Authors:** Michihiko Shimomura, Akane Yumoto, Naoko Ota-Murakami, Takashi Kudo, Masaki Shirakawa, Satoru Takahashi, Hironobu Morita, Dai Shiba

**Affiliations:** 1 Mouse Epigenetics Project, ISS/Kibo Experiment, Japan Aerospace Exploration Agency, Tsukuba, Japan; 2 JEM Utilization Center, Human Spaceflight Technology Directorate, Japan Aerospace Exploration Agency, Tsukuba, Ibaraki Japan; 3 Tsukuba Division, Advanced Engineering Services Co. Ltd., Tsukuba, Japan; 4 Laboratory Animal Resource Center in Transborder Medical Research Center, and Department of Anatomy and Embryology, Faculty of Medicine, University of Tsukuba, Tsukuba, Japan; 5 Space Biology Laboratory in Transborder Medical Research Center, Faculty of Medicine, University of Tsukuba, Tsukuba, Japan; 6 Department of Management Nutrition, Tokai Gakuin University, Kakamigahara, Japan; 7 Department of Physiology, Gifu University Graduate School of Medicine, Gifu, Japan

**Keywords:** Neuroscience, Sensorimotor processing

## Abstract

Many experiments have analyzed the effect of the space environment on various organisms. However, except for the group-rearing of mice in space, there has been little information on the behavior of organisms in response to gravity changes. In this study, we developed a simple Active Inactive Separation (AIS) method to extract activity and inactivity in videos obtained from the habitat cage unit of a space experiment. This method yields an activity ratio as a ratio of ‘activity’ within the whole. Adaptation to different gravitational conditions from 1*g* to hypergravity (HG) and from microgravity (MG) to artificial 1*g* (AG) was analyzed based on the amount of activity to calculate the activity ratio and the active interval. The result for the activity ratios for the ground control experiment using AIS were close to previous studies, so the effectiveness of this method was indicated. In the case of changes in gravity from 1*g* to HG, the ratio was low at the start of centrifugation, recovered sharply in the first week, and entered a stable period in another week. The trend in the AG and HG was the same; adapting to different gravity environments takes time.

## Introduction

Living creatures, including humans, have evolved under the influence of terrestrial gravity (1*g*). The effects of the space environment on human physiology include degradation of skeletal weight-bearing bone mass and muscle, as manifested among astronauts who stayed in outer space for an extended period. Such changes resemble age-related diseases on the ground, and have been found to progress rapidly in space^1,2^. Like humans, mice are vertebrates and have a similar genome structure. Mice are fast-growing model organisms suitable for studying the effects of living in the microgravity of space^3^. To date, space experiments on rats and mice, have been performed in the International Standard Payload Racks (ISPR) more than 70 times, including five experiments using the mouse habitat unit (MHU) of the Japan Aerospace Exploration Agency (JAXA)^3,4^. Although, using mice and other organisms, analysis of tissues and genes in a microgravity environment has mainly been performed to elucidate the mechanisms of adaptation to the space environment [http://www.spacestationresearch.com/wp-content/uploads/GeneLabStrategicPlan_Baseline_2014.pdf]^5,6^, activity analyses of mice in a gravity environment different from that on the ground is also important for estimating the effects of gravity on humans. However, there are many restrictions on in-flight experiments, such as upmass (payload weight launched into the International Space Station (ISS)), crew time (working time of the crew in ISS), and experimental space (rearing mice in cages), so activity analysis is usually conducted in a home-cage activity test. Regarding behavioral analysis, group-rearing behavior in space is the only research reported so far^7^. Identification of individuals is difficult in group-rearing, but individual-rearing makes it possible to observe behavior using videos over a long period.

In this study, we developed the Active Inactive Separation (AIS) method to analyze mouse behavioral patterns to extract activity and inactivity in videos under microgravity (MG) and artificial 1*g* (AG) environments. This method yields an activity ratio as a ratio of ‘activity’ in the whole because it separates ‘activity’ and ‘inactivity’. The mice were individually reared in a habitant cage unit (HCU) in space (developed by our team) and a camera attached to the HCU was used to obtain visual data on the rearing status of each mouse. The amount of activity (behavior) ratio and time of active interval were measured using the moving images obtained from the rearing cages in space. This is the first report of individual-rearing behavioral analysis in space. Furthermore, the moving images obtained from the space experiment were compared to those from the ground control. Additionally, to examine the impact of gravitational gradient that occurred when AG was generated on board by centrifugation at 77 rpm, a 1.4*g* centrifugation experiment was executed on the ground and videos were also analyzed using this method.

## Results and discussion

The experiments were executed under conditions of hypergravity (HG) on the ground, flight (MG, and AG) (FL), and ground control (GC). The 1.4*g* HG experiment was conducted to evaluate the effect of inducing the formation of a gravitational gradient inside the rearing cage at simulated JAXA’s Centrifuge-equipped Biological Experimental Facility (CBEF) with a short radius (0.15 m) and 77 rpm centrifugation. Since this may have some effects on the mice, the biological responses to HG induced by a short-arm centrifuge were examined.

**Figure 1:**
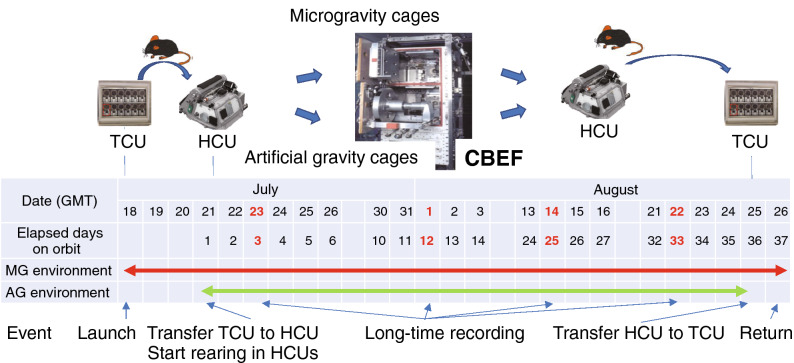
FL experimental event date. Twelve male mice were launched to ISS on July 18, 2016. Then the mice were transferred from TCU to HCU on July 21, 2016, divided into two groups (six mice in MG and six mice in AG) and installed into CBEF. Before reaching the ISS, the mice were exposed in MG environment for about 3.5 days. During on board period, videos were taken on the dates indicated in red. The mice were returned to the earth on August 26, 2016.

The behavior of the mice in the GC, HG, AG, and MG experiments was recorded in video format (Supplementary Tables 1–3). The mice were reared for 7 days in the centrifuge equipment cages before centrifugation. The HG experiment was conducted from July 29 to August 27, 2014, at Gifu University using four male C57BL/6J mice in 1.4*g* environment. After launching on July 18, 2016, the mice stayed in the microgravity environment for 3.5 days in TCU cages. Next, the FL experiment under the MG and AG conditions was conducted at the Japanese Experimental Module (Kibo) onboard the ISS from July 21 to August 26, 2016 (the mice were reared for 34.6 days under MG (n = 6) environment and 34.1 days under AG (n = 6)) (Fig. 1). Furthermore, the recorded AG (A1 cage) and MG (M1) videos on the 3rd and 33rd days were indicated as Supplementary AG and MG videos. The mice were reared for 4.5 days in TCU cages from September 22. The GC experiment was conducted from September 26 to November 3, 2016, at the Tsukuba Space Center of JAXA under the same conditions (temperature, humidity, and light–dark cycle) as the FL experiment with six mice.

The activities of the mice were analyzed through the percentage of active and inactive periods (active ratio) from the AIS method by comparisons with previous studies of video recordings on wake and sleep patterns using EEG/EMG. In particular, the results calculated by the AIS method correspond to the behavior and movement of the mice under different experimental conditions.

### Activity ratio based on times of day

**Figure 2:**
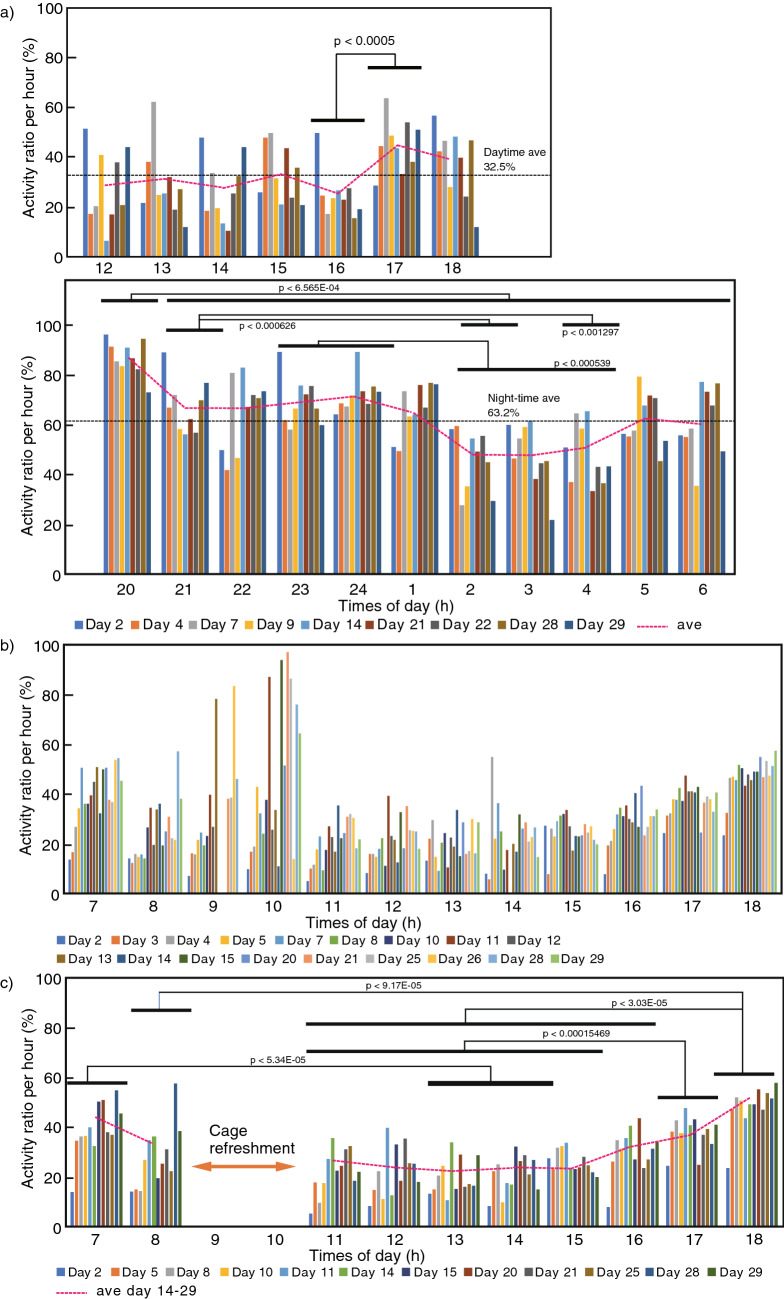
Time-based on the activity ratio per 1-h average. Activity ratio per 1-h average in GC (**a**), HG (**b**), and HG with 1 h removed after refreshment (**c**). The horizontal axis is elapsed time (h), and the vertical axis is activity ratio (%). The dashed line represents the average activity ratio. The horizontal bar in the figure shows the range of p-values. Average 32.5% and 63.2% in the figure are the average activity ratio during daytime and night-time. The recording time varies, so (**a**) is displayed from 12:00 to 07:00.

The behavior and movement of the mice in the GC, HG, MG, and AG conditions were extracted by video tracking. The hourly average activity ratio, based on the percentage of pixel change in the entire arena between the current sample and the previous sample, was analyzed after removing the flicker noise (Fig. 2, Supplementary Fig. 1).

In the case of GC, dashed line activity ratio tended to increase just before entering night-time; actually activity ratio at 17:00 is significantly increased compared with at 12:00–16:00 (Fig. 2a, Supplementary Table 4). Immediately after the switch from daytime, the activity ratio increased, followed by a decrease from 02:00 to 04:00. Before switching to daytime, activity ratio increased again; however, the increase was smaller than after switching from daytime. The pattern of the GC activity ratio became constant after 12:00 in daytime and was followed by a gradual increase near night-time. The activity ratio peaked immediately after the onset of night-time, followed by a decrease and a gradual increase until the onset of daytime. These activity patterns are consistent with previous data assessing mouse behavior under a light/dark cycle^8^. The average activity ratio at daytime and night-time is 32.5% and 63.2%, respectively, with the night-time ratio twice that of the daytime average activity ratio.

The mouse activity ratio increased in the daytime and before night-time (videos could not be used due to a problem with the infrared light settings) under HG conditions. There was an increase in activity ratio from 09:00 to 10:00, which coincided with the cage refreshment (Fig. 2b). A similar phenomenon was also reported in mice after cage-cleaning or cage change^9,10^. Upon removal of the activity ratio in this 2 h time-frame, the activity ratio became stable after 7 days (Fig. 2c, Supplementary Table 5). The HG activity ratio pattern showed an increase shortly after the onset of daytime at 07:00, then became constant at 13:00–15:00, followed by an increase from 16:00 to 18:00. A similar activity ratio pattern was also reported in mice subjected to a light/dark cycle in a home cage^8^.

### Activity ratio based on elapsed days

**Figure 3:**
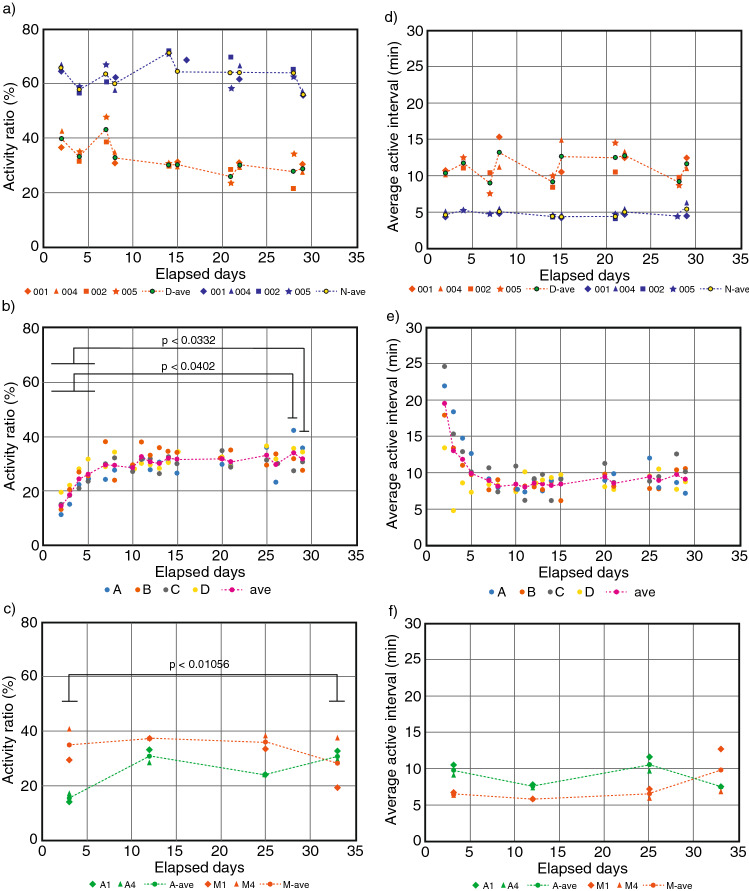
The activity ratio and active interval for elapsed days. The activity ratio and active interval for elapsed days in GC (**a**, **d**) blue dots at night-time and orange dots at daytime, HG (**b**, **e**) at daytime, green dots at AG and red dots at MG (**c**, **f**). The horizontal axis is elapsed days, and the vertical axis is the activity ratio (%). The dashed line represents the average activity ratio. The horizontal bar in the figure shows the range of p-values.

The activity ratio in GC, HG, MG, and AG conditions for situations where the mouse was mobile and active, and situations where it was immobile and inactive (Fig. 3a–c) showed the adaptation of mouse to different gravity environments. The results of statistical analysis are shown in Supplementary Tables 6–8.

**Figure 4:**
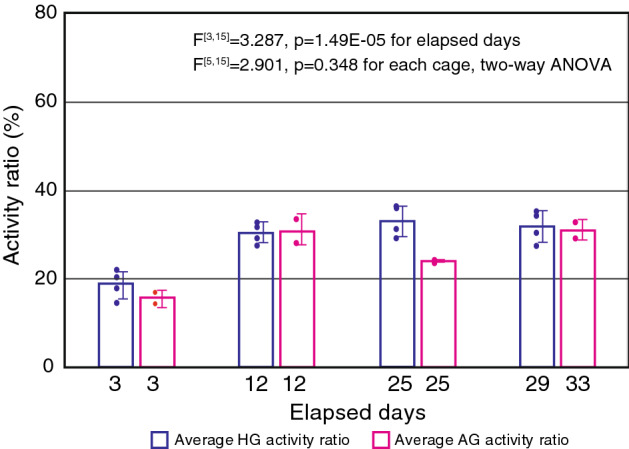
Comparison of HG and AG activity ratio. The horizontal axis is elapsed days, and the vertical axis is HG and AG activity ratio (%). Boxes are average activity ratios, and dots are activity ratios. The last day of AG is 33 days, and the last day of HG is 29 days, so they were considered the same day.

The activity ratio at GC in daytime was 40% on the second day and was around 30% after day 14. The activity ratio in GC night-time was 65% on the second day and fluctuated around 65% after that, although there was some increase and decrease. The activity ratio in HG decreased by half on the second day after the start of centrifugation but became stable at the range of 30–32% after day 7. Thus, activity ratio in HG recovered after 1 week, by which time the mice had become accustomed to the new environment. This means that if the gravity environment changes, the mice can adapt to the new environment after one week. The activity ratio on elapsed days 2–5 was significantly lower than that on days 28 and 29 (Fig. 3b). This can be thought of as a recovery time in terms of behavior, especially the vestibular related behavior control. In this regard, Hallgren et al.^11^ investigated the effect of 6 months of microgravity on the vestibular-mediated ocular response in a large group of astronauts, and found that ocular counter-rolling response was decreased at 2–5 days after return, but recovered to preflight levels at 9 days after return, indicating that the peripheral otolith system can achieve full recovery within 9 days. Although, it is unknown whether the vestibular-related behavior was also recovered within 9 days, the activity recovery curve in Fig. 3b (HG) and c (AG) might reflect to a recovery of the vestibular function. The activity ratio in MG reached about 35% from day 3 (as of the third day, mice stayed in microgravity for 6.5 days) to day 25 after relocation to the HCU cage and reached 30% on day 33. The activity ratio in AG was reduced to half on the third day after the start of centrifugation, but recovered on day 12. The activity ratio recovered pattern under AG was similar to that under HG (Fig. 4).

It was difficult to compare the daytime activity ratio in MG and GC because the measurement timing was different. However, the activity ratio was slightly higher in MG than the stable period of GC (daytime) and HG because this was under the microgravity environment, and it may have been easier for the mice to move. In the latter half, it is assumed that the activity ratio became stable as the mice became acclimatized to the environment (Supplementary Tables 6 and 8).

### Comparison of the activity ratio in the study and previous studies

The activity ratio calculated by the AIS method was compared with the results of the previous studies using EEG/EMG, and videos on the ground. The amount of mouse activity ratio is often indicated by wake and sleep time and the distance of locomotion or wheel, and varies with strain, sex, and age^12–14^. Previous studies on measurements of awake/active, and rest/immobility/inactive measured using video and EEG/EMG are summarized in Supplementary Table 9. The estimated average activity ratio is 51.2% in the 6-week old C57BL/6 strain^15^ (Supplementary Table 9, No. 1) and 34% in the 11–14-week old C57BL/6J strain^16^ (Supplementary Table 9, No. 2). In this method, if there are no center of gravity shift in units of 1 cm, detection is difficult, and the inactive state in WT of Fig. 3a may be increased^16^. The average of the activity ratio estimates is 68.5% with C57BL/6J^12^ (Supplementary Table 9, No. 3), and 45% with C57BL/6^17^ (Supplementary Table 9, No. 4). In this value, since daytime and night-time are combined, we think that the range is 23–70%. Based on Supplementary Table 9, Nos. 5, and 6, the average of the activity ratio is estimated at 43–45%^18,19^. Based on Supplementary Table 9, Nos. 7, and 8, the average of the activity ratio estimates is 51–53%^20,21^.

In video measurement, the average all-day activity ratio was in a range of 45–68%, depending on the measurement method^12,15,16^. In EEG/EMG measurement, the average all-day activity ratio was in a range of 44–53%, narrower than the video measurement^17–21^. The comparison of the data acquired by videos and data acquired by EEG/EMG showed that if the data is continuous for 40 s or more, the degree of coincidence between video and EEG/EMG is 95%^17^. In video measurement, fine movement may also tend to appear large (Supplementary Table 9, No. 4).

The result of the activity ratio obtained from the measurement method fitted to the MHU was 47.6% on all-day average in GC (Supplementary Table 9, No. 9). This result is close to the values shown in Supplementary Table 9, Nos. 4, 5, and 6. In corpore, the mouse does not move even when in the waking state, so it is considered that values such as Supplementary Table 9, Nos. 7, and 8 are acceptable. From these results, we are convinced that our method is enough reliable.

### Active interval

The active interval is the average duration of the active time between active states; activity with moving for more than 30 s was classed as active and non-moving for more than 30 s was classed as inactive. The active intervals of GC, HG, and FL (MG, AG) are shown in Fig. 3d–f. Results of statistical analysis are shown in Supplementary Tables 10–12. The average active interval under GC was in the range of about 8–12 min at daytime and about 4–5 min at night-time. The average active interval under HG decreased from a maximum of 20 min on day 2 to 8 min on day 5 (comparison day 28, day 29, and day 2, p < 0.0106, and comparison day 28, day 29, and days 3–26, p > 0.0698, Fig. 3e, Supplementary Table 11). After that, the average gradually converged towards 10 min. The average active interval under AG changed at about 8–10 min intervals from the start until the end of centrifugation. The average active interval under MG was about 6–7 min at the start and about 10 min at the end of centrifugation.

For daytime, the active intervals converged to an average of 10 min. A centrifugal force with the same rotation speed was applied, but the active intervals in AG and HG at the early stage of centrifugation differed around 10 min for AG and around 20 min for HG. Since MG has a value close to 6 min and a value close to the night-time of GC, it appears that it may be easier to move in the microgravity than in the gravity environment. In addition, the active interval of HG and MG eventually approached 10 min, so it may actually take this long to adapt to the environment. Due to the restriction of video storage hard disk size on the orbit and video channel, recorded videos were two AG and two MG videos. However, since the space experiment was conducted for a limited time and opportunity, the results obtained are valuable as basic data. It would be necessary to extend the period of exposure to the gravity environment and increase the number of samples in future studies.

### Mouse behavior and vestibular function

Environmental changes can contribute to mouse behavioral changes such as alertness and anxiety. Anxiety-like behavior can be examined using the elevated plus maze (EPM) and so on^22,23^. Because mice are stressed by high plasma corticosterone levels associated with anxiety-related behaviors during EPM^24^.

On the other hand, the cause of the decrease in activity immediately after the change in gravity is thought to be plastic changes in the vestibular system and/or stress-induced freezes. As a specific example, the following effects from vestibular organs were confirmed. Mice with sham operation (the tympanic membrane was removed, but the auditory ossicles were left intact) and with VL operation (Vestibular lesion: removal of the tympanic membrane, malleus, incus, and stapes, labyrinthine fluid was aspirated) were examined for 3 days, 2 weeks, and 8 weeks under a 2 *g* centrifugation environment for gene expression of hypothalamic feeding neuropeptides. Among these genes, anorexia-inducing neuropeptide [CRH (corticotropin-releasing hormone) in PVN (the paraventricular hypothalamic nucleus), proopiomelanocortin in the neuropeptide Y] was expressed for 2 weeks or more, and the expression change disappeared in 8 weeks^25^. In rats, after 90 min of exposure to 2*g*, the immunoreactivity of CRH in PVN was significantly increased, but in VL it was weakened^26^ and ACTH (adrenocorticotropic hormone) and corticosterone concentrations in plasma increased significantly against 1*g* control^26^. From these, CRH-producing neurons in PVN may receive neuronal inputs from the vestibular system^27^. In addition, the centrifugal environment was stressful for mice because Fos was observed in PVN^26^. Moreover, it was found that rearing VL mice in a 2 *g* centrifugation environment for 2 weeks may contribute to the adaptive response of bone tissue in a gravity change partially through the vestibular system^28^. It can be presumed that the vestibular system adapts to the new environment, making it less stressful or the behavioral patterns through the vestibular system adapt to the new environment.

A study on the behavior of MG mice aboard the ISS for around 3 days showed a high activity ratio in the case of group rearing in a microgravity environment^7^. The vestibular system controls a variety of physical functions such as physical stability, sympathetic nerve activity, arterial pressure, feeding behavior, body temperature, and muscle and bone metabolism^27^. However, it is very plastic, and its function changes when exposed to various gravitational environments^27^. It can be presumed that mouse behavior in the microgravity environment is also mediated by the vestibular system.

Astronauts going into space often suffer from Space Motion Sickness (SMS), which can be triggered during the first hour or two of weightlessness and may continue for up to 72 h^29,30^. In addition, many astronauts experience similar symptoms after returning to Earth (MG to 1*g*)^29,30^. Several mechanisms are under scrutiny, such as sensory conflict in the inner ear and in the visual system, but the exact molecular basis for this phenomenon is not fully understood.

The results of our experiment showed that several days are needed for mice to adapt to a different gravity such as to HG, from 1 to 1.4*g* (Fig. 3b). Since the animal cages do not support video recording at the early stage of gravity change from 1*g* to MG, therefore mouse activity could not be observed or recorded in this phase. Taking into consideration that the inner ear sensory structure is morphologically conserved in human and animals, the adaptation to MG for mice takes up to several days, the same as for astronauts (Fig. 3c). Developing a new video monitor system to observe animal behavior during launch and return phase would further our understanding of SMS using animal experiments for physiological and behavioral analysis.

## Conclusion

In this paper, we examined the mouse activity ratio and active intervals under different gravity environments. In the case of changes in the activity ratio from 1 to 1.4*g* (HG), the activity ratio was low at the start of centrifugation, recovered sharply in the first week, and then entered a stable period in another week. In the case of MG, the trend was different from that of HG; the ratio is higher, and the stable period persists from the beginning, although the start of video monitoring is 6.5 days of the entering MG, indicating that adaptation of mice to different gravity takes time.

These behaviors are controlled via the vestibular system. When exposed to various gravity environments, the vestibular system affects various body functions. In the age of exploring the Moon and Mars and other planets, it is important to acquire a set of behaviors and tissues, cells, and genes of mice in different gravity environments, including microgravity and partial gravity. With a space station in low orbit such as ISS, the rearing mice can be returned to the earth and analyzed, but with far-away satellites and planets, it is difficult to return the rearing mice to the earth. We believe that the day will soon come when we will conduct contact and non-contact space experiments using video observation, vital data, image sensing data, and RNA and DNA analysis information using minute samples in automatic rearing. Furthermore, in the more distant future, the day may come when the movement of genes, cells, and tissues can be predicted using video observation, vital data, image sensing data, and minute samples as clues.

## Methods

In this study, we developed a simple Active Inactive Separation (AIS) method to extract activity and inactivity in videos obtained from the habitat cage unit of a space experiment. The activity in different environmental conditions from hypergravity to microgravity was analyzed based on the amount of activity to calculate the activity ratio and the active interval.

### Animals

Eight weeks old male C57BL/6J mice (Stock #000664) were obtained from the Jackson Laboratory (USA) for the FL experiment and from the Charles River Laboratories (Japan) for the GC and HG experiments. On the day before the launch, all the mice were in good health conditions and showed similar physical characteristics with average mice body weights ranging from 22.4 to 22.9 g. The FL and GC experiments were approved by the Institutional Animal Care and Use Committee (IACUC) of University of Tsukuba (No. 16-048), JAXA (Protocol Number: 016-014B) and NASA (Protocol Number: NAS-15-004-Y1), and experiments were conducted according to the guidelines and applicable laws in Japan and the United States of America. The HG experiment was approved by the Animal Research Committees of Gifu University and the IACUC of JAXA (Protocol Number: 014-007), and experiment was conducted according to the “Guiding Principles for Care and Use of Animals in the Field of Physiological Science” set by the Physiological Society of Japan.

### Rearing equipment and camera

The HG experiment was performed using a specifically designed short-arm centrifuge. The radius of gyration at the center of the individual cage was adjusted to 0.15 m so that centrifugation at 77 rpm produced a centrifugal force of 1*g* and a resultant gravity of 1.41*g* on earth^31^. The individual cage used for rearing (0.01 m^2^ floor area, 0.0008 m^3^ volume) was installed on each gondola^31^. The camera attached to the centrifugal gondola was a 1/3 in. interlace CCD image sensor (NTSC) with effective pixels of 976 (H) × 494 (W). Video data from the camera was transmitted by wireless LAN to a PC after analog-to-digital conversion.

For the FL experiment, six HCU cages for MG and six HCU for cages for AG were installed with a rotation radius of 0.15 m on the CBEF. The individual rearing cage had a floor area of 0.0101 m^2^ and a volume of 0.00056 m^3^^32^. A 1/3-in. wide interlace CCD image sensor (NTSC) with 768 (H) × 494 (W) effective pixels was attached to each HCU. Video data from the camera were transmitted to JAXA at a transfer rate of 6 Mbps after analog-to-digital conversion^4^. For the GC experiment, six HCU ground model cages and camera with the same specifications were used. Video data from a camera was transmitted by LAN to PC after analog-to-digital conversion.

### Rearing condition and video information

The 12-h light and 12-h dark cycle^4,31^ was used in HG and FL experiments with daytime at 07:00–19:00 and night-time at 19:00–07:00, and GC experiment with daytime at 08:00–20:00 and night-time at 20:00–08:00. In the HG experiment, all the mice had access to food and water ad libitum, and the room temperature was maintained at 24 ± 1 °C^31^. The cage refreshment, such as cleaning and replenishment of water and food, was 0.5 h^31^, so video recording was ceased for 1 h due to the cage refreshment.

In the FL experiment, average temperatures during the launch, onboard, and return phases were 25.8 °C, 23.0 °C, and 26.1 °C, respectively. The average relative humidity levels during the launch, onboard, and return phases were 36.1%, 46.1%, and 41.7%, respectively. The average relative humidity during the launch, onboard and return phases was 36.1%, 46.1% and 41.7%, respectively. The mice were fed CRF-1 (Oriental Yeast Co., Ltd., Tokyo, Japan). The drinking water was filtered sterilized water containing iodine (0.2 mg/l)^4^. In the GC experiment, both the TCU and HCU were placed in an air-conditioned room at 24 ± 2 °C and 40–65% humidity. Food and water were the same as the FL experiment^4^.

The HG experiment was conducted for 29 days, whereas the FL and GC experiments were conducted for 34 days. Video recording was conducted throughout the HG, FL, and GC experiments (Supplementary Tables 1–3) with 720 × 480 (HG and FL) and 704 × 480 (GC) frame size and 29.97 (HG) and 30 (FL and GC) fps rate of trio-recorded experiment video.

### Activity analysis by EthoVision

Original movies were converted to AVI movies by the AviUtl with 60 Hz flicker noise mitigation plugin (http://auf.jpn.xxxxxxxx.jp/flicker60_0.2.2.zip). To analyze the activity threshold, the video image was converted to monochrome. Each pixel in the image had a grayscale value, ranging from 0 (black) to 255 (white). With gray scaling, the range of grayscale values should be considered as the subject is defined. The remaining grayscale values were considered to be background. The activity threshold gives the threshold for the difference in grayscale values between a sample and the previous sample (EthoVision XT Reference manual 2012)^33^. The activity threshold for AG, MG, and GC is 17; in the case of HG, it is 3.

All pixel coordinates in the arena, not only those of the detected subject, were determined immediately after they have been detected. The grayscale values of all pixels were compared with the previous sample to determine the number of changed pixels in the arena between the two. The formula for Activity was simply the number of changed pixels (CPn) for the current sample divided by the total number of pixels (Pn) in the arena: Activity = CPn/Pn × 100 (EthoVision XT Reference manual 2012)^33^.

### Noise removal from the activity continuous

EthoVision (version 9) extracted activity continuous data from the removed flicker noise movies with a sampling time of 0.033 s (30 frames/s). EthoVision performs the analysis in three steps. (1) In Setup, analysis conditions are set. (2) In Acquisition, associated data and video are loaded. (3) In Analysis, analysis and output are executed. Details are shown in the Supplementary EthoVision execution.

When a target approaches, the camera picks up breathing and heartbeat. Heart rate is 310–840 beats per minute and respiratory rate is 80–230 breaths per minute (Johns Hopkins University, Animal Care and Use Committee, http://web.jhu.edu/animalcare/procedures/mouse.html). To remove the activity continuous data derived from the EthoVision results, a low-pass filter with 0–0.2 Hz bandwidth is used (to remove the frequency).

### Active/inactive separation (AIS) method

The inactive contained in the activity continuous data is low, so it is difficult to separate inactive and active using the exact values (antilog). So, to expand the inactive level, we chose a log transfer and generated a histogram from the result of log conversion (− log_0.9_X). The histogram is plotted on the right if the value of activity continuous at a certain time is high and plotted on the left if it is low. We call this method “AIS” (Active Inactive Separation). An example is shown in Supplementary Fig. 2. The histogram (Supplementary Fig. 2c) was separated into a mountain on the left and a mountain on the right. When the left mountain and the right mountain were identified by log converted activity continuous data, it could be determined that the left mountain was a histogram containing data such as noise and rest, and the right mountain was a histogram in which the mouse was behaving. (Supplementary Activity amount when sampled from time-course data.) Therefore, we decided to use a value that does not include the local minimum between the left mountain and the right mountain as the threshold.

### AIS activity analysis

The flowchart after the low-pass filter is shown in Supplementary AIS activity analysis R^34^ script flow. In Supplementary AIS activity analysis R script, “finding local maxima and minima in R” script obtained from https://stackoverflow.com/questions/34205515/finding-local-maxima-and-minima-in-r No. 1 was used as subroutines. The activity ratio (amount of activity) from active bins and inactive bins of an output file from the R script was calculated as follows:

$$ {\text{Activity ratio}} = {{\text{active bins}} \mathord{\left/ {\vphantom {{\text{active bins}} {\left( {{\text{inactive bins}} + {\text{active bins}}} \right)}}} \right. \kern-\nulldelimiterspace} {\left( {{\text{inactive bins}} + {\text{active bins}}} \right)}} \times 100\left( \% \right) $$

‘Active’ refers to active time for more than 30 s, and ‘inactive’ refers to inactive time for more than 30 s, both calculated based on average active interval.

### Statistical processing

For statistical analysis, the Welch’s t test and multiple test by FDR (false discovery rate) were executed for Figs. 2 and 3. Statistical analysis of Fig. 4 was executed by the two-way ANOVA.

## Supplementary Information

Supplementary Information 1.

Supplementary Video 1.

Supplementary Video 2.

Supplementary Video 3.

Supplementary Video 4.

## Data Availability

All relevant data are within the paper and its “Supporting Information files”.
