# Author Correction: Study of mouse behavior in different gravity environments

**DOI:** 10.1038/s41598-021-96312-9

**Published:** 2021-08-27

**Authors:** Michihiko Shimomura, Akane Yumoto, Naoko Ota‑Murakami, Takashi Kudo, Masaki Shirakawa, Satoru Takahashi, Hironobu Morita, Dai Shiba

**Affiliations:** 1 Mouse Epigenetics Project, ISS/Kibo Experiment, Japan Aerospace Exploration Agency, Tsukuba, Japan; 2 JEM Utilization Center, Human Spaceflight Technology Directorate, Japan Aerospace Exploration Agency, Tsukuba, Ibaraki Japan; 3 Tsukuba Division, Advanced Engineering Services Co. Ltd., Tsukuba, Japan; 4 Laboratory Animal Resource Center in Transborder Medical Research Center, and Department of Anatomy and Embryology, Faculty of Medicine, University of Tsukuba, Tsukuba, Japan; 5 Space Biology Laboratory in Transborder Medical Research Center, Faculty of Medicine, University of Tsukuba, Tsukuba, Japan; 6 Department of Management Nutrition, Tokai Gakuin University, Kakamigahara, Japan; 7 Department of Physiology, Gifu University Graduate School of Medicine, Gifu, Japan

Correction to: *Scientific Reports* 10.1038/s41598-021-82013-w, published online 29 January 2021

The original version of this Article contained an error in the value given for the bandwidth of a low-pass filter.

In the Methods section, subheading “Noise removal from the activity continuous”,

“To remove the activity continuous data derived from the EthoVision results, a low-pass filter with 0–1 Hz bandwidth is used (to remove the frequency).”

now reads:

“To remove the activity continuous data derived from the EthoVision results, a low-pass filter with 0–0.2 Hz bandwidth is used (to remove the frequency).”

The original Article has been corrected.

