# Supplementary material for: Study of mouse behavior in different gravity environments
Source: Sci Rep. 2021 Jan 29;11:2665. doi: 10.1038/s41598-021-82013-w (PMC7846607; doi:10.1038/s41598-021-82013-w)
Supplement: Supplementary file 1 — Supplementary Information 1. [file 41598_2021_82013_MOESM1_ESM.pdf]

Study of mouse behavior in different gravity environments

Author list and affiliations

Michihiko Shimomura (1,2), Akane Yumoto (1,2), Naoko Ota-Murakami (3), Takashi Kudo (1,4), Masaki Shirakawa (1,2,5), Satoru Takahashi (1,4), Hironobu Morita (1,6,7), and Dai Shiba* (1,2,5)

1) Mouse Epigenetics Project, ISS/Kibo Experiment, Japan Aerospace Exploration Agency
2) JEM Utilization Center, Human Spaceflight Technology Directorate, Japan Aerospace Exploration Agency
3) Tsukuba Division, Advanced Engineering Services Co., Ltd.
4) Laboratory Animal Resource Center in Transborder Medical Research Center, and Department of Anatomy and Embryology, Faculty of Medicine, University of Tsukuba
5) Space Biology Laboratory in Transborder Medical Research Center, Faculty of Medicine, University of Tsukuba
6) Department of Management Nutrition, Tokai Gakuin University
7) Department of Physiology, Gifu University Graduate School of Medicine

*To whom correspondence should be addressed:

Dai Shiba, JEM Utilization Center, Human Spaceflight Technology Directorate, JAXA, Tsukuba, Ibaraki 305-8505, Japan

E-mail: shiba.dai@jaxa.jp

Supplementary Figure 1. AIS activity based on times of day in hypergravity, microgravity, artificial gravity and ground control experiments.

Activity continuous corresponding to time course data per day obtained using EthoVision in hypergravity (Centrifuge Gondola A-D), artificial gravity (FL AG A1 and A4 cage), microgravity (FL MG M1 and M4 cage), ground control at daytime (D001, D002, D004, D005) and ground control at night-time (N001, N002, N004, N005). The gray blocks indicate the period when the cages were refreshed and dotted blocks indicate missing data.

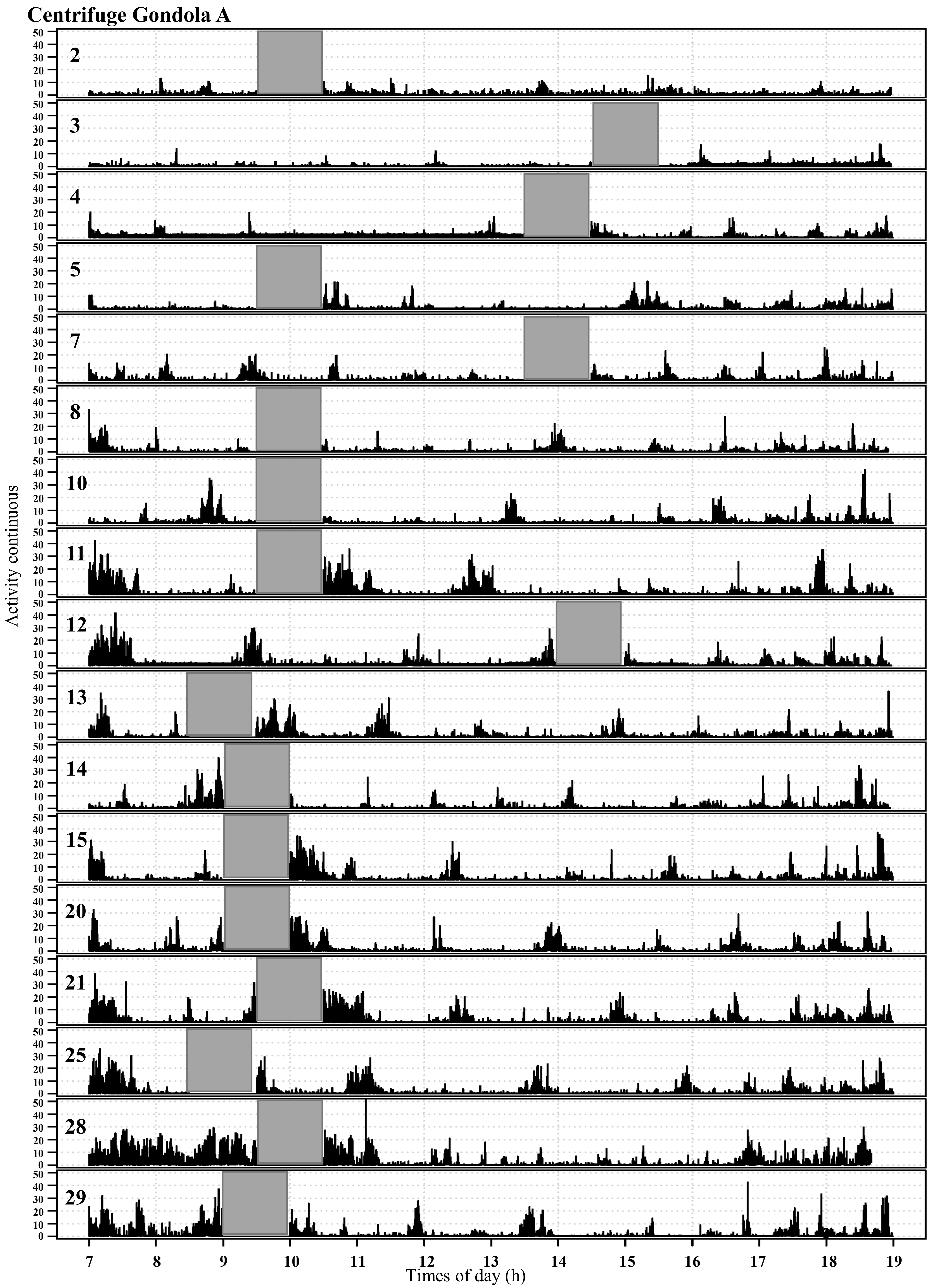

Centrifuge Gondola A
2
3
4
5
7
8
10
11
12
13
14
15
20
21
25
28
29
Activity continuous
Times of day (h)
7
8
9
10
11
12
13
14
15
16
17
18
19
0
10
20
30
40
50

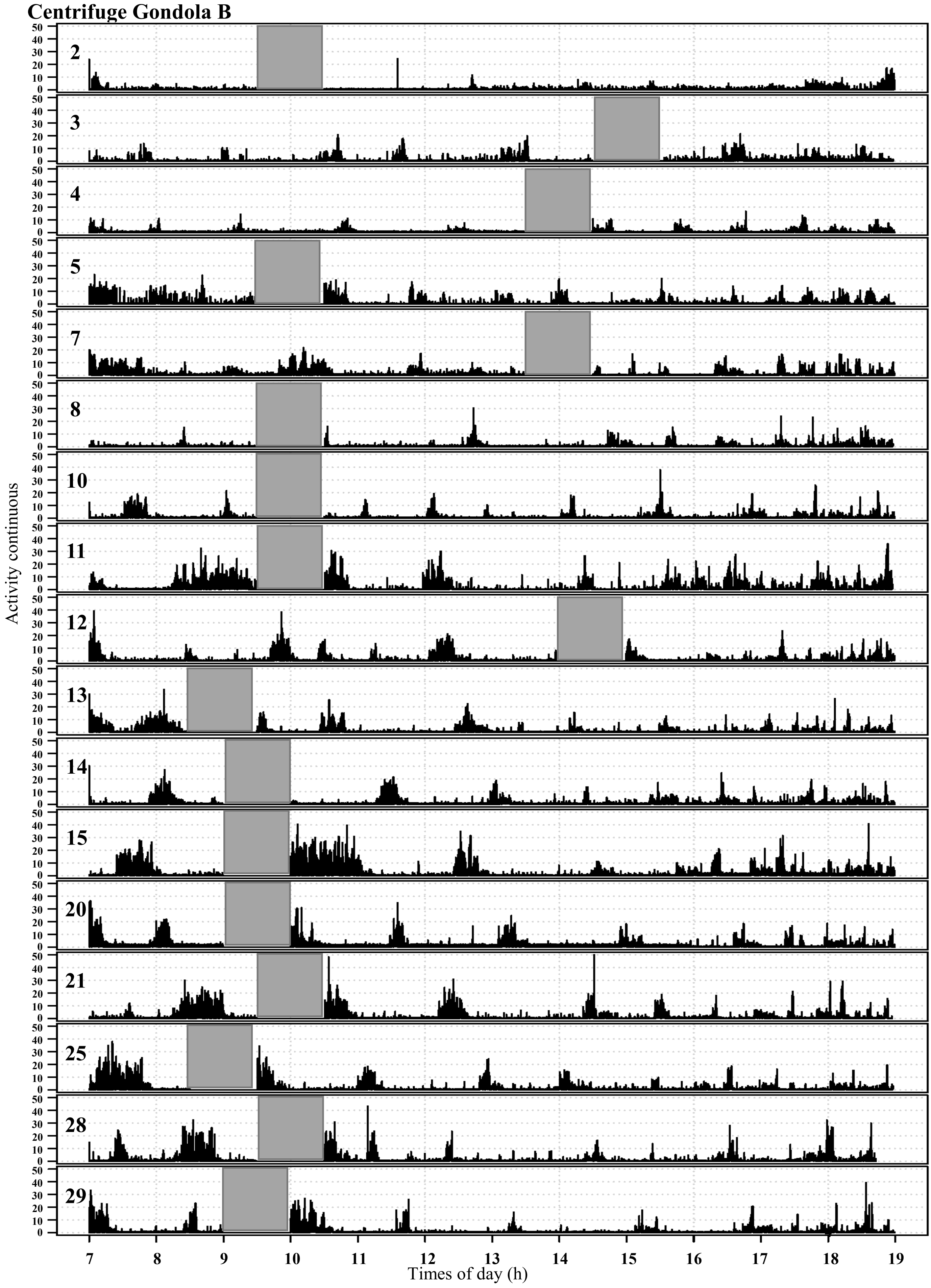

Centrifuge Gondola B
Activity continuous
Times of day (h)
2
3
4
5
7
8
10
11
12
13
14
15
20
21
25
28
29

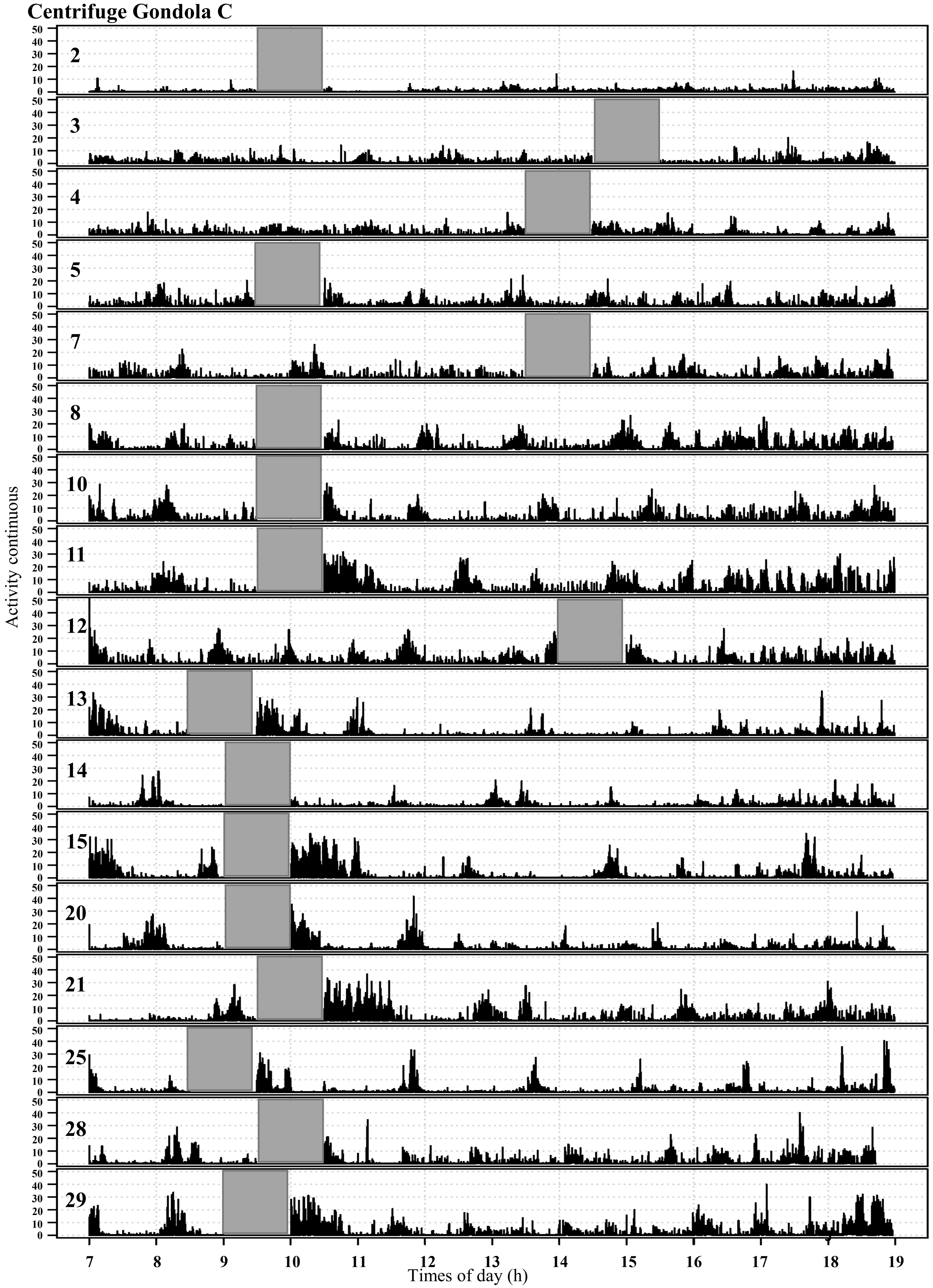

Centrifuge Gondola C
Activity continuous
Times of day (h)
2
3
4
5
7
8
10
11
12
13
14
15
20
21
25
28
29
0
10
20
30
40
50
7
8
9
10
11
12
13
14
15
16
17
18
19

Centrifuge Gondola D

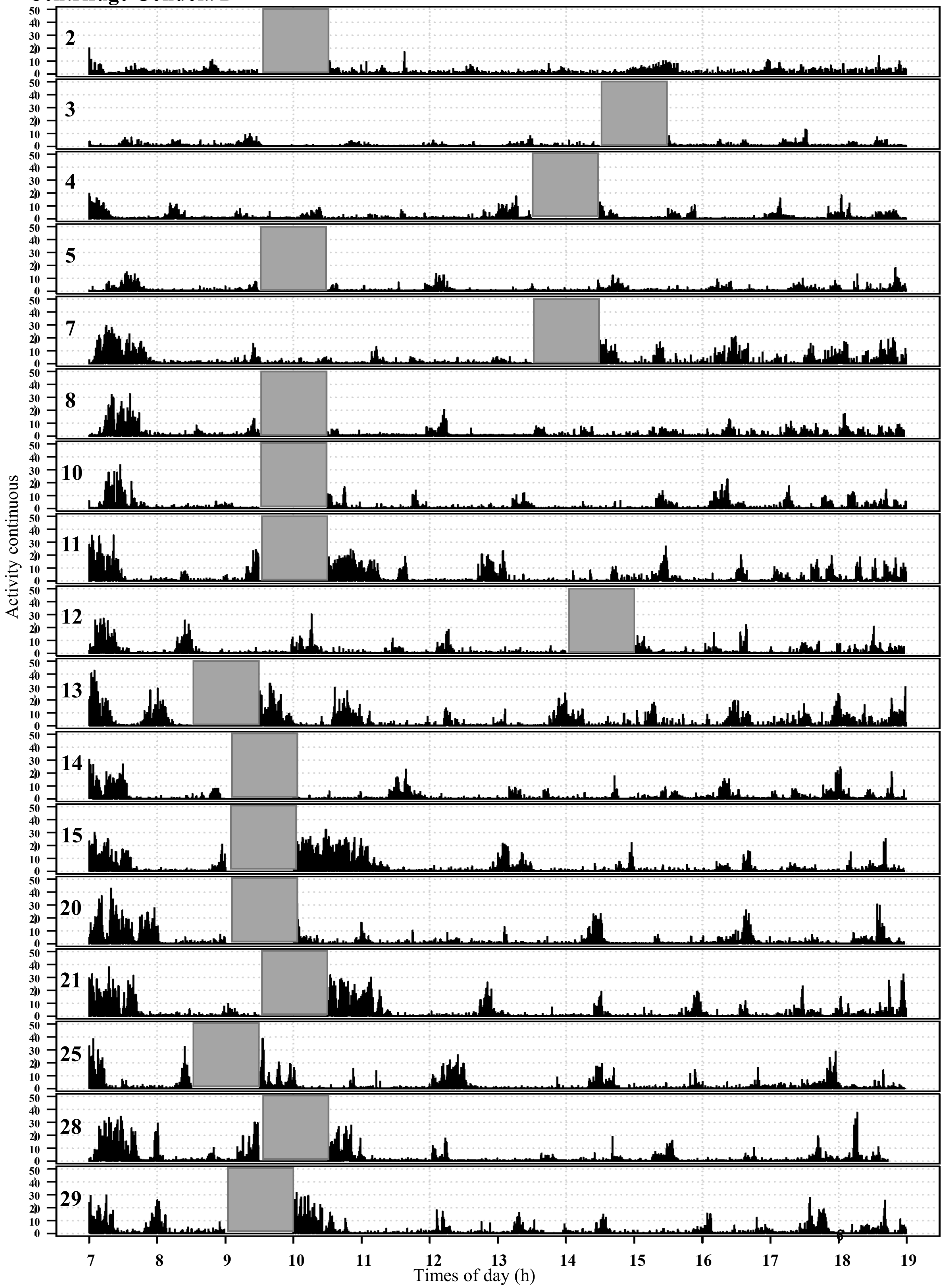

FL AG A1 cage

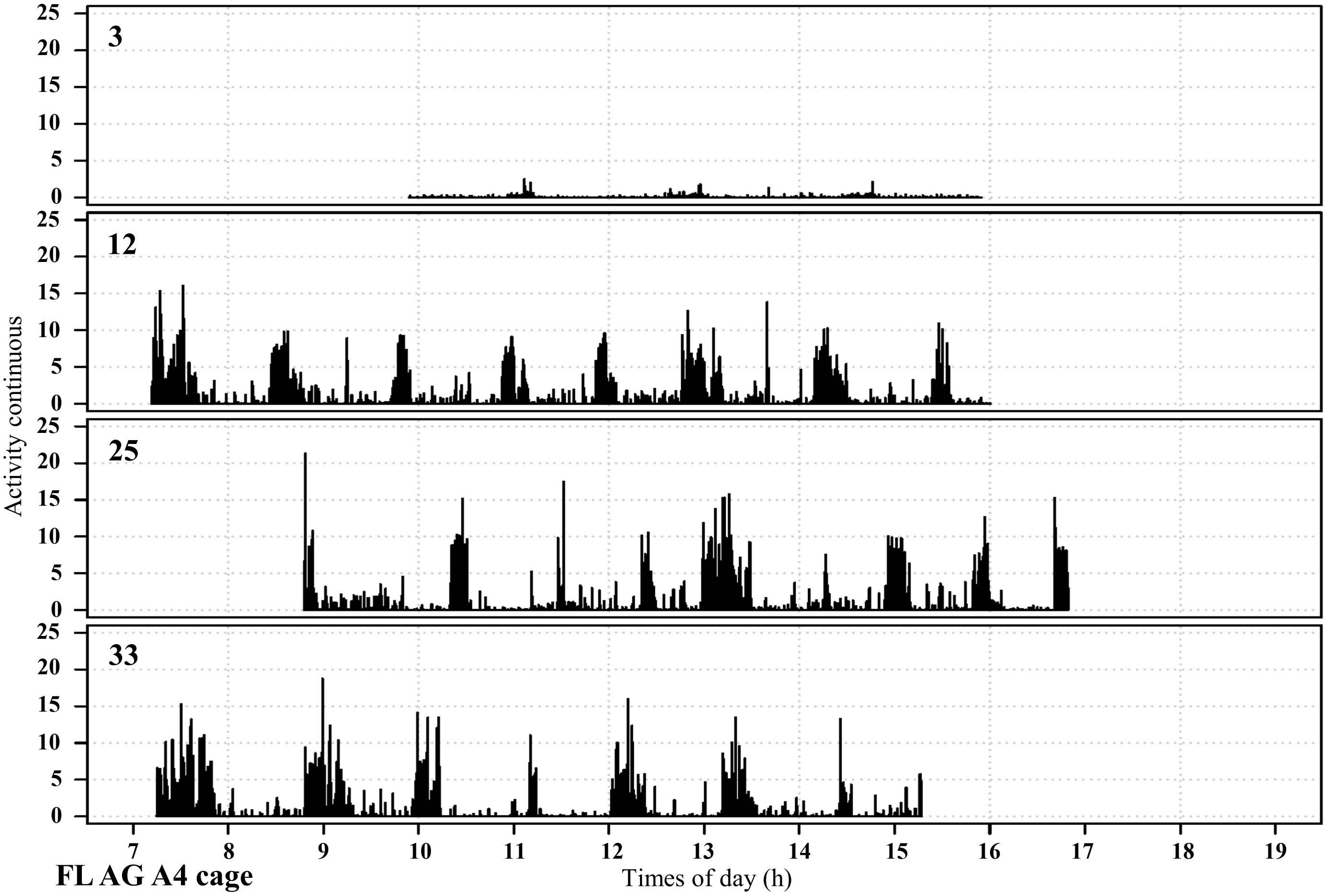

FL AG A4 cage

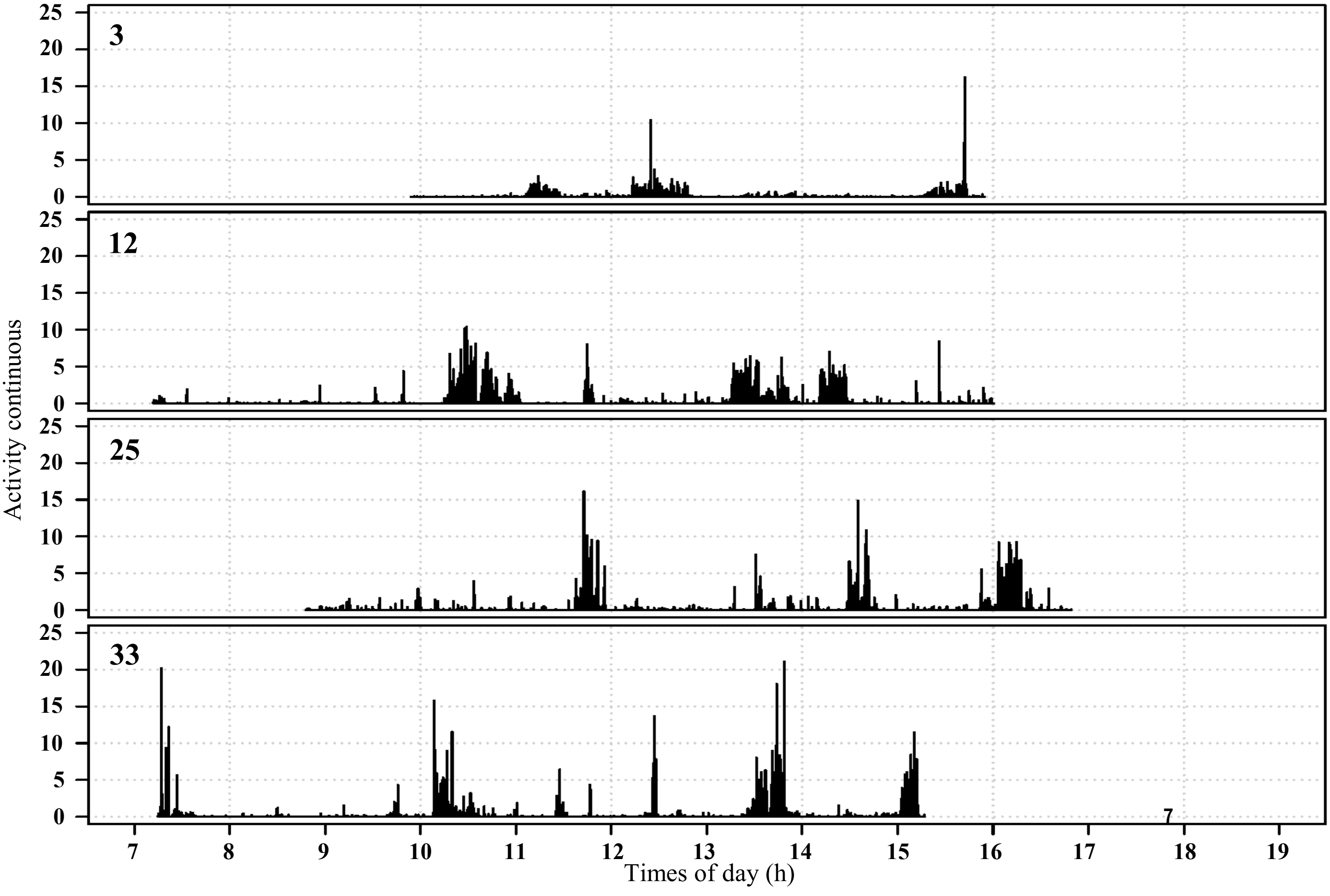

FL MG M1 cage

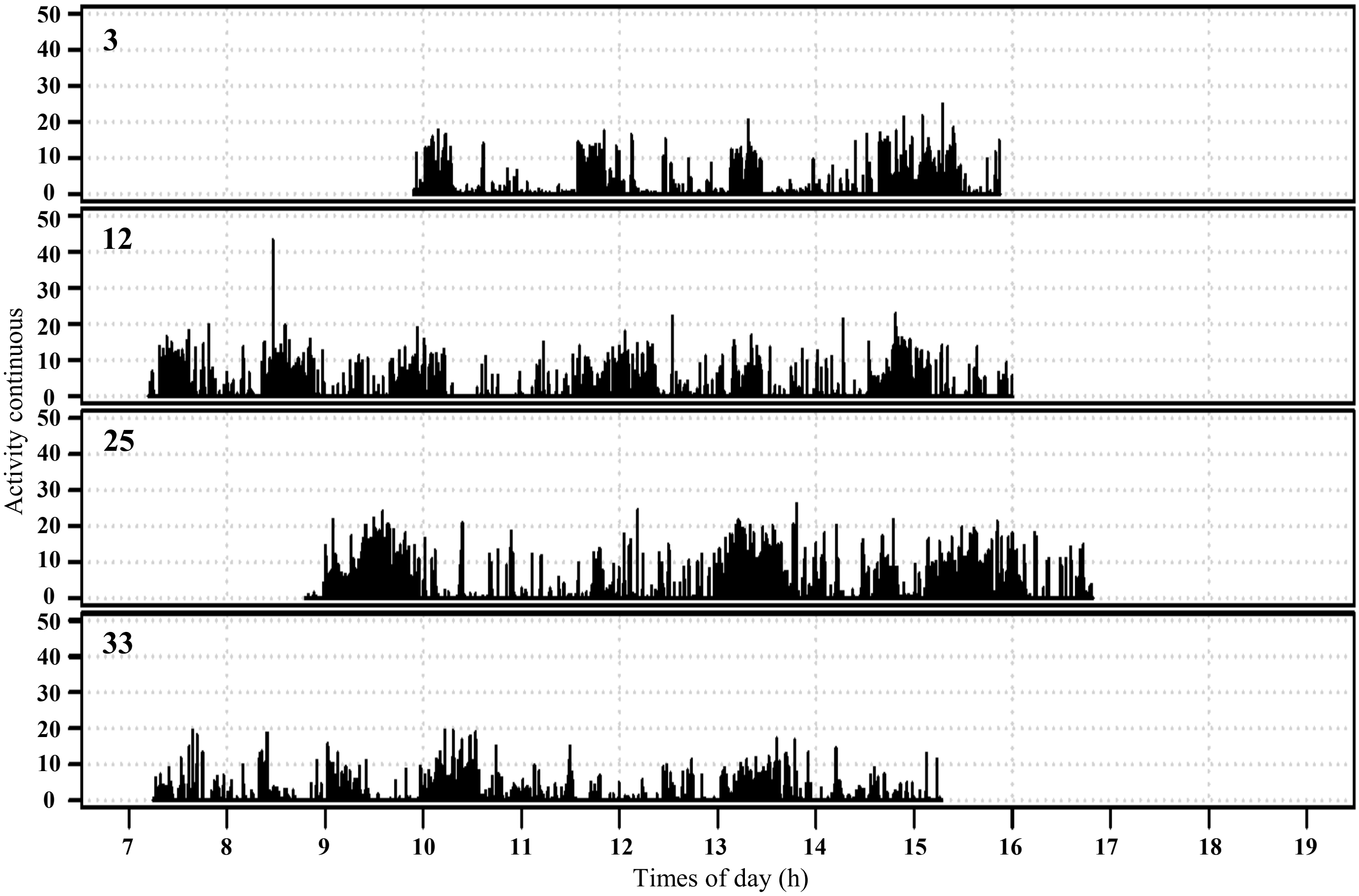

FL MG M4 cage

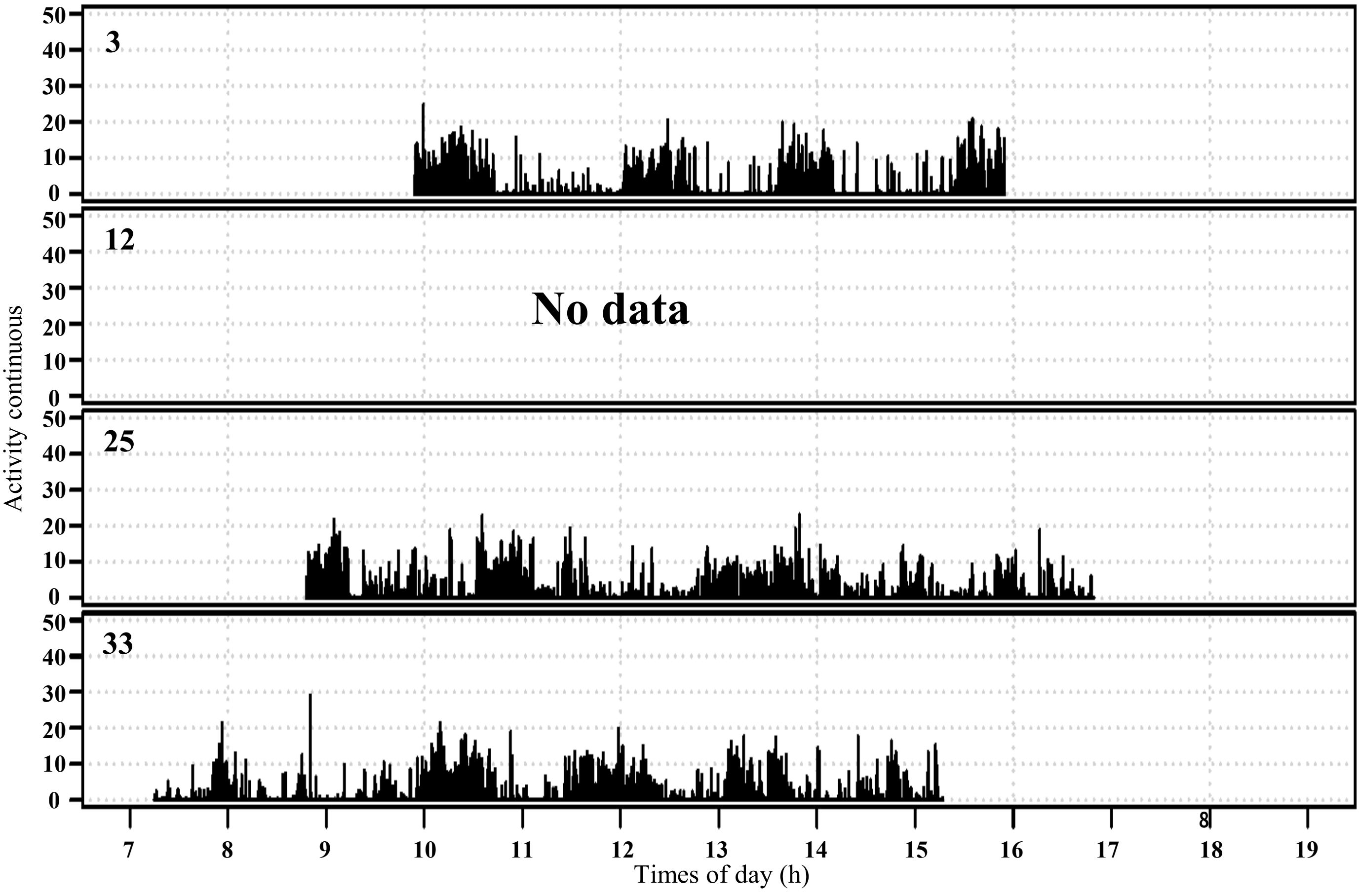

Ground control Cage D001

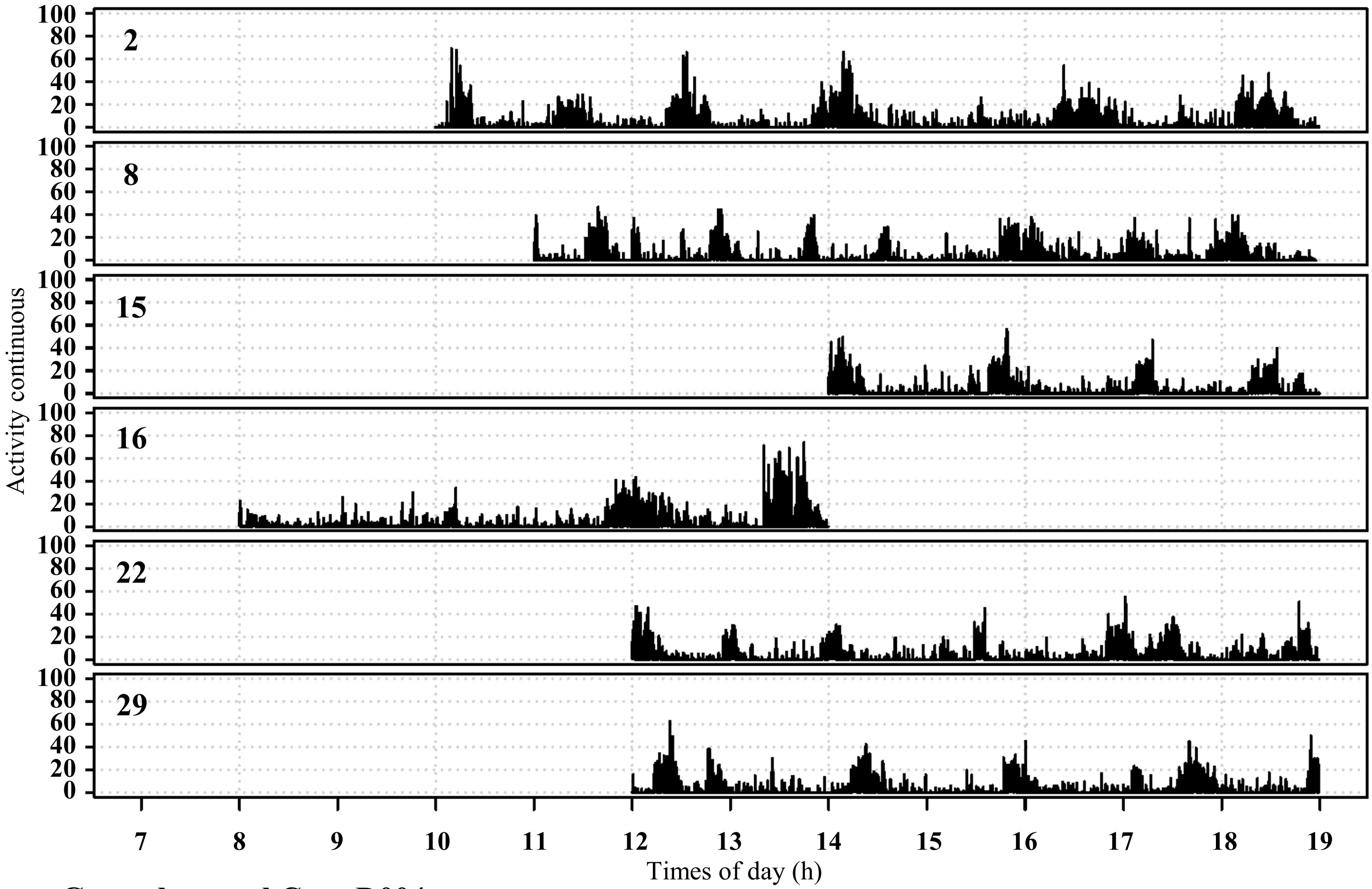

Ground control Cage D004

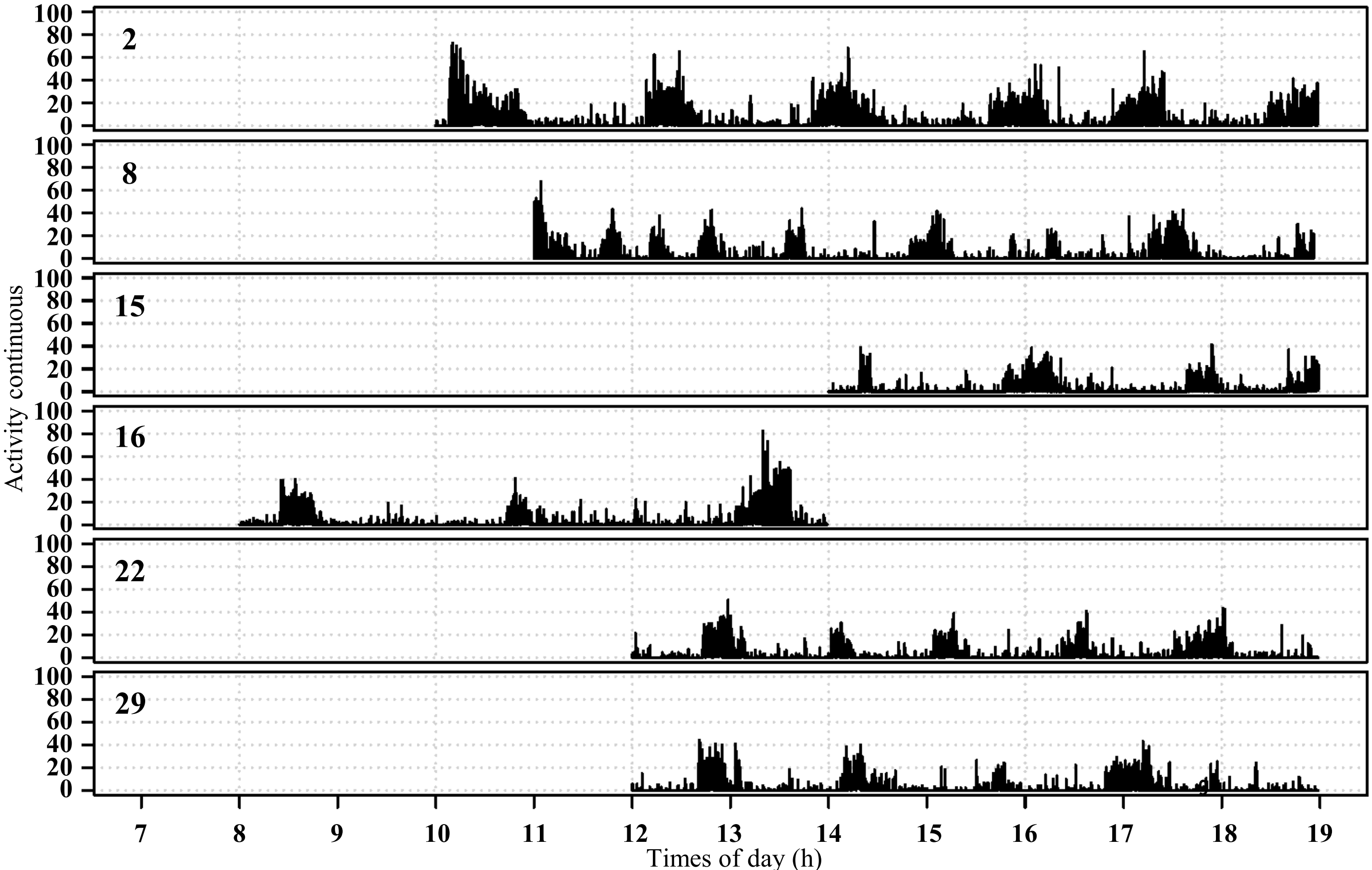

Ground control Cage D002

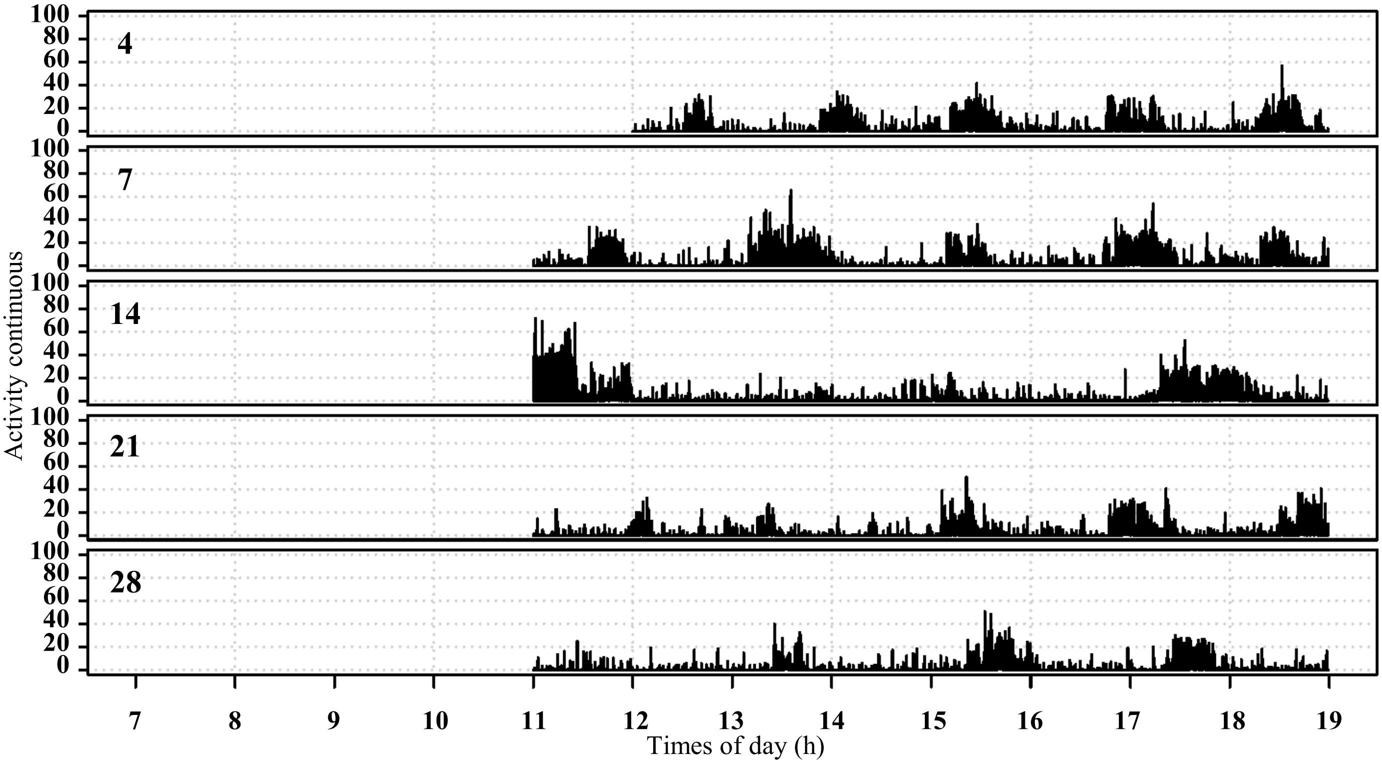

Ground control Cage D005

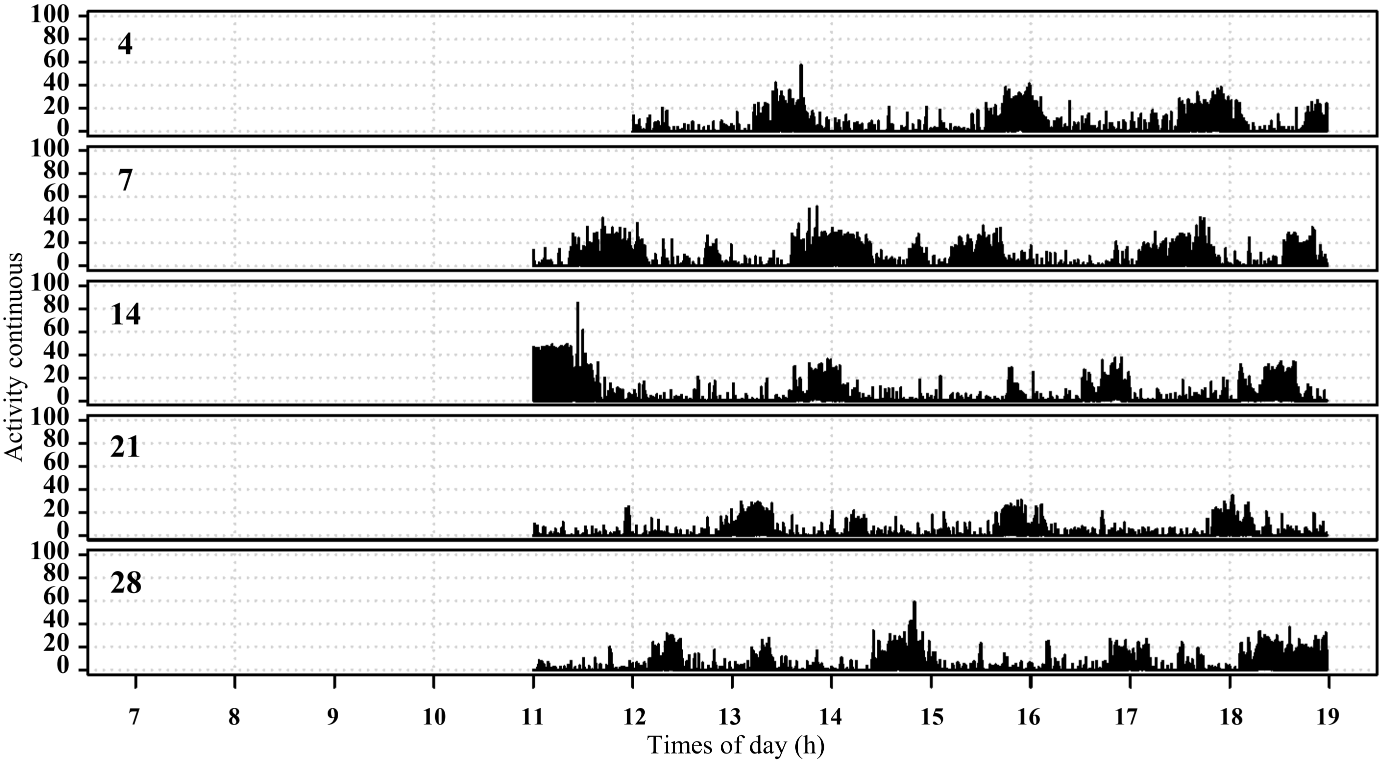

Ground control Cage N001

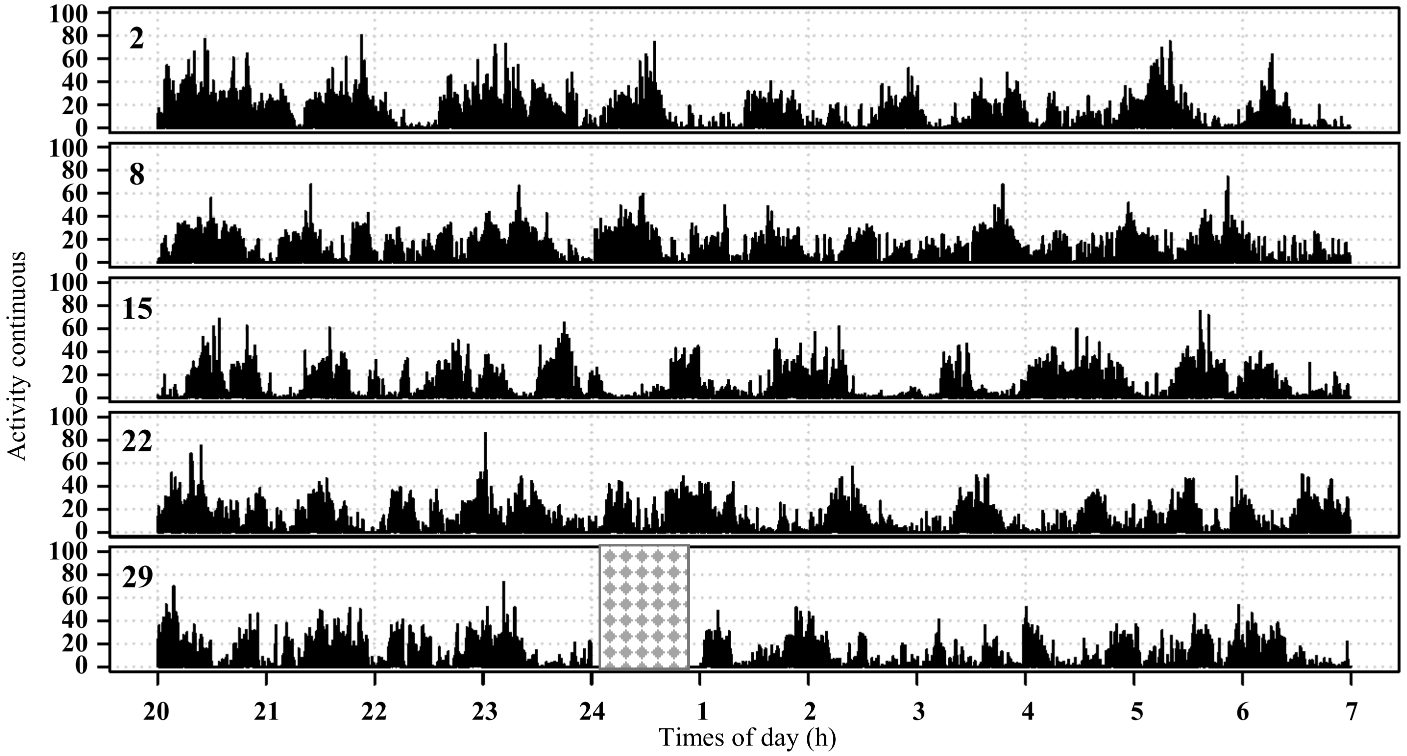

Ground control Cage N004

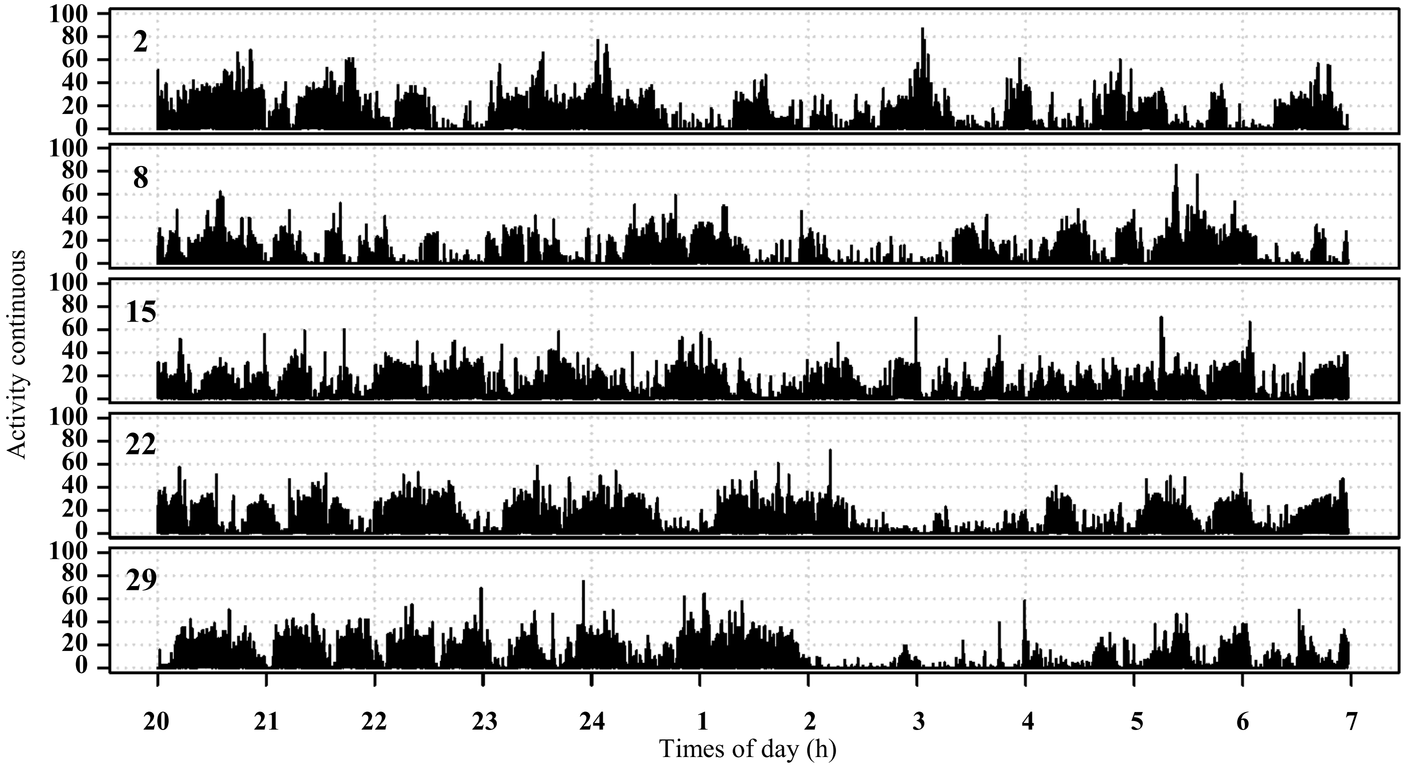

Ground control Cage N002

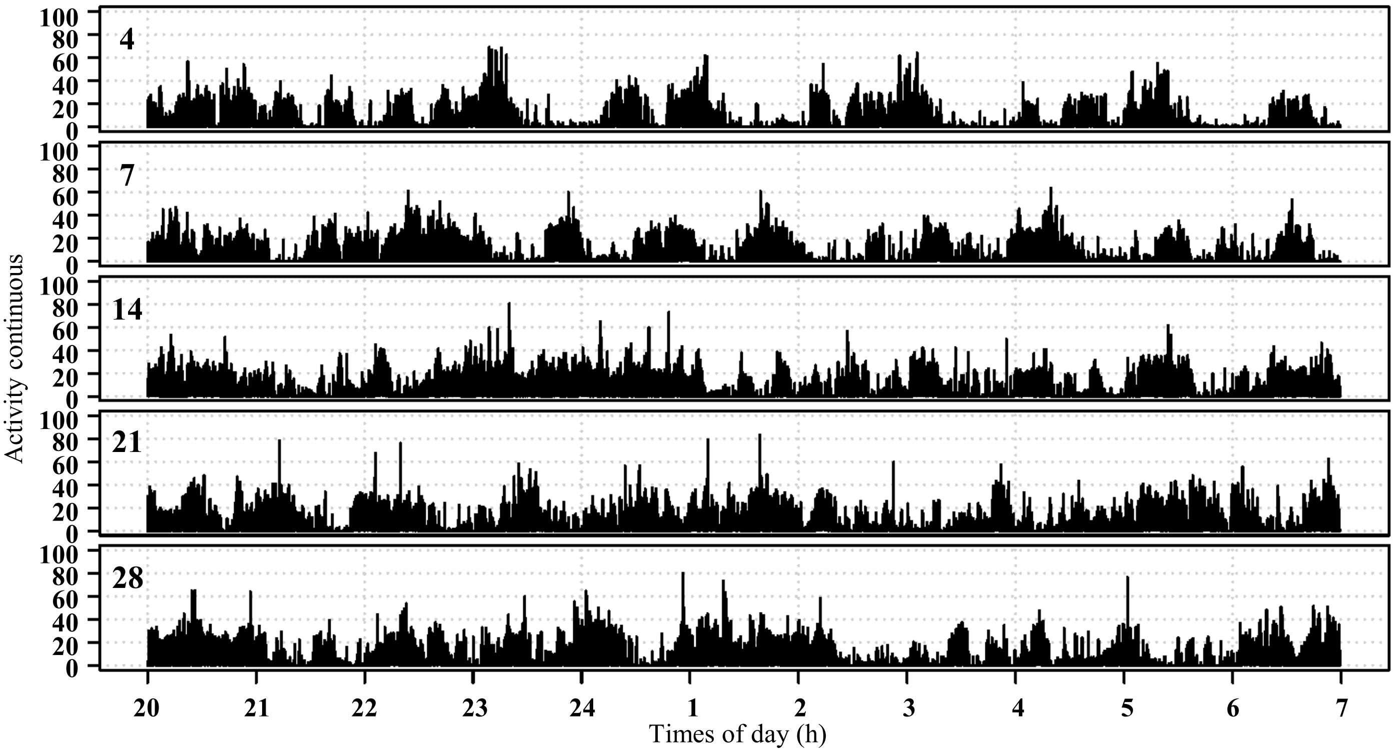

Ground control Cage N005

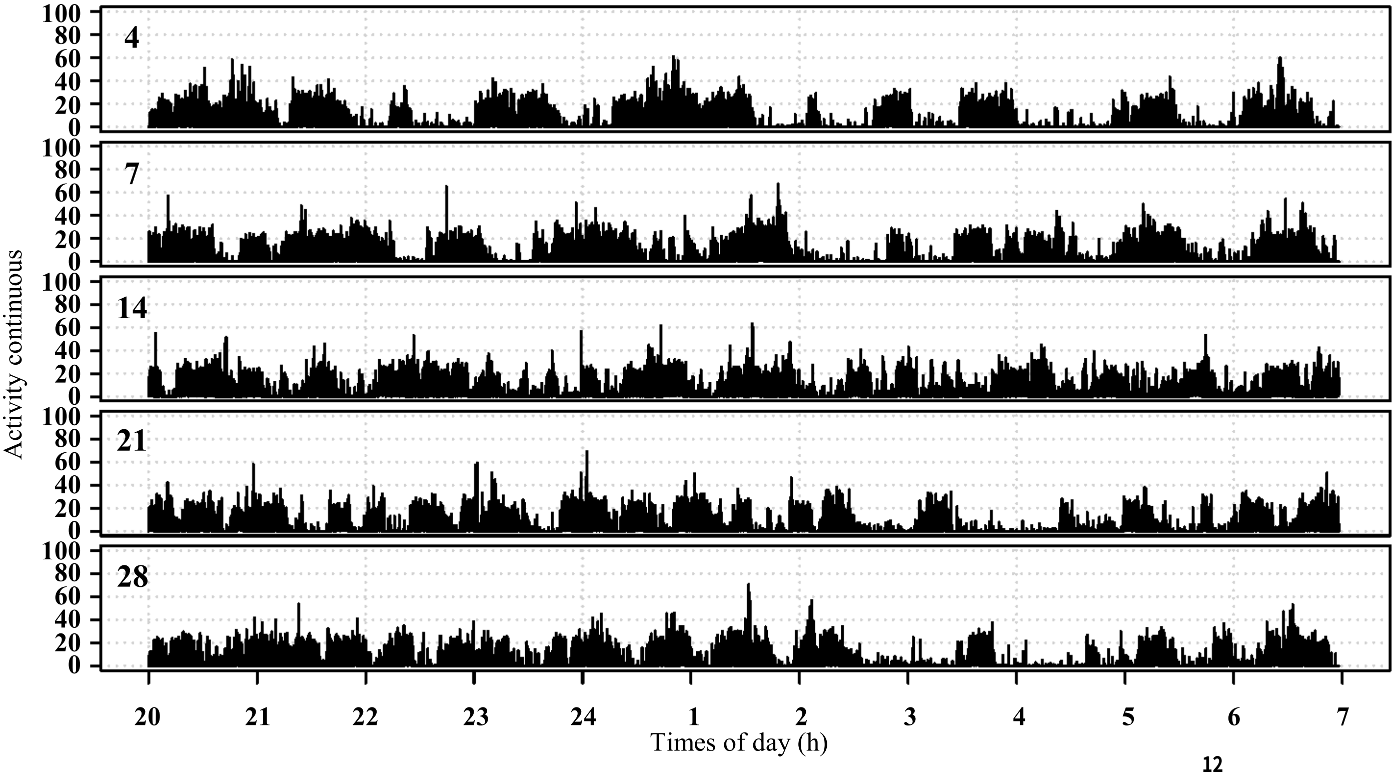

Supplementary Figure 2. Time course and histogram of active continuous

a) Time course of activity continuous

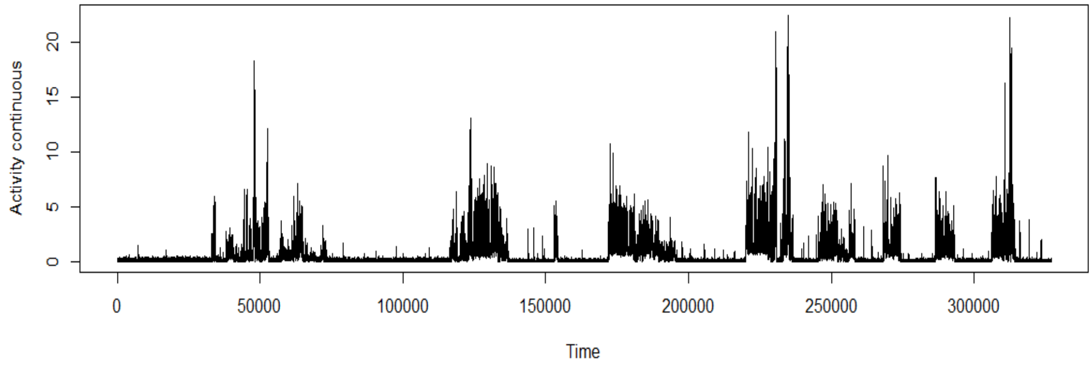

b) Log converted activity continuous (using data before low-pass filter)

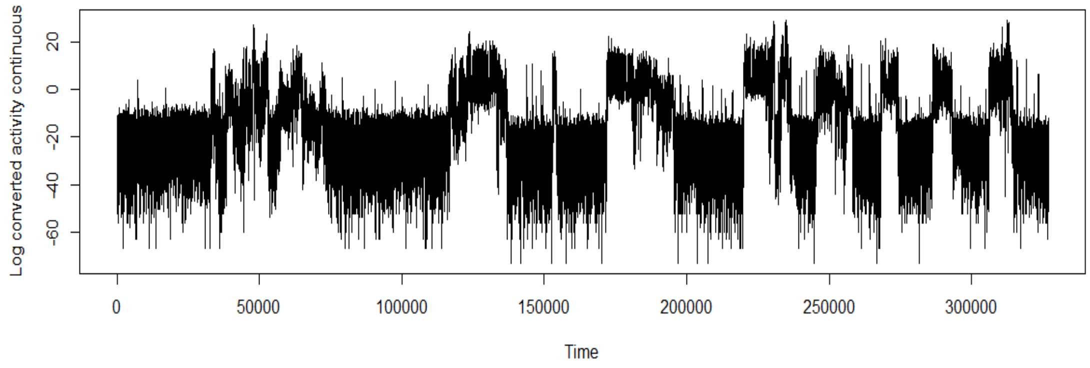

c) Histogram of log converted activity continuous (using data before low-pass filter)

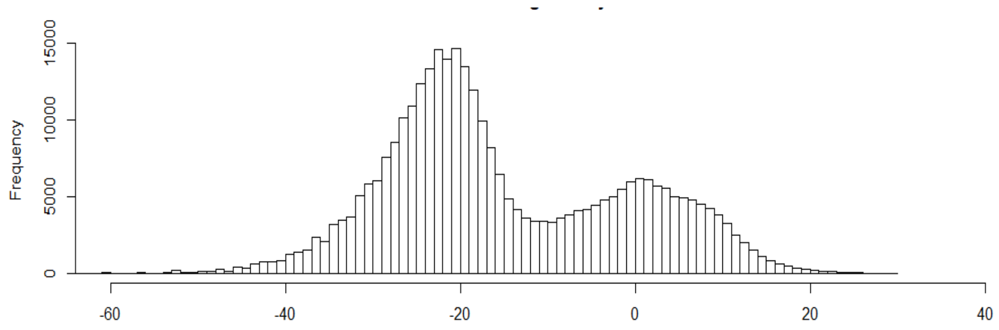

Supplementary Activity amount when sampled from time course data.

Time course data obtained log-converting the activity continuous data after low-pass filter is shown in Fig. S1_1 and a histogram of the data is shown in Fig. S1_2.

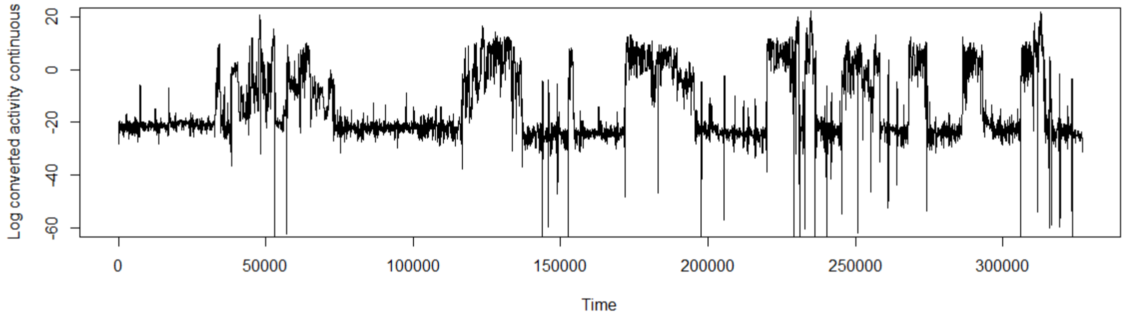

Figure S1_1 Time course obtained log-converting the activity continuous data after low-pass filter (horizontal scale is 1/30 s).

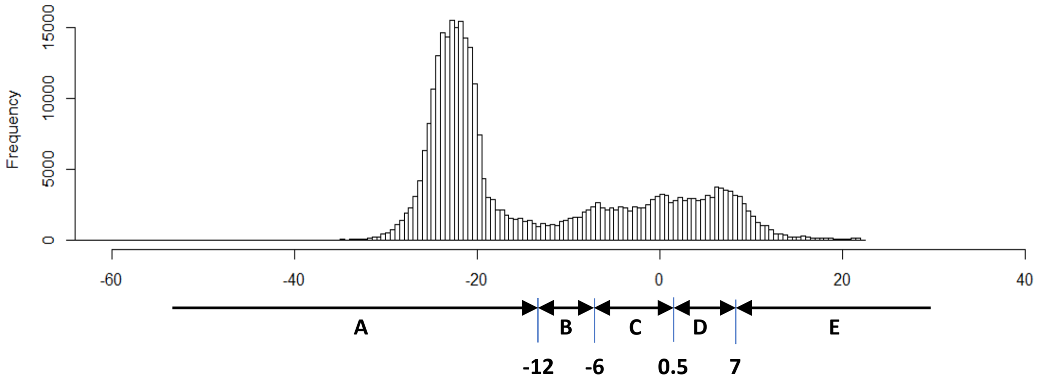

Figure S1_2 Histogram of the data after lo-pass filter

Here, the region is divided into five areas (A< -12, -12≦B<-6, -6≦C<0.5, 0.5≦D<7, 7≦E) as shown in figure S1_2. A summary of the state of movement of mouse is shown in Table S1.

Table S1 Summary of the state of movement

Figure No.	Time region (s)	Behavioral type	Region class	Remarks
**S1_3A1**	2669.8-2736.2	Resting	A< -12	
**S1_3A2**	3423.4-3424.5	Resting	A< -12	
**S1_3A3**	7708.0-7724.2	Resting	A< -12	
**S1_3BC**	9127.6-9128.6	Drinking	-12≦BC<0.5	
**S1_3CD**	5107.5-5109.5	Drinking	-6≦CD<7	Area D: frequent
**S1_3C**	1279.5-1281.2	Grooming	-6≦C<0.5	
**S1_3DE1**	6153.2-6200.2	Grooming	0.5≦DE	Area D: frequent
**S1_3DE2**	10442.0-10449.0	Rearing	0.5≦DE	Area E: frequent

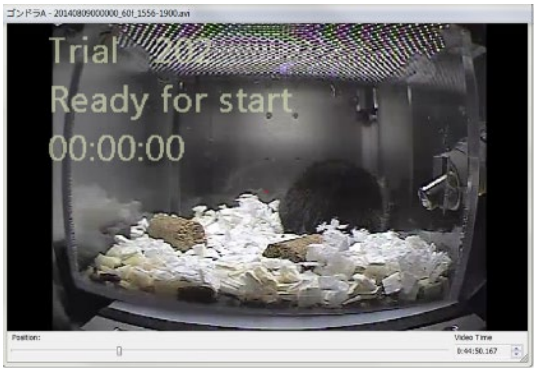

Figure S1_3A1 Resting

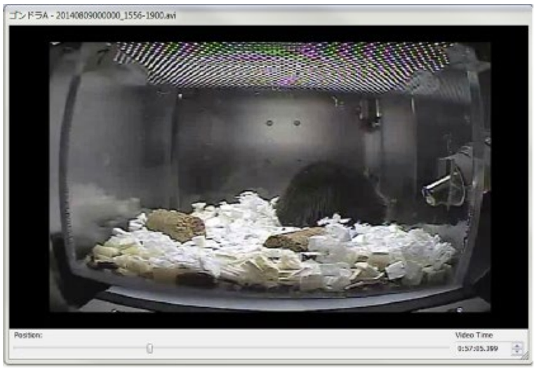
Figure S1_3A2 Resting

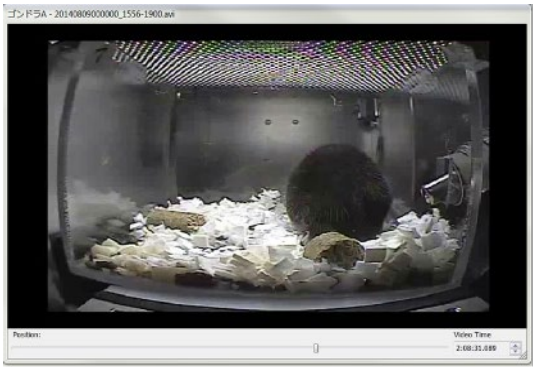
Figure S1_3A3 Resting

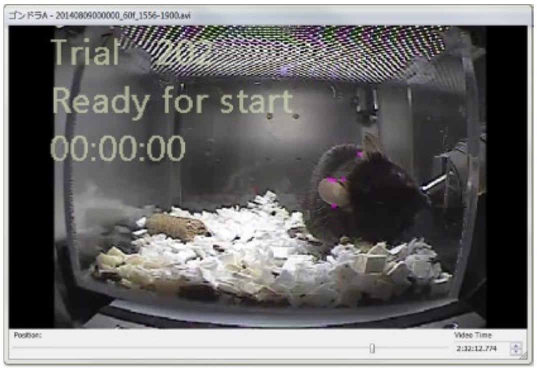

Figure S1_3BC Drinking

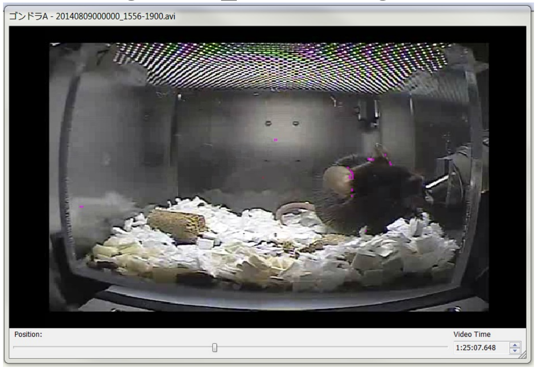
Figure S1_3CD Drinking

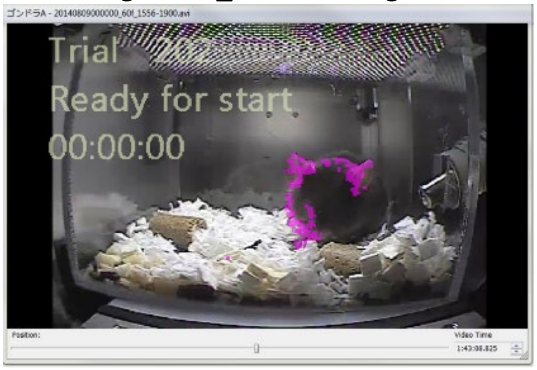

Figure S1_3C Grooming

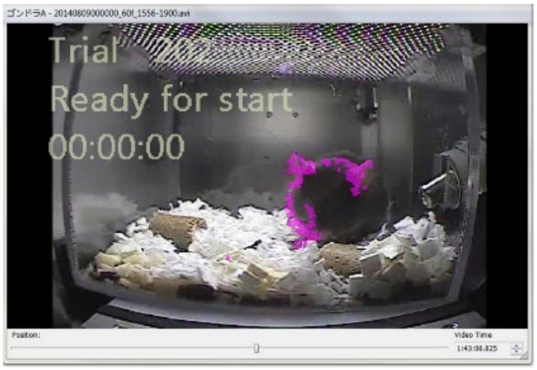

Figure S1_3DE1 Grooming

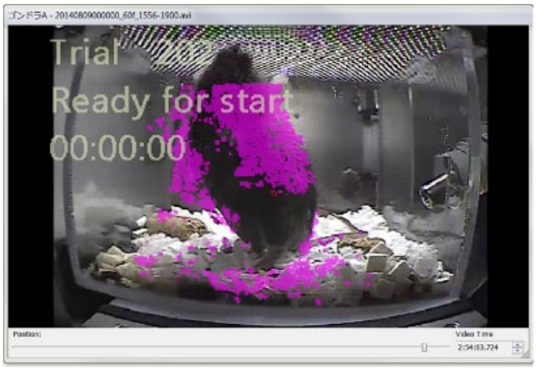

Figure S1_3DE2 Rearing

The areas in pink part indicate movement.

Supplementary Table 1. Video recording of centrifugation on the ground (HG). The bold text indicates the date and time of recording the video.

Elapsed days	Video		Remarks
	Basic recording duration	No recording duration	
1			start centrifugation
**2**		9:30-10:30	
**3**		14:30:15:30	
**4**		13:30-14:30	
**5**		9:30-10:30	
**7**		13:30-14:30	
**8**		9:30-10:30	
**10**		9:30-10:30	
**11**		9:30-10:30	
**12**	7:00-18:59	14:00-15:00	
**13**		8:30-9:30	
**14**		9:00-10:00	
**15**		9:00-10:00	
**20**		9:00-10:00	
**21**		9:30-10:30	
**25**		9:30-10:30	
**28**		9:30-10:30	
**29**		9:00-10:00	
30			end centrifugation

Supplementary Table 2. Video recording and cage refreshment operation for crew in flight (FL). a: add water into feed-water container, b: replace feed container, c: clean-up a cage, d: water leaking, e: change the cage. The bold text indicates the date and time (total time) of recording the video.

Elapsed days	Cage number				Remarks
	A1	A4	M1	M4	
1					TCU to MHU
**3**	✔	✔	✔	✔	video recording 9:54-15:55 (6)
5			✔[a]	✔[a]	cage refreshment
7	✔[a,b]	✔[a,b]	✔[b]	✔[b]	
9	✔[a]	✔[a]	✔[a]	✔[a]	
**12**	✔	✔	✔	✔	video recording 7:12-16:01 (9)
13			✔[a]	✔[a]	cage refreshment
14	✔[a,b]	✔[a,b]	✔[b]	✔[b]	
17	✔[a]	✔[a]	✔[a]	✔[a]	
19			✔[c]	✔[b,c]	
20	✔[b,c]	✔[b,c]			
21			✔[b]		
22				✔[d]	
23				✔[e]	
**25**	✔	✔	✔		video recording 7:59-16:00 (8)
26	✔[a,b,c]	✔[a,b,c]	✔[a]	✔[a]	cage refreshment
27			✔[a,c]	✔[a,c]	
31	✔[a,b]	✔[a,b]	✔[a]	✔[a]	
**33**	✔	✔	✔	✔	video recording 7:15-15:17 (8)
34	✔[a,c]		✔[c]		cage refreshment
36					MHU to TCU

Supplementary Table 3. Video recording and cage refreshment operation on the ground (GC). a: add water into feed-water container, b: feed pellet, c: change deed-water nozzle. The bold text indicates the date and time (total time) of recording the video.

Elapsed days	Cage number				Remarks
	001	002	004	005	
1					start rearing
**2**	✔		✔		**video recording 10:00-18:59 (9), 20:00-06:59 (11)**
**4**		✔		✔	**video recording 12:00-18:59 (7), 20:00-06:59 (11)**
**7**		✔		✔	**video recording 11:00-18:59 (8), 20:00-06:59 (11)**
**8**	✔[a,b]	✔[a,b]	✔[a,b]	✔[a,b]	cage refreshment
	✔		✔		**video recording 11:00-18:59 (8), 20:00-06:59 (11)**
10	✔[a]				
11	✔[a,c]				cage refreshment
**14**				✔[a]	
		✔		✔	**video recording 11:00-18:59 (8), 20:00-06:59 (11)**
**15**	✔[a,b]	✔[a,b]	✔[a,b]	✔[a,b]	cage refreshment
	✔		✔		**video recording 11:00-18:59 (8), 20:00-06:59 (11)**
**16**	✔		✔		**video recording 08:00-13:59 (6)**
20	✔[a]				cage refreshment
**21**		✔		✔	**video recording 11:00-18:59 (8), 20:00-06:59 (11)**
**22**	✔[a,b]	✔[a,b]	✔[a,b]	✔[a,b]	cage refreshment
	✔		✔		**video recording 12:00-18:59 (7), 20:00-06:59 (11)**
**28**		✔		✔	**video recording 11:00-18:59 (8), 20:00-06:59 (11)**
**29**	✔[a,b]	✔[a,b]	✔[a,b]	✔[a,b]	cage refreshment
	✔		✔		**video recording 12:00-18:59 (7), 20:00-06:59 (11)**
36					stop rearing

Videos with a short continuous recording time were not used.

Supplementary AG and MG videos.

The following 4 movies are the recorded A1 of AG cage and M1 of MG cage on the 3rd and 33rd days and converted to 1800x speed using ffmpeg (FFmpeg Developers. (2016). ffmpeg tool (Version be1d324) [Software]. Available from http://ffmpeg.org/).

03A.mov: day-3 AG cage
33A.mov: day-33 AG cage
03M.mov: day-3 MG cage
33M.mov: day-33 MG cage

Supplementary Table 4 Statistic of GC activity ratio per hour.

Daytime

Times of day (h)		t-value	p-value	FDR
12	13	-0.103	0.459684	0.05
12	14	0.171	0.433224	0.025
12	15	-0.774	0.225582	0.00625
12	16	0.5176	0.306452	0.01
12	17	-2.661	0.00915	0.002632
12	18	-1.419	0.087598	0.003846
13	14	0.2881	0.388519	0.016667
13	15	-0.687	0.251267	0.007143
13	16	0.6632	0.258872	0.008333
13	17	-2.651	0.009151	0.002778
13	18	-1.359	0.096571	0.004167
14	15	-1.049	0.154945	0.005
14	16	0.3656	0.359905	0.0125
14	17	-3.142	0.003269	0.0025
14	18	-1.714	0.052933	0.003333
15	16	1.5976	0.065005	0.003571
15	17	-2.236	0.019999	0.002941
15	18	-0.814	0.213998	0.005556
16	17	-4.03	0.000489	0.002381
16	18	-2.256	0.020009	0.003125
17	18	1.146	0.134881	0.004545

Night-time

Times of day (h)		t-value	p-value	FDR
20	1	4.895	0.000117	0.00102
20	2	8.6033	5.83E-07	0.00091
20	3	8.0835	1.25E-06	0.00094
20	4	8.2327	9.12E-07	0.00093
20	5	5.8018	2.45E-05	0.00096
20	6	5.0008	0.000159	0.00106
20	21	4.54	0.000237	0.00111
20	22	3.9538	0.001062	0.00132
20	23	4.348	0.000307	0.00116
20	24	4.3533	0.000247	0.00114
21	1	0.2358	0.408278	0.0125
21	2	3.9239	0.000626	0.00125
21	3	3.542	0.001404	0.00139
21	4	3.5746	0.001297	0.00135
21	5	1.0952	0.144824	0.00313
21	6	1.1177	0.14061	0.00278
21	22	0.4048	0.345759	0.00625
21	23	-0.397	0.348377	0.00714
21	24	-1.105	0.14396	0.00294
22	1	-0.215	0.416254	0.01667
22	2	2.911	0.005259	0.00152
22	3	2.6042	0.00976	0.00167
22	4	2.6123	0.009662	0.00161
22	5	0.4959	0.3137	0.00556
22	6	0.5917	0.281184	0.005
22	23	-0.737	0.236665	0.00385
22	24	-1.303	0.109047	0.00263
23	1	0.6484	0.262975	0.00455
23	2	4.4358	0.000233	0.00109
23	3	4.0314	0.000539	0.00122
23	4	4.0765	0.00048	0.00119
23	5	1.5376	0.071931	0.00227
23	6	1.493	0.078524	0.00238
23	24	-0.7	0.247554	0.00417
24	1	1.4004	0.091416	0.0025
24	2	5.4811	5.42E-05	0.00098
24	3	5.0141	0.000126	0.00104
24	4	5.0936	0.000104	0.001
24	5	2.3796	0.016073	0.00192
24	6	2.1651	0.025584	0.002
1	2	3.7455	0.000919	0.00128
1	3	3.3615	0.002065	0.00147
1	4	3.3919	0.001916	0.00143
1	5	0.8733	0.19772	0.00357
1	6	0.9243	0.185036	0.00333

Times of day (h)		t-value	p-value	FDR
2	3	-0.313	0.379041	0.01
2	4	-0.344	0.36782	0.00833
2	5	-2.893	0.005369	0.00156
2	6	-2.399	0.014569	0.00185
3	4	-0.025	0.490053	0.05
3	5	-2.525	0.011394	0.00179
3	6	-2.082	0.026972	0.00217
4	5	-2.54	0.011006	0.00172
4	6	-2.085	0.02686	0.00208
5	6	0.1662	0.435121	0.025

Yellow is below the p-value upper limit.

Supplementary Table 5 Statistic of HG activity ratio per hour.

Times of day (h)		t-value	p-value	FDR
7	8	2.325351	0.01497019	0.002083
7	11	4.220844	0.00018584	0.001613
7	12	4.016291	0.00029217	0.001667
7	13	5.079951	3.14E-05	0.001351
7	14	4.828128	5.34E-05	0.001389
7	15	3.8669	0.00078334	0.001724
7	16	2.163235	0.02095995	0.0025
7	17	0.4388635	0.3328634	0.01
7	18	-2.320626	0.01520651	0.002174
8	11	1.322892	0.1004507	0.004167
8	12	1.260957	0.1106147	0.004545
8	13	1.872685	0.03891629	0.003125
8	14	1.674394	0.05573186	0.003333
8	15	0.4775526	0.3202412	0.008333
8	16	-0.509547	0.3079575	0.007143
8	17	-2.278565	0.0178752	0.002273
8	18	-4.601924	9.17E-05	0.001515
11	12	-0.01523	0.4939937	0.05
11	13	0.5880036	0.2813857	0.005556
11	14	0.3486004	0.3654056	0.0125
11	15	-1.404755	0.0895126	0.003571
11	16	-2.213667	0.01876584	0.002381
11	17	-4.69011	6.43E-05	0.001429
11	18	-7.28881	1.35E-07	0.001163
12	13	0.5668996	0.2885253	0.00625
12	14	0.3431025	0.3675165	0.016667
12	15	-1.270239	0.1115103	0.005
12	16	-2.081834	0.02463998	0.002778
12	17	-4.36439	0.00015469	0.001563
12	18	-6.867467	3.75E-07	0.00122
13	14	-0.261027	0.3982506	0.025
13	15	-2.444025	0.01243563	0.002
13	16	-3.005152	0.00338307	0.001852
13	17	-5.960849	2.69E-06	0.00125
13	18	-8.636383	1.03E-08	0.001111
14	15	-2.084507	0.02581622	0.002941
14	16	-2.744204	0.00605777	0.001923
14	17	-5.611871	6.17E-06	0.001282
14	18	-8.302496	1.87E-08	0.001136
15	16	-1.403263	0.08985664	0.003846
15	17	-4.729461	7.56E-05	0.001471
15	18	-7.877739	2.77E-07	0.00119
16	17	-2.157346	0.02149542	0.002632
16	18	-4.946505	3.03E-05	0.001316
17	18	-3.375001	0.00142646	0.001786

Yellow is below the p-value upper limit.

Supplementary Table 6 Statistic of GC activity ratio.

Daytime					Night-time				
Elapsed day		t-value	p-value	FDR	Elapsed day		t-value	p-value	FDR
2	4	1.9225	0.1110	0.00167	2	4	5.3829	0.0170	0.00119
2	7	-0.6517	0.2960	0.005	2	7	0.5738	0.3279	0.00417
2	8	2.0636	0.1001	0.00143	2	7	-1.7875	0.1433	0.00185
2	14	3.2663	0.0903	0.00135	2	8	2.1704	0.1122	0.00167
2	15	3.0602	0.0823	0.00122	2	14	-5.6859	0.0416	0.00135
2	21	3.6441	0.0350	0.00111	2	15	1.3949	0.1948	0.00227
2	22	3.1651	0.0823	0.00125	2	21	0.2834	0.4111	0.00455
2	28	1.7048	0.1407	0.00192	2	22	0.6234	0.3110	0.00385
2	29	3.3439	0.0596	0.00116	2	28	0.9796	0.2208	0.00263
4	7	-2.0421	0.1224	0.00179	2	29	7.5948	0.0116	0.00116
4	8	0.2134	0.4254	0.0125	4	8	-0.8475	0.2585	0.00333
4	14	1.7196	0.1571	0.00227	4	14	-11.9968	0.0189	0.00122
4	15	1.4348	0.1599	0.00238	4	15	-5.8923	0.0510	0.00139
4	21	2.4485	0.0745	0.00119	4	21	-1.0908	0.2311	0.00294
4	22	1.5859	0.1483	0.00208	4	22	-2.4807	0.0894	0.00156
4	28	0.8289	0.2723	0.00385	4	28	-3.4745	0.0401	0.00132
4	29	1.9173	0.0999	0.00139	4	29	0.8296	0.2546	0.00313
7	8	2.1423	0.1152	0.00172	7	8	0.9405	0.2261	0.00278
7	14	2.8393	0.1060	0.00161	7	14	-2.4134	0.1233	0.00172
7	15	2.7424	0.1032	0.00156	7	15	-0.1683	0.4469	0.00556
7	21	3.3413	0.0557	0.00114	7	21	-0.0430	0.4853	0.0125
7	22	2.8021	0.1026	0.00152	7	22	-0.0872	0.4695	0.00625
7	28	1.9692	0.1004	0.00147	7	28	-0.0643	0.4784	0.00833
7	29	2.9799	0.0863	0.00132	7	29	2.1809	0.1286	0.00179
8	14	1.3840	0.1901	0.00278	8	14	-4.6970	0.0640	0.00147
8	15	1.1404	0.2010	0.00294	8	15	-1.7700	0.1631	0.002
8	21	2.2518	0.0831	0.00128	8	21	-0.6555	0.3028	0.00357
8	22	1.2805	0.1847	0.00263	8	22	-1.2246	0.1728	0.00208
8	28	0.7474	0.2887	0.00455	8	28	-1.4192	0.1592	0.00192
8	29	1.6519	0.1231	0.00185	8	29	1.3237	0.1947	0.00217
14	15	-0.2251	0.4252	0.01	14	15	21.6413	0.0049	0.00111
14	21	1.7240	0.1620	0.0025	14	21	1.3096	0.2071	0.00238
14	22	-0.0101	0.4966	0.05	14	22	3.1565	0.0943	0.00161
14	28	0.3784	0.3846	0.00714	14	28	5.1667	0.0534	0.00143
14	29	0.8271	0.2710	0.00357	14	29	20.0455	0.0056	0.00114
15	21	1.7231	0.1455	0.002	15	21	0.0453	0.4856	0.01667
15	22	0.1767	0.4381	0.01667	15	22	0.0831	0.4736	0.00714
15	28	0.4114	0.3746	0.00556	15	28	0.2192	0.4311	0.005
15	29	0.8565	0.2466	0.00333	15	29	11.0921	0.0239	0.00125
21	22	-1.6596	0.1546	0.00217	21	22	-0.0104	0.4965	0.025
21	28	-0.2812	0.4084	0.00833	21	28	0.0095	0.4969	0.05
21	29	-1.0884	0.2069	0.00313	21	29	1.2915	0.2075	0.0025
22	28	0.3774	0.3843	0.00625	22	28	0.0440	0.4848	0.01
22	29	0.7483	0.2736	0.00417	22	29	3.0838	0.0850	0.00152
28	29	-0.1820	0.4416	0.025	28	29	4.6610	0.0392	0.00128

Supplementary Table 7 Statistic of HG activity ratio.

Elapsed day		t-value	p-value	FDR
2	3	-1.8195	0.0597	0.00051546
2	4	-3.9766	0.0037	0.00039063
2	5	-4.5474	0.0020	0.00037879
2	7	-4.6792	0.0028	0.00038462
2	8	-5.1405	0.0013	0.00037037
2	10	-7.7199	0.0014	0.00037313
2	11	-7.4743	0.0001	0.00032895
2	12	-7.6434	0.0002	0.00033784
2	13	-5.8083	0.0006	0.00035714
2	14	-9.0506	0.0003	0.00034014
2	15	-6.5325	0.0003	0.00034247
2	20	-8.8659	0.0002	0.00033113
2	21	-7.0481	0.0002	0.00033557
2	25	-7.5800	0.0001	0.0003268
2	26	-5.1912	0.0013	0.00036765
2	28	-5.4392	0.0018	0.00037594
2	29	-6.8982	0.0002	0.00033333
3	4	-2.3761	0.0278	0.00046729
3	5	-3.0221	0.0121	0.00043103
3	7	-3.5103	0.0097	0.00042017
3	8	-3.8104	0.0057	0.0004
3	10	-5.9450	0.0029	0.0003876
3	11	-6.0476	0.0005	0.00035211
3	12	-6.0477	0.0006	0.00035461
3	13	-4.4135	0.0026	0.00038168
3	14	-7.4876	0.0005	0.00034965
3	15	-5.1256	0.0012	0.00036496
3	20	-7.3056	0.0004	0.00034483
3	21	-5.5108	0.0008	0.00035971
3	25	-6.1699	0.0004	0.00034722
3	26	-3.8647	0.0054	0.00039683
3	28	-4.3666	0.0049	0.0003937
3	29	-5.4806	0.0008	0.00036232
4	5	-0.6881	0.2586	0.001
4	7	-1.7565	0.0699	0.00053191
4	8	-1.7395	0.0681	0.00052632
4	10	-2.2662	0.0485	0.0005
4	11	-3.4392	0.0069	0.00040984
4	12	-2.9485	0.0151	0.00043478
4	13	-2.1431	0.0384	0.00048077
4	14	-3.9703	0.0070	0.00041322
4	15	-2.7266	0.0173	0.00044248
4	20	-3.8847	0.0068	0.0004065
4	21	-2.7192	0.0179	0.00044643
4	25	-3.5828	0.0058	0.00040323

Elapsed day		t-value	p-value	FDR
4	26	-1.7948	0.0632	0.00052083
4	28	-2.7219	0.0224	0.00046296
4	29	-2.9988	0.0120	0.00042735
5	7	-1.2148	0.1387	0.00060241
5	8	-1.1081	0.1560	0.00066667
5	10	-1.2285	0.1488	0.00064935
5	11	-2.6383	0.0194	0.00045045
5	12	-2.0283	0.0489	0.00050505
5	13	-1.4521	0.0985	0.00055556
5	14	-2.9252	0.0202	0.00045455
5	15	-1.9929	0.0467	0.00049505
5	20	-2.8651	0.0202	0.00045872
5	21	-1.8811	0.0558	0.0005102
5	25	-2.7857	0.0159	0.0004386
5	26	-1.1631	0.1455	0.00063291
5	28	-2.2053	0.0402	0.00048544
5	29	-2.2388	0.0332	0.0004717
7	8	0.2381	0.4100	0.003125
7	10	0.6191	0.2888	0.00121951
7	11	-0.7466	0.2448	0.00096154
7	12	-0.0934	0.4650	0.00625
7	13	0.0433	0.4835	0.01666667
7	14	-0.6066	0.2905	0.00125
7	15	-0.3329	0.3761	0.00238095
7	20	-0.5954	0.2933	0.00128205
7	21	-0.0884	0.4667	0.00714286
7	25	-0.8720	0.2118	0.00087719
7	26	0.1925	0.4270	0.00384615
7	28	-0.9214	0.1963	0.00080645
7	29	-0.4905	0.3221	0.00147059
8	10	0.3937	0.3591	0.00192308
8	11	-1.1760	0.1439	0.00061728
8	12	-0.4518	0.3363	0.0016129
8	13	-0.2355	0.4109	0.00333333
8	14	-1.0969	0.1686	0.00070423
8	15	-0.6770	0.2622	0.00102041
8	20	-1.0760	0.1715	0.00071429
8	21	-0.4260	0.3438	0.00166667
8	25	-1.3183	0.1195	0.00056818
8	26	-0.0513	0.4804	0.0125
8	28	-1.2299	0.1345	0.00059524
8	29	-0.8665	0.2106	0.00083333
10	11	-2.4269	0.0411	0.0004902
10	12	-1.6622	0.0862	0.00054945
10	13	-0.7887	0.2414	0.00092593

Elapsed day		t-value	p-value	FDR
10	14	-3.7085	0.0078	0.00041667
10	15	-1.5012	0.1103	0.0005618
10	20	-3.4300	0.0113	0.00042373
10	21	-1.3609	0.1260	0.00057471
10	25	-2.6247	0.0341	0.00047619
10	26	-0.4638	0.3360	0.0015625
10	28	-1.7932	0.0834	0.00054348
10	29	-1.8426	0.0758	0.00053763
11	12	1.0668	0.1661	0.00068493
11	13	0.9958	0.1795	0.00075758
11	14	0.3546	0.3696	0.00227273
11	15	0.5261	0.3089	0.00138889
11	20	0.3571	0.3683	0.00217391
11	21	0.9842	0.1820	0.00076923
11	25	-0.1781	0.4322	0.005
11	26	1.1161	0.1553	0.00065789
11	28	-0.4018	0.3529	0.00172414
11	29	0.3304	0.3762	0.0025
12	13	0.1883	0.4292	0.00416667
12	14	-1.0572	0.1673	0.00069444
12	15	-0.3838	0.3585	0.00178571
12	20	-1.0088	0.1769	0.00074627
12	21	0.0025	0.4990	0.05
12	25	-1.2600	0.1304	0.00058824
12	26	0.3863	0.3585	0.00185185
12	28	-1.0815	0.1716	0.00072464
12	29	-0.6372	0.2757	0.00106383
13	14	-0.8917	0.2114	0.00086207
13	15	-0.4658	0.3289	0.00151515
13	20	-0.8700	0.2155	0.00089286
13	21	-0.1745	0.4339	0.00555556
13	25	-1.1498	0.1475	0.00064103
13	26	0.1809	0.4312	0.00454545
13	28	-1.0866	0.1629	0.00067568
13	29	-0.6660	0.2652	0.00104167
14	15	0.3206	0.3820	0.00263158
14	20	0.0147	0.4944	0.025
14	21	0.9068	0.2037	0.00081967
14	25	-0.5731	0.2974	0.00135135
14	26	1.0268	0.1826	0.00078125
14	28	-0.6469	0.2792	0.00111111
14	29	0.0739	0.4722	0.00833333
15	20	-0.3065	0.3867	0.00277778
15	21	0.3593	0.3662	0.00208333
15	25	-0.6897	0.2582	0.00098039

Elapsed day		t-value	p-value	FDR
15	26	0.6203	0.2793	0.00113636
15	28	-0.7552	0.2423	0.0009434
15	29	-0.1975	0.4250	0.00357143
20	21	0.8732	0.2108	0.00084746
20	25	-0.5715	0.2972	0.00131579
20	26	1.0069	0.1857	0.00079365
20	28	-0.6481	0.2784	0.00108696
20	29	0.0634	0.4761	0.01
21	25	-1.1644	0.1449	0.000625
21	26	0.3641	0.3652	0.002
21	28	-1.0481	0.1754	0.00073529
21	29	-0.5927	0.2880	0.00119048
25	26	1.2586	0.1291	0.0005814
25	28	-0.2800	0.3958	0.00294118
25	29	0.4996	0.3176	0.00142857
26	28	-1.1863	0.1422	0.00060976
26	29	-0.8085	0.2256	0.00090909
28	29	0.6213	0.2814	0.00116279

Yellow is below the p-value upper limit.

Supplementary Table 8 Statistic of FL activity ratio.

AG				
Elapsed day		t-value	p-value	FDR
3	12	-5.3001	0.0273	0.01
3	25	-5.9182	0.0506	0.0125
3	33	-7.0219	0.0106	0.0083
12	25	2.7115	0.1114	0.025
12	33	-0.0387	0.4865	0.05
25	33	-4.1249	0.0735	0.0167

MG				
Elapsed day		t-value	p-value	FDR
3	25	-0.1422	0.4529	0.05
3	33	0.6329	0.3015	0.025
25	33	0.8116	0.2773	0.013

Supplementary Table 9.

No.	Strain	Sex	Age	Bins (hours)	Awake/Active (%)			Rest/Immobility/Inactive		Method	Features	Calculation method	Publication year Reference & Remarks
					total	light	dark	(min or sec)	(%)				
				1				316.4894	8.8				
				2				1303.191	36.2				
				3				502.6596	14.0				
				4				781.9149	21.7				
				5				707.4468	19.7				
				6				297.8723	8.3				
				7			66.3	595.7447	16.5				
				8				1601.064	44.5				
				9				1898.936	52.7				
				10				2047.872	56.9				
				11				1675.532	46.5				
1	C57BL/6	male	week6	12	51.2			1787.234	49.6	Video	The activity was analyzed using the HomeCageScan (HCS) software and manual annotation. The software does not identify movements of less than 6 frames out of a 30-frame movie. The identification accuracy of the software is over 80% on average. The estimated amount of activity from WT Rest of Fig. 2A control.	The vertical axis (Time) in Fig. 2A is shown in seconds/hour. Therefore, hour was converted into seconds and calculated as a ratio. Awake / Active (%) was calculated by subtracting averaged Rest/Immobility/Inactive(%) from 100%.	2007 [15]
				13				1340.426	37.2				
				14				2196.809	61.0				
				15				2271.277	63.1				
				16				2829.787	78.6				
				17				2159.574	60.0				
				18		34.7		2773.936	77.1				
				19				2420.213	67.2				
				20				2718.085	75.5				
				21				2755.319	76.5				
				22				2606.383	72.4				
				23				2829.787	48				
2	C57BL/6J	male	week11-14		34				66	Photo beam, Center of grav ity shift (> 0.01m)	To monitor the position of an animal's center of gravity, we placed the Plexiglas enclosures on activity-monitoring platforms, each with a central pivot point and two load beams at the front. The estimated active / wake from Daily time budgets. Black, inactive state of fig 3A.	Awake / Active (%) was calculated by subtracting averaged Rest/Immobility/Inactive(%) from 100%.	2008 fig1A [16]
3	CAST/EiJ		week11-13		62.9			534.6	37.1	Video	Hang, rear, walk, rest and all were classified from an angle perpendicular to the cage using a consumer grade camcorder by SVMHMM (Hidden Markov Model Support Vector Machine) algorithm. Estimated active / wake from rest of fig 6a.	Fig. 6a is the average total resting time for each of the four strains of mice over 24 h (n = 7 animals for each strain). The vertical axis (Total resting time (min)) is shown in min. Therefore, mins was converted into hours and calculated as a ratio. Awake / Active (%) was calculated by subtracting averaged Rest/Immobility/Inactive(%) from 100%.	2010 Figure6.a [12]
	C57BL/6J				68.5			453.8	31.5				
	DBA/2J				60.2			573.1	39.8				
	BTBR		week10-12		57.3			615.4	42.7				
4	C57BL/6	male	month3-9		45.5 ± 22			32.71 ± 1 3.48	54.5 ± 22	EEG/EMG	Sleep/wake behavior were compared with using EEG / EMG and video. Defining sleep as a period of extended immobility (>40s) during which 95% or more of the area of the animal is stationary, a 0.94 correlation with simultaneous EEG/EMG defined sleep were obtained. Estimated active / w.ake from rest time.	Awake / Active (%) was calculated by subtracting averaged Rest/Immobility/Inactive(%) from 100%.	2012 [17] per 1h
					44.7 ± 25			33.19 ± 1 5.41	55.3 ± 25	Video			2012 [17] >40s
5	C57BL/6 × CBA/CaJ		week9		44.4 ± 1.7	38.7 ± 1.6	50.2 ± 2.4			EEG/EMG	Since measuring with using EEG / EMG, wake time was converted to a percentage.		2013 Table 1 [18]
			week12		43.4 ± 1.3	35.5 ± 0.4	50.3 ± 1.7						
			week17		44.5 ± 1.1	40.6 ± 1.8	48.5 ± 2.0						
6	C57BL/6N	male	week20.1 ± 0.3		45.3					EEG/EMG		The vertical axis (W (% of time)) in fig. 6b is shown in %.	2014 fig2B,Standard-diet [19]
7	C57BL/6J	male	week10-20		53.1	36.1	70			EEG/EMG	Since measuring with using EEG / EMG, wake time of daytime and night-time is shown, it was converted to percentage.	The vertical axis (Amount of each stage) in Figure 2B Wake is shown in min/12hr. These minutes were normalized to the ratio in 12 hours	2016 fig2B wake [20]
8	C57BL/6				51.5	30.9	72.1			EEG/EMG		The vertical axis (Total time in wake) in Fig. 1A Wake is shown in min. These minutes were normalized to the ratio in 24 hours, or 12 hours.	2018 fig1A [21]
9	C57BL/6J	male	week8		47.6	32.2 ± 5.3	63.1 ± 4.3			Video		AIS acivity index	GC

Red letters are percentage values converted from rest, Immobility, and inactive.

Supplementary Table 10 Statistic of GC active interval.

Daytime					Night-time				
Elapsed day		t-value	p-value	FDR	Elapsed day		t-value	p-value	FDR
2	4	-1.6634	0.1531	0.0017	2	4	-1.522	0.185	0.0017
2	7	1.0219	0.2419	0.0028	2	7	0.193	0.439	0.01
2	8	-1.2782	0.2089	0.0025	2	8	-0.937	0.225	0.0021
2	14	1.5363	0.1663	0.0019	2	14	0.857	0.264	0.0029
2	15	-1.0507	0.2399	0.0026	2	15	0.812	0.256	0.0028
2	21	-1.0279	0.2433	0.0029	2	21	0.942	0.224	0.0019
2	22	-6.7904	0.0108	0.0011	2	22	-0.640	0.294	0.0036
2	28	2.2701	0.0919	0.0013	2	28	1.054	0.241	0.0025
2	29	-1.5294	0.1664	0.0020	2	29	-0.705	0.293	0.0033
4	7	1.7521	0.1291	0.0014	4	7	170.773	0.000	0.0011
4	8	-0.6288	0.3125	0.0033	4	8	0.357	0.391	0.0056
4	14	2.3935	0.0696	0.0013	4	14	7.638	0.041	0.0012
4	15	-0.4008	0.3731	0.0038	4	15	3.722	0.084	0.0012
4	21	-0.3480	0.3882	0.0045	4	21	3.186	0.097	0.0013
4	22	-1.4096	0.1815	0.0024	4	22	0.475	0.359	0.0042
4	28	2.8810	0.0623	0.0012	4	28	90.453	0.003	0.0011
4	29	0.0793	0.4720	0.0167	4	29	-0.160	0.449	0.0167
7	8	-1.6476	0.1302	0.0014	7	8	-1.688	0.170	0.0017
7	14	-0.1158	0.4605	0.0100	7	14	2.227	0.134	0.0014
7	15	-1.4585	0.1495	0.0016	7	15	1.169	0.225	0.0022
7	21	-1.4464	0.1492	0.0016	7	21	1.214	0.219	0.0019
7	22	-2.7446	0.1055	0.0014	7	22	-1.016	0.247	0.0026
7	28	-0.1528	0.4499	0.0071	7	28	29.006	0.006	0.0012
7	29	-1.6908	0.1337	0.0015	7	29	-0.833	0.279	0.0031
8	14	1.7678	0.1432	0.0015	8	14	2.367	0.104	0.0014
8	15	0.1776	0.4377	0.0056	8	15	2.049	0.092	0.0013
8	21	0.2333	0.4187	0.0050	8	21	2.046	0.089	0.0013
8	22	0.1446	0.4541	0.0083	8	22	0.174	0.440	0.0125
8	28	1.8201	0.1514	0.0017	8	28	2.715	0.112	0.0014
8	29	0.6650	0.3036	0.0031	8	29	-0.264	0.415	0.0063
14	15	-1.5600	0.1611	0.0019	14	15	0.107	0.464	0.05
14	21	-1.5580	0.1597	0.0018	14	21	0.378	0.380	0.0045
14	22	-4.5338	0.0542	0.0012	14	22	-1.571	0.168	0.0016
14	28	-0.0439	0.4849	0.0500	14	28	0.490	0.354	0.0038
14	29	-2.2928	0.0745	0.0013	14	29	-1.101	0.233	0.0024
15	21	0.0520	0.4816	0.0250	15	21	0.246	0.415	0.0071
15	22	-0.1046	0.4667	0.0125	15	22	-1.467	0.154	0.0015
15	28	1.6058	0.1688	0.0022	15	28	0.114	0.464	0.025
15	29	0.4381	0.3622	0.0036	15	29	-1.103	0.225	0.0023
21	22	-0.1846	0.4416	0.0063	21	22	-1.543	0.136	0.0015
21	28	1.6090	0.1677	0.0021	21	28	-0.222	0.430	0.0083
21	29	0.3870	0.3765	0.0042	21	29	-1.180	0.209	0.0018
22	28	6.9354	0.0207	0.0011	22	28	1.765	0.164	0.0016
22	29	1.4905	0.1732	0.0023	22	29	-0.342	0.389	0.005
28	29	-2.7455	0.0676	0.0012	28	29	-1.171	0.225	0.002

Supplementary Table 11 Statistic of HG active interval.

Elapsed day		t-value	p-value	FDR
2	3	1.7156	0.0693	0.0003937
2	4	2.7701	0.0217	0.0003623
2	5	3.5771	0.0109	0.0003571
2	7	4.1737	0.0097	0.0003448
2	8	4.5952	0.0089	0.0003356
2	10	4.2971	0.0076	0.0003289
2	11	4.4872	0.0068	0.0003268
2	12	4.3968	0.0104	0.0003521
2	13	4.3898	0.0090	0.0003378
2	14	4.3891	0.0079	0.0003311
2	15	4.2808	0.0079	0.0003333
2	20	3.9344	0.0111	0.0003597
2	21	4.3907	0.0094	0.0003401
2	25	3.8387	0.0104	0.0003497
2	26	4.1768	0.0098	0.0003472
2	28	3.6400	0.0106	0.0003546
2	29	4.0344	0.0096	0.0003425
3	4	0.3573	0.3692	0.0016667
3	5	0.9760	0.1934	0.0007143
3	7	1.3444	0.1320	0.0004717
3	8	1.6357	0.0990	0.0004132
3	10	1.5029	0.1087	0.0004348
3	11	1.6559	0.0921	0.0004065
3	12	1.4576	0.1199	0.000463
3	13	1.5030	0.1120	0.0004464
3	14	1.5445	0.1053	0.0004274
3	15	1.4774	0.1123	0.0004505
3	20	1.1545	0.1621	0.0005495
3	21	1.4865	0.1148	0.0004545
3	25	1.1256	0.1655	0.0005682
3	26	1.3430	0.1323	0.0004762
3	28	1.0199	0.1843	0.0006579
3	29	1.2638	0.1427	0.000495
4	5	1.1172	0.1540	0.0005155
4	7	1.9770	0.0565	0.0003876
4	8	2.7196	0.0316	0.0003676
4	10	2.2109	0.0385	0.0003731
4	11	2.5269	0.0264	0.000365
4	12	2.3557	0.0469	0.0003846
4	13	2.3479	0.0391	0.0003759
4	14	2.3550	0.0346	0.0003704
4	15	2.1790	0.0408	0.0003788
4	20	1.5718	0.0911	0.0004032
4	21	2.3471	0.0415	0.0003817
4	25	1.4579	0.1011	0.0004202
4	26	1.9804	0.0567	0.0003906
4	28	1.2046	0.1378	0.0004854
4	29	1.7612	0.0698	0.0003968
5	7	0.7722	0.2379	0.0007692
5	8	1.5470	0.1025	0.0004237
5	10	1.1092	0.1563	0.0005263
5	11	1.4564	0.0999	0.0004167
5	12	1.0993	0.1725	0.0006098
5	13	1.1663	0.1512	0.0005051
5	14	1.2290	0.1361	0.0004808
5	15	1.0592	0.1670	0.0005814
5	20	0.3274	0.3782	0.002
5	21	1.1410	0.1582	0.0005376
5	25	0.2780	0.3954	0.0022727
5	26	0.7701	0.2387	0.0007813
5	28	0.0835	0.4681	0.0071429
5	29	0.5802	0.2925	0.0009804
7	8	1.0799	0.1671	0.0005882
7	10	0.5152	0.3130	0.0011905
7	11	0.9642	0.1869	0.0006667
7	12	0.3625	0.3679	0.0016129
7	13	0.5251	0.3094	0.0011628
7	14	0.6467	0.2710	0.0008929
7	15	0.4398	0.3380	0.0014286
7	20	-0.5859	0.2897	0.0009259
7	21	0.4706	0.3281	0.0013514
7	25	-0.5442	0.3038	0.0010417
7	26	-0.0095	0.4963	0.025
7	28	-0.6849	0.2621	0.0008065
7	29	-0.1981	0.4249	0.0033333
8	10	-0.2733	0.3991	0.002381
8	11	0.2369	0.4121	0.0026316
8	12	-1.2743	0.1268	0.0004673
8	13	-0.5313	0.3090	0.0011364
8	14	-0.2114	0.4211	0.003125
8	15	-0.3907	0.3578	0.0015152
8	20	-1.7394	0.0755	0.0004
8	21	-0.7129	0.2526	0.0007937
8	25	-1.4735	0.1083	0.000431
8	26	-1.1128	0.1603	0.0005435
8	28	-1.4703	0.1115	0.0004425
8	29	-1.1618	0.1543	0.0005208
10	11	0.3909	0.3547	0.0014706
10	12	-0.3356	0.3781	0.0019231
10	13	-0.0977	0.4629	0.0055556
10	14	0.0721	0.4725	0.0125
10	15	-0.0801	0.4694	0.01
10	20	-1.0137	0.1755	0.000625
10	21	-0.1749	0.4342	0.0045455
10	25	-0.9403	0.1917	0.0007042
10	26	-0.5281	0.3089	0.0011111
10	28	-1.0333	0.1717	0.0006024
10	29	-0.6517	0.2694	0.0008772
11	12	-0.8829	0.2163	0.0007463
11	13	-0.5644	0.2978	0.001
11	14	-0.3477	0.3700	0.0017241
11	15	-0.4815	0.3236	0.0012821
11	20	-1.4593	0.0979	0.0004098
11	21	-0.6652	0.2682	0.0008621
11	25	-1.3364	0.1151	0.0004587
11	26	-0.9818	0.1831	0.000641
11	28	-1.3857	0.1093	0.0004386
11	29	-1.0667	0.1636	0.0005618
12	13	0.3217	0.3815	0.0020833
12	14	0.4911	0.3254	0.0013158
12	15	0.2393	0.4119	0.0025
12	20	-1.0936	0.1696	0.0005952
12	21	0.2344	0.4124	0.0029412
12	25	-0.9279	0.2065	0.0007353
12	26	-0.3830	0.3608	0.0015625
12	28	-1.0104	0.1900	0.0006944
12	29	-0.5513	0.3069	0.001087
13	14	0.1962	0.4257	0.0035714
13	15	0.0055	0.4979	0.05
13	20	-1.1309	0.1517	0.0005102
13	21	-0.0964	0.4632	0.00625
13	25	-1.0052	0.1802	0.0006329
13	26	-0.5428	0.3036	0.0010204
13	28	-1.0844	0.1664	0.0005747
13	29	-0.6769	0.2632	0.0008333
14	15	-0.1603	0.4390	0.005
14	20	-1.1843	0.1406	0.0004902
14	21	-0.2886	0.3920	0.0021739
14	25	-1.0770	0.1622	0.0005556
14	26	-0.6628	0.2662	0.0008475
14	28	-1.1521	0.1496	0.0005
14	29	-0.7785	0.2330	0.0007576
15	20	-0.9536	0.1889	0.0006849
15	21	-0.0811	0.4693	0.0083333
15	25	-0.8824	0.2059	0.0007246
15	26	-0.4526	0.3338	0.0013889
15	28	-0.9815	0.1836	0.0006494
15	29	-0.5846	0.2901	0.0009434
20	21	1.1119	0.1572	0.0005319
20	25	-0.0314	0.4880	0.0166667
20	26	0.5833	0.2906	0.0009615
20	28	-0.2333	0.4122	0.0027778
20	29	0.3398	0.3729	0.0017857
21	25	-0.9760	0.1888	0.0006757
21	26	-0.4891	0.3218	0.00125
21	28	-1.0569	0.1742	0.0006173
21	29	-0.6330	0.2775	0.0009091
25	26	0.5408	0.3050	0.0010638
25	28	-0.1900	0.4279	0.0041667
25	29	0.3331	0.3752	0.0018519
26	28	-0.6824	0.2630	0.0008197
26	29	-0.1915	0.4274	0.0038462
28	29	0.4938	0.3203	0.0012195

Supplementary Table 12 Statistic of FL active interval.

AG				
Elapsed day		t-value	p-value	FDR
3	12	2.8506	0.0743	0.0083
3	25	-0.6439	0.2963	0.025
3	33	3.1851	0.0894	0.0125
12	25	-2.8208	0.0894	0.01
12	33	0.1505	0.4500	0.05
25	33	3.0019	0.0987	0.0167

MG				
Elapsed day		t-value	p-value	FDR
3	25	-0.0749	0.4753	0.05
3	33	-1.1212	0.2311	0.013
25	33	-1.0819	0.2313	0.025

Supplementary Information on EthoVision execution.

1. Setup

Start EthoVIsion and click "New Default Experiment".
Enter the required data on the Experiment Setting screen.
Click "Arena Settings" and determine the analysis area of video.
Click "Trial Control Setting" and use the default.
Click "Detection Settings".
Enter the following items and click "save Changes" at the end.

- Method: gray scaling
- Activity Settings:
  - Activity Threshold 3 or 17
  - Background noise 1
  - Compression artifact filter on

2. Acquisition

Select "Open Acquisition" from "Acquisition" on the menu bar.
Set "Arena Settings", "Trial Control Settings", and Detection Settings" and press the green button on Acquisition Control screen to start data collection.

3. Analysis

Click "Data Profiles" and use the default.
Click "Analysis output" and press "Calculation" button.
Export "Raw data".

Supplementary Information on AIS Activity analysis R script flow.

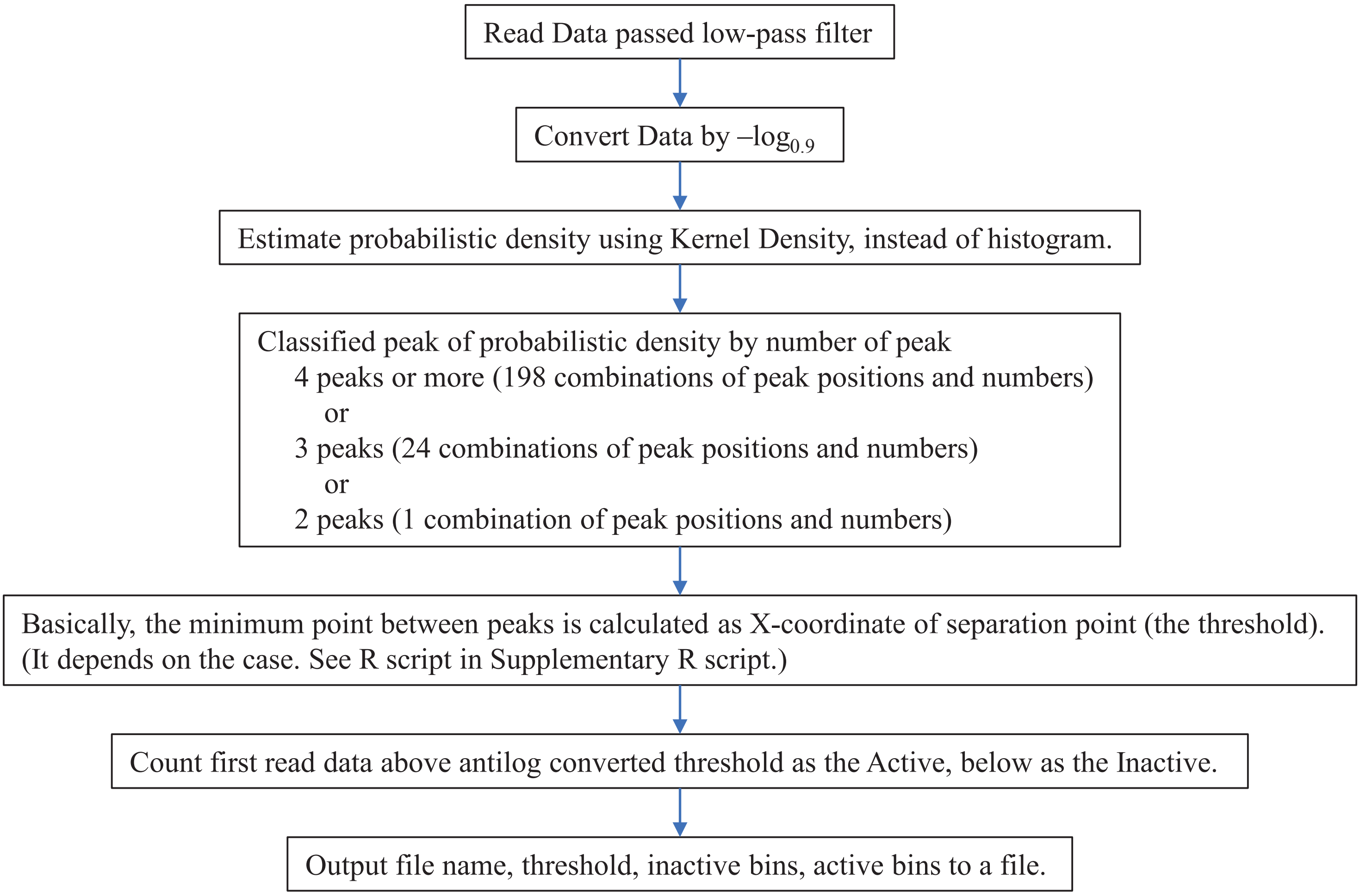

Supplementary R script.

```
### find local maxima subroutine
## obtained from https://stackoverflow.com/questions/34205515/finding-local-maxima-and-minima-in-r

localMaxima <- function(x) {
  # Use -Inf instead if x is numeric (non-integer)
  y <- diff(c(-.Machine$integer.max, x)) > 0L
  rle(y)$lengths
  y <- cumsum(rle(y)$lengths)
  y <- y[seq.int(1L, length(y), 2L)]
  if(x[[1]] == x[[2]]) {
    y <- y[-1]
  }
  y
}

### find local minima

localMinima <- function(x) {
  # Use -Inf instead if x is numeric (non-integer)
  y <- diff(c(.Machine$integer.max, x)) > 0L
  rle(y)$lengths
  y <- cumsum(rle(y)$lengths)
  y <- y[seq.int(1L, length(y), 2L)]
  if(x[[1]] == x[[2]]) {
     y <- y[-1]
  }
  y
}

### main
### edited by Michihiko Shimomura
### copyright JAXA, 2020
### “list.name” is a list that contains the file names for batch processing.
### For example, assuming that the file names of the files in which activity continuous is described are “a1.txt” and “a2.txt”,
### “list.name” file is described as follows:
### a1
### a2
###
### The data is described in the file “a1.txt” in the following format.
### 0.713274
### 0.170416
### 0.002047
### 0.009628
### 0.001609
### 0.379159
### 0.436011
### 0.346797
### <snip>
###
####

listlist <- read.table("list.name", header=F)

pdf("evidence.pdf")

for(mm in 1:length(listlist$V1)){

            minmaxt<-c()
            minmaxty<-c()
            minmaxty1<-c()

            tht<-c()
            th<-c()

            ft1<-0
            ft2<-0
            ft3<-0
            ft4<-0
            ft5<-0
            ft6<-0
            ca<-c()
            dis <- 12
            bai <- 1.2
            sl <-c()

            fil <- paste(listlist$V1[mm],"_r2.txt", sep="")

            x <- scan(fil)
            l <- length(x)
            t <- c(1:l)/30
```

```
		xlog <- -log(abs(x), 0.9)

		y <- data.frame(time=t, va0=x, va1=xlog)

# w$x time, w$y log, w$z real

		d <- density(xlog, kernel=c("gaussian"), window= kernel, n=512)

		plot(d)
		mtext(text=fil)


### find maxima points

		loc.maxxd <- d$x[localMaxima(d$y)]
		loc.maxyd <- d$y[localMaxima(d$y)]
		temax <- cbind(c(loc.maxxd), c(loc.maxyd))

### find minima points

		loc.minxd <- d$x[localMinima(d$y)]
		loc.minyd <- d$y[localMinima(d$y)]
		temin <- cbind(c(loc.minxd), c(loc.minyd))

		temaxA <- cbind(temax, c(1))
		teminA <- cbind(temin, c(0))

### merge max and min points

		minmax <- rbind(temaxA, teminA)
		minmaxt <- minmax[order(minmax[,1], decreasing=F), ,drop=F]

### numbering

		ban <- c(1:nrow(minmaxt))
		minmaxt <- cbind(c(ban), minmaxt)

### arrange in descending order of amplitude (minmaxt[,3])

		minmaxty <- minmaxt[order(minmaxt[,3], decreasing=T), ,drop=F]

### pick up six in descending order of amplitude

		minmaxty1 <- minmaxty[minmaxty[,4] == 1, ,drop=F]
		ft1 <- minmaxty1[1, ,drop=F]
		if(nrow(minmaxty1) >= 2){
			ft2 <- minmaxty1[2, ,drop=F]
		}
		if(nrow(minmaxty1) >= 3){
			ft3 <- minmaxty1[3, ,drop=F]
		}
		if(nrow(minmaxty1) >= 4){
			ft4 <- minmaxty1[4, ,drop=F]
		}
		if(nrow(minmaxty1) >= 5){
			ft5 <- minmaxty1[5, ,drop=F]
		}
		if(nrow(minmaxty1) >= 6){
			ft6 <- minmaxty1[6, ,drop=F]
		}

### categorize peak position

if(nrow(temax) >= 4){


## ft1, ft2, ft3, ft4

		if(ft1[1,1] < ft2[1,1] && ft2[1,1] < ft3[1,1] && ft3[1,1] < ft4[1,1]){
			if(ft1[1,3]/ft2[1,3] < bai && abs(ft1[1,2]-ft2[1,2]) < dis && ft2[1,3]/ft3[1,3] < bai && abs(ft2[1,2]-ft3[1,2]) < dis && ft3[1,3]/ft4[1,3] < bai && abs(ft3[1,2]-ft4[1,2]) < dis){
				ca <- "AA1234"
			} else if(ft2[1,3]/ft3[1,3] < bai && abs(ft2[1,2]-ft3[1,2]) < dis && ft3[1,3]/ft4[1,3] < bai && abs(ft3[1,2]-ft4[1,2]) < dis){
				ca <- "AA234"
			} else if(ft1[1,3]/ft2[1,3] < bai && abs(ft1[1,2]-ft2[1,2]) < dis && ft2[1,3]/ft3[1,3] < bai && abs(ft2[1,2]-ft3[1,2]) < dis){
				ca <- "AA123"
			} else if(ft1[1,3]/ft2[1,3] < bai && abs(ft1[1,2]-ft2[1,2]) < dis && ft3[1,3]/ft4[1,3] < bai && abs(ft3[1,3]-ft4[1,2]) < dis){
				ca <- "AA12_34"
			} else if(ft1[1,3]/ft2[1,3] < bai && abs(ft1[1,2]-ft2[1,2]) < dis){
				ca <- "AA12"
			} else if(ft2[1,3]/ft3[1,3] < bai && abs(ft2[1,2]-ft3[1,2]) < dis){
				ca <- "AA23"
			} else if(ft3[1,3]/ft4[1,3] < bai && abs(ft3[1,2]-ft4[1,2]) < dis){
				ca <- "AA34"
			} else {
				ca <- "AA"
			}
```

```
			}

## ft1, ft2, ft4, ft3

			if(ft1[1,1] < ft2[1,1] && ft2[1,1] < ft4[1,1] && ft4[1,1] < ft3[1,1]){
				if(ft1[1,3]/ft2[1,3] < bai && abs(ft1[1,2]-ft2[1,2]) < dis && ft2[1,3]/ft4[1,3] < bai && abs(ft2[1,2]-ft4[1,2]) < dis &&
ft4[1,3]/ft3[1,3] < bai && abs(ft4[1,2]-ft3[1,2]) < dis){
					ca <- "AB1243"
				} else if(ft2[1,3]/ft4[1,3] < bai && abs(ft2[1,2]-ft4[1,2]) < dis && ft4[1,3]/ft3[1,3] < bai && abs(ft4[1,2]-ft3[1,2]) < dis){
					ca <- "AB243"
				} else if(ft1[1,3]/ft2[1,3] < bai && abs(ft1[1,2]-ft2[1,2]) < dis && ft2[1,3]/ft4[1,3] < bai && abs(ft2[1,2]-ft4[1,2]) < dis){
					ca <- "AB124"
				} else if(ft1[1,3]/ft2[1,3] < bai && abs(ft1[1,2]-ft2[1,2]) < dis && ft4[1,3]/ft3[1,3] < bai && abs(ft4[1,2]-ft3[1,2]) < dis){
					ca <- "AB12_43"
				} else if(ft1[1,3]/ft2[1,3] < bai && abs(ft1[1,2]-ft2[1,2]) < dis){
					ca <- "AB12"
				} else if(ft2[1,3]/ft4[1,3] < bai && abs(ft2[1,2]-ft4[1,2]) < dis){
					ca <- "AB24"
				} else if(ft4[1,3]/ft3[1,3] < bai && abs(ft4[1,2]-ft3[1,2]) < dis){
					ca <- "AB43"
				} else {
					ca <- "AB"
				}
			}

## ft1, ft3, ft2, ff4

			if(ft1[1,1] < ft3[1,1] && ft3[1,1] < ft2[1,1] && ft2[1,1] < ft4[1,1]){
				if(ft1[1,3]/ft3[1,3] < bai && abs(ft1[1,2]-ft3[1,2]) < dis && ft3[1,3]/ft2[1,3] < bai && abs(ft3[1,2]-ft2[1,2]) < dis &&
ft2[1,3]/ft4[1,3] < bai && abs(ft2[1,2]-ft4[1,2]) < dis){
					ca <- "AC1324"
				} else if(ft3[1,3]/ft2[1,3] < bai && abs(ft3[1,2]-ft2[1,2]) < dis && ft2[1,3]/ft4[1,3] < bai && abs(ft2[1,2]-ft4[1,2]) < dis){
					ca <- "AC324"
				} else if(ft1[1,3]/ft3[1,3] < bai && abs(ft1[1,2]-ft3[1,2]) < dis && ft3[1,3]/ft2[1,3] < bai && abs(ft3[1,2]-ft2[1,2]) < dis){
					ca <- "AC132"
				} else if(ft1[1,3]/ft3[1,3] < bai && abs(ft1[1,2]-ft3[1,2]) < dis && ft2[1,3]/ft3[1,3] < bai && abs(ft2[1,3]-ft3[1,2]) < dis){
					ca <- "AC13_24"
				} else if(ft1[1,3]/ft3[1,3] < bai && abs(ft1[1,2]-ft3[1,2]) < dis){
					ca <- "AC13"
				} else if(ft3[1,3]/ft2[1,3] < bai && abs(ft3[1,2]-ft2[1,2]) < dis){
					ca <- "AC32"
				} else if(ft2[1,3]/ft4[1,3] < bai && abs(ft2[1,2]-ft4[1,2]) < dis){
					ca <- "AC24"
				} else {
					ca <- "AC"
				}
			}

## ft1, ft3, ft4, ft2

			if(ft1[1,1] < ft3[1,1] && ft3[1,1] < ft4[1,1] && ft4[1,1] < ft2[1,1]){
				if(ft1[1,3]/ft3[1,3] < bai && abs(ft1[1,2]-ft3[1,2]) < dis && ft3[1,3]/ft4[1,3] < bai && abs(ft3[1,2]-ft4[1,2]) < dis &&
ft4[1,3]/ft2[1,3] < bai && abs(ft4[1,3]-ft2[1,2]) < dis){
					ca <- "AD1342"
				} else if(ft3[1,3]/ft4[1,3] < bai && abs(ft3[1,2]-ft4[1,2]) < dis && ft4[1,3]/ft2[1,3] < bai && abs(ft4[1,2]-ft2[1,2]) < dis){
					ca <- "AD342"
				} else if(ft1[1,3]/ft3[1,3] < bai && abs(ft1[1,2]-ft3[1,2]) < dis && ft3[1,3]/ft4[1,3] < bai && abs(ft3[1,2]-ft4[1,2]) < dis){
					ca <- "AD134"
				} else if(ft1[1,3]/ft3[1,3] < bai && abs(ft1[1,2]-ft3[1,2]) < dis && ft4[1,3]/ft2[1,3] < bai && abs(ft4[1,2]-ft2[1,2]) < dis){
					ca <- "AD13_42"
				} else if(ft1[1,3]/ft3[1,3] < bai && abs(ft1[1,2]-ft3[1,2]) < dis){
					ca <- "AD13"
				} else if(ft4[1,3]/ft2[1,3] < bai && abs(ft4[1,2]-ft2[1,2]) < dis){
					ca <- "AD42"
				} else if(ft3[1,3]/ft4[1,3] < bai && abs(ft3[1,2]-ft4[1,2]) < dis){
					ca <- "AD34"
				} else {
					ca <- "AD"
				}
			}

## ft1, ft4, ft2, ft3

			if(ft1[1,1] < ft4[1,1] && ft4[1,1] < ft2[1,1] && ft2[1,1] < ft3[1,1]){
				if(ft1[1,3]/ft4[1,3] < bai && abs(ft1[1,2]-ft4[1,2]) < dis && ft4[1,3]/ft2[1,3] < bai && abs(ft4[1,2]-ft2[1,2]) < dis &&
ft2[1,3]/ft3[1,3] < bai && abs(ft2[1,2]-ft3[1,2]) < dis){
					ca <- "AE1342"
				} else if(ft4[1,3]/ft2[1,3] < bai && abs(ft4[1,2]-ft2[1,2]) < dis && ft2[1,3]/ft3[1,3] < bai && abs(ft2[1,2]-ft3[1,2]) < dis){
					ca <- "AE423"
				} else if(ft1[1,3]/ft4[1,3] < bai && abs(ft1[1,2]-ft4[1,2]) < dis && ft4[1,3]/ft2[1,3] < bai && abs(ft4[1,2]-ft2[1,2]) < dis){
					ca <- "AE142"
				} else if(ft1[1,3]/ft4[1,3] < bai && abs(ft1[1,2]-ft4[1,2]) < dis && ft2[1,3]/ft3[1,3] < bai && abs(ft2[1,2]-ft3[1,2]) < dis){
					ca <- "AE14_23"
				} else if(ft1[1,3]/ft4[1,3] < bai && abs(ft1[1,2]-ft4[1,2]) < dis){
					ca <- "AE14"
				} else if(ft4[1,3]/ft2[1,3] < bai && abs(ft4[1,2]-ft2[1,2]) < dis){
					ca <- "AE42"
```

```
			} else if(ft2[1,3]/ft3[1,3] < bai && abs(ft2[1,2]-ft3[1,2]) < dis){
				ca <- "AE23"
			} else {
				ca <- "AE"
			}
		}

## ft1, ft4, ft3, ft2

		if(ft1[1,1] < ft4[1,1] && ft4[1,1] < ft3[1,1] && ft3[1,1] < ft2[1,1]){
			if(ft1[1,3]/ft4[1,3] < bai && abs(ft1[1,2]-ft4[1,2]) < dis && ft4[1,3]/ft3[1,3] < bai && abs(ft4[1,2]-ft3[1,2]) < dis && ft3[1,3]/ft2[1,3] < bai && abs(ft3[1,2]-ft2[1,2]) < dis){
				ca <- "AF1432"
			} else if(ft4[1,3]/ft3[1,3] < bai && abs(ft4[1,2]-ft3[1,2]) < dis && ft3[1,3]/ft2[1,3] < bai && abs(ft3[1,2]-ft2[1,2]) < dis){
				ca <- "AF432"
			} else if(ft1[1,3]/ft4[1,3] < bai && abs(ft1[1,2]-ft4[1,2]) < dis && ft4[1,3]/ft3[1,3] < bai && abs(ft4[1,2]-ft3[1,2]) < dis){
				ca <- "AF143"
			} else if(ft1[1,3]/ft4[1,3] < bai && abs(ft1[1,2]-ft4[1,2]) < dis && ft3[1,3]/ft2[1,3] < bai && abs(ft3[1,2]-ft2[1,2]) < dis){
				ca <- "AF14_32"
			} else if(ft1[1,3]/ft4[1,3] < bai && abs(ft1[1,2]-ft4[1,2]) < dis){
				ca <- "AF14"
			} else if(ft4[1,3]/ft3[1,3] < bai && abs(ft4[1,2]-ft3[1,2]) < dis){
				ca <- "AF43"
			} else if(ft3[1,3]/ft2[1,3] < bai && abs(ft3[1,2]-ft2[1,2]) < dis){
				ca <- "AF32"
			} else {
				ca <- "AF"
			}
		}

## ft2, ft1, ft3, ft4

		if(ft2[1,1] < ft1[1,1] && ft1[1,1] < ft3[1,1] && ft3[1,1] < ft4[1,1]){
			if(ft2[1,3]/ft1[1,3] < bai && abs(ft2[1,2]-ft1[1,2]) < dis && ft1[1,3]/ft3[1,3] < bai && abs(ft1[1,2]-ft3[1,2]) < dis && ft3[1,3]/ft4[1,3] < bai && abs(ft3[1,2]-ft4[1,2]) < dis){
				ca <- "BA2134"
			} else if(ft1[1,3]/ft3[1,3] < bai && abs(ft1[1,2]-ft3[1,2]) < dis && ft3[1,3]/ft4[1,3] < bai && abs(ft3[1,2]-ft4[1,2]) < dis){
				ca <- "BA134"
			} else if(ft2[1,3]/ft1[1,3] < bai && abs(ft2[1,2]-ft1[1,2]) < dis && ft1[1,3]/ft3[1,3] < bai && abs(ft1[1,2]-ft3[1,2]) < dis){
				ca <- "BA213"
			} else if(ft2[1,3]/ft1[1,3] < bai && abs(ft2[1,2]-ft1[1,2]) < dis && ft3[1,3]/ft4[1,3] < bai && abs(ft3[1,2]-ft4[1,2]) < dis){
				ca <- "BA21_34"
			} else if(ft2[1,3]/ft1[1,3] < bai && abs(ft2[1,2]-ft1[1,2]) < dis){
				ca <- "BA21"
			} else if(ft1[1,3]/ft3[1,3] < bai && abs(ft1[1,2]-ft3[1,2]) < dis){
				ca <- "BA13"
			} else if(ft3[1,3]/ft4[1,3] < bai && abs(ft3[1,2]-ft4[1,2]) < dis){
				ca <- "BA34"
			} else {
				ca <- "BA"
			}
		}


## ft2, ft3, ft1, ft4

		if(ft2[1,1] < ft3[1,1] && ft3[1,1] < ft1[1,1] && ft1[1,1] < ft4[1,1]){
			if(ft2[1,3]/ft3[1,3] < bai && abs(ft2[1,2]-ft3[1,2]) < dis && ft3[1,3]/ft1[1,3] < bai && abs(ft3[1,2]-ft1[1,2]) < dis && ft1[1,3]/ft4[1,3] < bai && abs(ft1[1,2]-ft4[1,2]) < dis){
				ca <- "BB2314"
			} else if(ft3[1,3]/ft1[1,3] < bai && abs(ft3[1,2]-ft1[1,2]) < dis && ft1[1,3]/ft4[1,3] < bai && abs(ft1[1,2]-ft4[1,2]) < dis){
				ca <- "BB314"
			} else if(ft2[1,3]/ft3[1,3] < bai && abs(ft2[1,2]-ft3[1,2]) < dis && ft3[1,3]/ft1[1,3] < bai && abs(ft3[1,2]-ft1[1,2]) < dis){
				ca <- "BB231"
			} else if(ft2[1,3]/ft3[1,3] < bai && abs(ft2[1,2]-ft3[1,2]) < dis && ft1[1,3]/ft4[1,3] < bai && abs(ft1[1,2]-ft4[1,2]) < dis){
				ca <- "BB23_14"
			} else if(ft2[1,3]/ft3[1,3] < bai && abs(ft2[1,2]-ft3[1,2]) < dis){
				ca <- "BB23"
			} else if(ft3[1,3]/ft1[1,3] < bai && abs(ft3[1,2]-ft1[1,2]) < dis){
				ca <- "BB31"
			} else if(ft1[1,3]/ft4[1,3] < bai && abs(ft1[1,2]-ft4[1,2]) < dis){
				ca <- "BB14"
			} else {
				ca <- "BB"
			}
		}

## ft2, ft3, ft4, ft1

		if(ft2[1,1] < ft3[1,1] && ft3[1,1] < ft4[1,1] && ft4[1,1] < ft1[1,1]){
			if(ft2[1,3]/ft3[1,3] < bai && abs(ft2[1,2]-ft3[1,2]) < dis && ft3[1,3]/ft4[1,3] < bai && abs(ft3[1,2]-ft4[1,2]) < dis && ft4[1,3]/ft1[1,3] < bai && abs(ft4[1,2]-ft1[1,2]) < dis){
				ca <- "BC2341"
			} else if(ft3[1,3]/ft4[1,3] < bai && abs(ft3[1,2]-ft4[1,2]) < dis && ft4[1,3]/ft1[1,3] < bai && abs(ft4[1,2]-ft1[1,2]) < dis){
				ca <- "BC341"
			} else if(ft2[1,3]/ft3[1,3] < bai && abs(ft2[1,2]-ft3[1,2]) < dis && ft3[1,3]/ft4[1,3] < bai && abs(ft3[1,2]-ft4[1,2]) < dis){
				ca <- "BC234"
```

```
			} else if(ft2[1,3]/ft3[1,3] < bai && abs(ft2[1,2]-ft3[1,2]) < dis && ft4[1,3]/ft1[1,3] < bai && abs(ft4[1,2]-ft1[1,2]) < dis){
				ca <- "BC23_41"
			} else if(ft2[1,3]/ft3[1,3] < bai && abs(ft2[1,2]-ft3[1,2]) < dis){
				ca <- "BC23"
			} else if(ft3[1,3]/ft4[1,3] < bai && abs(ft3[1,2]-ft4[1,2]) < dis){
				ca <- "BC34"
			} else if(ft4[1,3]/ft1[1,3] < bai && abs(ft4[1,2]-ft1[1,2]) < dis){
				ca <- "BC41"
			} else {
				ca <- "BC"
			}
		}

## ft2, ft1, ft4, ft3

		if(ft2[1,1] < ft1[1,1] && ft1[1,1] < ft4[1,1] && ft4[1,1] < ft3[1,1]){
			if(ft2[1,3]/ft1[1,3] < bai && abs(ft2[1,2]-ft1[1,2]) < dis && ft1[1,3]/ft4[1,3] < bai && abs(ft1[1,2]-ft4[1,2]) < dis &&
ft4[1,3]/ft3[1,3] < bai && abs(ft4[1,2]-ft3[1,2]) < dis){
				ca <- "BD2143"
			} else if(ft1[1,3]/ft4[1,3] < bai && abs(ft1[1,2]-ft4[1,2]) < dis && ft4[1,3]/ft3[1,3] < bai && abs(ft4[1,2]-ft3[1,2]) < dis){
				ca <- "BD143"
			} else if(ft2[1,3]/ft1[1,3] < bai && abs(ft2[1,2]-ft1[1,2]) < dis && ft1[1,3]/ft4[1,3] < bai && abs(ft1[1,2]-ft4[1,2]) < dis){
				ca <- "BD214"
			} else if(ft2[1,3]/ft1[1,3] < bai && abs(ft2[1,2]-ft1[1,2]) < dis && ft4[1,3]/ft3[1,3] < bai && abs(ft4[1,2]-ft3[1,2]) < dis){
				ca <- "BD21_43"
			} else if(ft2[1,3]/ft1[1,3] < bai && abs(ft2[1,2]-ft1[1,2]) < dis){
				ca <- "BD21"
			} else if(ft1[1,3]/ft4[1,3] < bai && abs(ft1[1,2]-ft4[1,2]) < dis){
				ca <- "BD14"
			} else if(ft4[1,3]/ft3[1,3] < bai && abs(ft4[1,2]-ft3[1,2]) < dis){
				ca <- "BD43"
			} else {
				ca <- "BD"
			}
		}

## ft2, ft4, ft1, ft3

		if(ft2[1,1] < ft4[1,1] && ft4[1,1] < ft1[1,1] && ft1[1,1] < ft3[1,1]){
			if(ft2[1,3]/ft4[1,3] < bai && abs(ft2[1,2]-ft4[1,2]) < dis && ft4[1,3]/ft1[1,3] < bai && abs(ft4[1,2]-ft1[1,2]) < dis &&
ft1[1,3]/ft3[1,3] < bai && abs(ft1[1,2]-ft3[1,2]) < dis){
				ca <- "BE2413"
			} else if(ft4[1,3]/ft1[1,3] < bai && abs(ft4[1,2]-ft1[1,2]) < dis && ft1[1,3]/ft3[1,3] < bai && abs(ft1[1,2]-ft3[1,2]) < dis){
				ca <- "BE413"
			} else if(ft2[1,3]/ft4[1,3] < bai && abs(ft2[1,2]-ft4[1,2]) < dis && ft4[1,3]/ft1[1,3] < bai && abs(ft4[1,2]-ft1[1,2]) < dis){
				ca <- "BE241"
			} else if(ft2[1,3]/ft4[1,3] < bai && abs(ft2[1,2]-ft4[1,2]) < dis && ft1[1,3]/ft3[1,3] < bai && abs(ft1[1,2]-ft3[1,2]) < dis){
				ca <- "BE24_13"
			} else if(ft2[1,3]/ft4[1,3] < bai && abs(ft2[1,2]-ft4[1,2]) < dis){
				ca <- "BE24"
			} else if(ft4[1,3]/ft1[1,3] < bai && abs(ft4[1,2]-ft1[1,2]) < dis){
				ca <- "BE41"
			} else if(ft1[1,3]/ft3[1,3] < bai && abs(ft1[1,2]-ft3[1,2]) < dis){
				ca <- "BE13"
			} else {
				ca <- "BE"
			}
		}

## ft2, ft4, ft3, ft1

		if(ft2[1,1] < ft4[1,1] && ft4[1,1] < ft3[1,1] && ft3[1,1] < ft1[1,1]){
			if(ft2[1,3]/ft4[1,3] < bai && abs(ft2[1,2]-ft4[1,2]) < dis && ft4[1,3]/ft3[1,3] < bai && abs(ft4[1,2]-ft3[1,2]) < dis &&
ft3[1,3]/ft1[1,3] < bai && abs(ft3[1,2]-ft1[1,2]) < dis){
				ca <- "BF2431"
			} else if(ft4[1,3]/ft3[1,3] < bai && abs(ft4[1,2]-ft3[1,2]) < dis && ft3[1,3]/ft1[1,3] < bai && abs(ft3[1,2]-ft1[1,2]) < dis){
				ca <- "BF431"
			} else if(ft2[1,3]/ft4[1,3] < bai && abs(ft2[1,2]-ft4[1,2]) < dis && ft4[1,3]/ft3[1,3] < bai && abs(ft4[1,2]-ft3[1,2]) < dis){
				ca <- "BF243"
			} else if(ft2[1,3]/ft4[1,3] < bai && abs(ft2[1,2]-ft4[1,2]) < dis && ft3[1,3]/ft1[1,3] < bai && abs(ft3[1,2]-ft1[1,2]) < dis){
				ca <- "BF24_31"
			} else if(ft2[1,3]/ft4[1,3] < bai && abs(ft2[1,2]-ft4[1,2]) < dis){
				ca <- "BF24"
			} else if(ft4[1,3]/ft3[1,3] < bai && abs(ft4[1,2]-ft3[1,2]) < dis){
				ca <- "BF43"
			} else if(ft3[1,3]/ft1[1,3] < bai && abs(ft3[1,2]-ft1[1,2]) < dis){
				ca <- "BF31"
			} else {
				ca <- "BF"
			}
		}

## ft3, ft2, ft1, ft4

		if(ft3[1,1] < ft2[1,1] && ft2[1,1] < ft1[1,1] && ft1[1,1] < ft4[1,1]){
			if(ft3[1,3]/ft2[1,3] < bai && abs(ft3[1,2]-ft2[1,2]) < dis && ft2[1,3]/ft1[1,3] < bai && abs(ft2[1,2]-ft1[1,2]) < dis &&
ft1[1,3]/ft4[1,3] < bai && abs(ft1[1,2]-ft4[1,2]) < dis){
```

```
				ca <- "CA3214"
			} else if(ft2[1,3]/ft1[1,3] < bai && abs(ft2[1,2]-ft1[1,2]) < dis && ft1[1,3]/ft4[1,3] < bai && abs(ft1[1,2]-ft4[1,2]) < dis){
				ca <- "CA214"
			} else if(ft3[1,3]/ft2[1,3] < bai && abs(ft3[1,2]-ft2[1,2]) < dis && ft2[1,3]/ft1[1,3] < bai && abs(ft2[1,2]-ft1[1,2]) < dis){
				ca <- "CA321"
			} else if(ft3[1,3]/ft2[1,3] < bai && abs(ft3[1,2]-ft2[1,2]) < dis && ft1[1,3]/ft4[1,3] < bai && abs(ft1[1,2]-ft4[1,2]) < dis){
				ca <- "CA32_14"
			} else if(ft3[1,3]/ft2[1,3] < bai && abs(ft3[1,2]-ft2[1,2]) < dis){
				ca <- "CA32"
			} else if(ft2[1,3]/ft1[1,3] < bai && abs(ft2[1,2]-ft1[1,2]) < dis){
				ca <- "CA21"
			} else if(ft1[1,3]/ft4[1,3] < bai && abs(ft1[1,2]-ft4[1,2]) < dis){
				ca <- "CA14"
			} else {
				ca <- "CA"
			}
		}

## ft3, ft1, ft2, ft4

		if(ft3[1,1] < ft1[1,1] && ft1[1,1] < ft2[1,1] && ft2[1,1] < ft4[1,1]){
			if(ft3[1,3]/ft1[1,3] < bai && abs(ft3[1,2]-ft1[1,2]) < dis && ft1[1,3]/ft2[1,3] < bai && abs(ft1[1,2]-ft2[1,2]) < dis &&
ft2[1,3]/ft4[1,3] < bai && abs(ft2[1,2]-ft4[1,2]) < dis){
				ca <- "CB3124"
			} else if(ft1[1,3]/ft2[1,3] < bai && abs(ft1[1,2]-ft2[1,2]) < dis && ft2[1,3]/ft4[1,3] < bai && abs(ft2[1,2]-ft4[1,2]) < dis){
				ca <- "CB124"
			} else if(ft3[1,3]/ft1[1,3] < bai && abs(ft3[1,2]-ft1[1,2]) < dis && ft1[1,3]/ft2[1,3] < bai && abs(ft1[1,2]-ft2[1,2]) < dis){
				ca <- "CB312"
			} else if(ft3[1,3]/ft1[1,3] < bai && abs(ft3[1,2]-ft1[1,2]) < dis && ft2[1,3]/ft4[1,3] < bai && abs(ft2[1,2]-ft4[1,2]) < dis){
				ca <- "CB31_24"
			} else if(ft3[1,3]/ft1[1,3] < bai && abs(ft3[1,2]-ft1[1,2]) < dis){
				ca <- "CB31"
			} else if(ft1[1,3]/ft2[1,3] < bai && abs(ft1[1,2]-ft2[1,2]) < dis){
				ca <- "CB12"
			} else if(ft2[1,3]/ft4[1,3] < bai && abs(ft2[1,2]-ft4[1,2]) < dis){
				ca <- "CB24"
			} else {
				ca <- "CB"
			}
		}

## ft3, ft1, ft4, ft2

		if(ft3[1,1] < ft1[1,1] && ft1[1,1] < ft4[1,1] && ft4[1,1] < ft2[1,1]){
			if(ft3[1,3]/ft1[1,3] < bai && abs(ft3[1,2]-ft1[1,2]) < dis && ft1[1,3]/ft4[1,3] < bai && abs(ft1[1,2]-ft4[1,2]) < dis &&
ft4[1,3]/ft2[1,3] < bai && abs(ft4[1,2]-ft2[1,2]) < dis){
				ca <- "CC3142"
			} else if(ft1[1,3]/ft4[1,3] < bai && abs(ft1[1,2]-ft4[1,2]) < dis && ft4[1,3]/ft2[1,3] < bai && abs(ft4[1,2]-ft2[1,2]) < dis){
				ca <- "CC142"
			} else if(ft3[1,3]/ft1[1,3] < bai && abs(ft3[1,2]-ft1[1,2]) < dis && ft1[1,3]/ft4[1,3] < bai && abs(ft1[1,2]-ft4[1,2]) < dis){
				ca <- "CC314"
			} else if(ft3[1,3]/ft1[1,3] < bai && abs(ft3[1,2]-ft1[1,2]) < dis && ft4[1,3]/ft2[1,3] < bai && abs(ft4[1,2]-ft2[1,2]) < dis){
				ca <- "CC31_42"
			} else if(ft3[1,3]/ft1[1,3] < bai && abs(ft3[1,2]-ft1[1,2]) < dis){
				ca <- "CC31"
			} else if(ft1[1,3]/ft4[1,3] < bai && abs(ft1[1,2]-ft4[1,2]) < dis){
				ca <- "CC14"
			} else if(ft4[1,3]/ft2[1,3] < bai && abs(ft4[1,2]-ft2[1,2]) < dis){
				ca <- "CC42"
			} else {
				ca <- "CC"
			}
		}

## ft3, ft2, ft4, ft1

		if(ft3[1,1] < ft2[1,1] && ft2[1,1] < ft4[1,1] && ft4[1,1] < ft1[1,1]){
			if(ft3[1,3]/ft2[1,3] < bai && abs(ft3[1,2]-ft2[1,2]) < dis && ft2[1,3]/ft4[1,3] < bai && abs(ft2[1,2]-ft4[1,2]) < dis &&
ft4[1,3]/ft1[1,3] < bai && abs(ft4[1,2]-ft1[1,2]) < dis){
				ca <- "CD3142"
			} else if(ft2[1,3]/ft4[1,3] < bai && abs(ft2[1,2]-ft4[1,2]) < dis && ft4[1,3]/ft1[1,3] < bai && abs(ft4[1,2]-ft1[1,2]) < dis){
				ca <- "CD241"
			} else if(ft3[1,3]/ft2[1,3] < bai && abs(ft3[1,2]-ft2[1,2]) < dis && ft2[1,3]/ft4[1,3] < bai && abs(ft2[1,2]-ft4[1,2]) < dis){
				ca <- "CD324"
			} else if(ft3[1,3]/ft2[1,3] < bai && abs(ft3[1,2]-ft2[1,2]) < dis && ft4[1,3]/ft1[1,3] < bai && abs(ft4[1,2]-ft1[1,2]) < dis){
				ca <- "CD32_41"
			} else if(ft3[1,3]/ft2[1,3] < bai && abs(ft3[1,2]-ft2[1,2]) < dis){
				ca <- "CD32"
			} else if(ft2[1,3]/ft4[1,3] < bai && abs(ft2[1,2]-ft4[1,2]) < dis){
				ca <- "CD24"
			} else if(ft4[1,3]/ft1[1,3] < bai && abs(ft4[1,2]-ft1[1,2]) < dis){
				ca <- "CD41"
			} else {
				ca <- "CD"
			}
		}
```

```
## ft3, ft4, ft2, ft1

	if(ft3[1,1] < ft4[1,1] && ft4[1,1] < ft2[1,1] && ft2[1,1] < ft1[1,1]){
		if(ft3[1,3]/ft4[1,3] < bai && abs(ft3[1,2]-ft4[1,2]) < dis && ft4[1,3]/ft2[1,3] < bai && abs(ft4[1,2]-ft2[1,2]) < dis && ft2[1,3]/ft1[1,3] < bai && abs(ft2[1,2]-ft1[1,2]) < dis){
			ca <- "CE3421"
		} else if(ft4[1,3]/ft2[1,3] < bai && abs(ft4[1,2]-ft2[1,2]) < dis && ft2[1,3]/ft1[1,3] < bai && abs(ft2[1,2]-ft1[1,2]) < dis){
			ca <- "CE421"
		} else if(ft3[1,3]/ft4[1,3] < bai && abs(ft3[1,2]-ft4[1,2]) < dis && ft4[1,3]/ft2[1,3] < bai && abs(ft4[1,2]-ft2[1,2]) < dis){
			ca <- "CE342"
		} else if(ft3[1,3]/ft4[1,3] < bai && abs(ft3[1,2]-ft4[1,2]) < dis && ft2[1,3]/ft1[1,3] < bai && abs(ft2[1,2]-ft1[1,2]) < dis){
			ca <- "CE34_21"
		} else if(ft3[1,3]/ft4[1,3] < bai && abs(ft3[1,2]-ft4[1,2]) < dis){
			ca <- "CE34"
		} else if(ft4[1,3]/ft2[1,3] < bai && abs(ft4[1,2]-ft2[1,2]) < dis){
			ca <- "CE42"
		} else if(ft2[1,3]/ft1[1,3] < bai && abs(ft2[1,2]-ft1[1,2]) < dis){
			ca <- "CE21"
		} else {
			ca <- "CE"
		}
	}

## ft3, ft4, ft1, ft2

	if(ft3[1,1] < ft4[1,1] && ft4[1,1] < ft1[1,1] && ft1[1,1] < ft2[1,1]){
		if(ft3[1,3]/ft4[1,3] < bai && abs(ft3[1,2]-ft4[1,2]) < dis && ft4[1,3]/ft1[1,3] < bai && abs(ft4[1,2]-ft1[1,2]) < dis && ft1[1,3]/ft2[1,3] < bai && abs(ft1[1,2]-ft2[1,2]) < dis){
			ca <- "CF3412"
		} else if(ft4[1,3]/ft1[1,3] < bai && abs(ft4[1,2]-ft1[1,2]) < dis && ft1[1,3]/ft2[1,3] < bai && abs(ft1[1,2]-ft2[1,2]) < dis){
			ca <- "CF412"
		} else if(ft3[1,3]/ft4[1,3] < bai && abs(ft3[1,2]-ft4[1,2]) < dis && ft4[1,3]/ft1[1,3] < bai && abs(ft4[1,2]-ft1[1,2]) < dis){
			ca <- "CF341"
		} else if(ft3[1,3]/ft4[1,3] < bai && abs(ft3[1,2]-ft4[1,2]) < dis && ft1[1,3]/ft2[1,3] < bai && abs(ft1[1,2]-ft2[1,2]) < dis){
			ca <- "CF34_12"
		} else if(ft3[1,3]/ft4[1,3] < bai && abs(ft3[1,2]-ft4[1,2]) < dis){
			ca <- "CF34"
		} else if(ft4[1,3]/ft1[1,3] < bai && abs(ft4[1,2]-ft1[1,2]) < dis){
			ca <- "CF41"
		} else if(ft1[1,3]/ft2[1,3] < bai && abs(ft1[1,2]-ft2[1,2]) < dis){
			ca <- "CF12"
		} else {
			ca <- "CF"
		}
	}

## ft4, ft1, ft2, ft3

	if(ft4[1,1] < ft1[1,1] && ft1[1,1] < ft2[1,1] && ft2[1,1] < ft3[1,1]){
		if(ft4[1,3]/ft1[1,3] < bai && abs(ft4[1,2]-ft1[1,2]) < dis && ft1[1,3]/ft2[1,3] < bai && abs(ft1[1,2]-ft2[1,2]) < dis && ft2[1,3]/ft3[1,3] < bai && abs(ft2[1,2]-ft3[1,2]) < dis){
			ca <- "DA4123"
		} else if(ft1[1,3]/ft2[1,3] < bai && abs(ft1[1,2]-ft2[1,2]) < dis && ft2[1,3]/ft3[1,3] < bai && abs(ft2[1,2]-ft3[1,2]) < dis){
			ca <- "DA123"
		} else if(ft4[1,3]/ft1[1,3] < bai && abs(ft4[1,2]-ft1[1,2]) < dis && ft1[1,3]/ft2[1,3] < bai && abs(ft1[1,2]-ft2[1,2]) < dis){
			ca <- "DA412"
		} else if(ft4[1,3]/ft1[1,3] < bai && abs(ft4[1,2]-ft1[1,2]) < dis && ft2[1,3]/ft3[1,3] < bai && abs(ft2[1,2]-ft3[1,2]) < dis){
			ca <- "DA41_23"
		} else if(ft4[1,3]/ft1[1,3] < bai && abs(ft4[1,2]-ft1[1,2]) < dis){
			ca <- "DA41"
		} else if(ft1[1,3]/ft2[1,3] < bai && abs(ft1[1,2]-ft2[1,2]) < dis){
			ca <- "DA12"
		} else if(ft2[1,3]/ft3[1,3] < bai && abs(ft2[1,2]-ft3[1,2]) < dis){
			ca <- "DA23"
		} else {
			ca <- "DA"
		}
	}

## ft4, ft1, ft3, ft2

	if(ft4[1,1] < ft1[1,1] && ft1[1,1] < ft3[1,1] && ft3[1,1] < ft2[1,1]){
		if(ft4[1,3]/ft1[1,3] < bai && abs(ft4[1,2]-ft1[1,2]) < dis && ft1[1,3]/ft3[1,3] < bai && abs(ft1[1,2]-ft3[1,2]) < dis && ft3[1,3]/ft2[1,3] < bai && abs(ft3[1,2]-ft2[1,2]) < dis){
			ca <- "DB4132"
		} else if(ft1[1,3]/ft3[1,3] < bai && abs(ft1[1,2]-ft3[1,2]) < dis && ft3[1,3]/ft2[1,3] < bai && abs(ft3[1,2]-ft2[1,2]) < dis){
			ca <- "DB132"
		} else if(ft4[1,3]/ft1[1,3] < bai && abs(ft4[1,2]-ft1[1,2]) < dis && ft1[1,3]/ft3[1,3] < bai && abs(ft1[1,2]-ft3[1,2]) < dis){
			ca <- "DB413"
		} else if(ft4[1,3]/ft1[1,3] < bai && abs(ft4[1,2]-ft1[1,2]) < dis && ft3[1,3]/ft2[1,3] < bai && abs(ft3[1,2]-ft2[1,2]) < dis){
			ca <- "DB41_32"
		} else if(ft4[1,3]/ft1[1,3] < bai && abs(ft4[1,2]-ft1[1,2]) < dis){
			ca <- "DB41"
		} else if(ft1[1,3]/ft3[1,3] < bai && abs(ft1[1,2]-ft3[1,2]) < dis){
			ca <- "DB13"
		} else if(ft3[1,3]/ft2[1,3] < bai && abs(ft3[1,2]-ft2[1,2]) < dis){
			ca <- "DB32"
```

```
			} else {
				ca <- "DB"
			}
		}

## ft4, ft2, ft1, ft3

		if(ft4[1,1] < ft2[1,1] && ft2[1,1] < ft1[1,1] && ft1[1,1] < ft3[1,1]){
			if(ft4[1,3]/ft2[1,3] < bai && abs(ft4[1,2]-ft2[1,2]) < dis && ft2[1,3]/ft1[1,3] < bai && abs(ft2[1,2]-ft1[1,2]) < dis && ft1[1,3]/ft3[1,3] < bai && abs(ft1[1,2]-ft3[1,2]) < dis){
				ca <- "DC4213"
			} else if(ft2[1,3]/ft1[1,3] < bai && abs(ft2[1,2]-ft1[1,2]) < dis && ft1[1,3]/ft3[1,3] < bai && abs(ft1[1,2]-ft3[1,2]) < dis){
				ca <- "DC213"
			} else if(ft4[1,3]/ft2[1,3] < bai && abs(ft4[1,2]-ft2[1,2]) < dis && ft2[1,3]/ft1[1,3] < bai && abs(ft2[1,2]-ft1[1,2]) < dis){
				ca <- "DC421"
			} else if(ft4[1,3]/ft2[1,3] < bai && abs(ft4[1,2]-ft2[1,2]) < dis && ft1[1,3]/ft3[1,3] < bai && abs(ft1[1,2]-ft3[1,2]) < dis){
				ca <- "DC42_13"
			} else if(ft4[1,3]/ft2[1,3] < bai && abs(ft4[1,2]-ft2[1,2]) < dis){
				ca <- "DC42"
			} else if(ft2[1,3]/ft1[1,3] < bai && abs(ft2[1,2]-ft1[1,2]) < dis){
				ca <- "DC21"
			} else if(ft1[1,3]/ft3[1,3] < bai && abs(ft1[1,2]-ft3[1,2]) < dis){
				ca <- "DC13"
			} else {
				ca <- "DC"
			}
		}

## ft4, ft2, ft3, ft1

		if(ft4[1,1] < ft2[1,1] && ft2[1,1] < ft3[1,1] && ft3[1,1] < ft1[1,1]){
			if(ft4[1,3]/ft2[1,3] < bai && abs(ft4[1,2]-ft2[1,2]) < dis && ft2[1,3]/ft3[1,3] < bai && abs(ft2[1,2]-ft3[1,2]) < dis && ft3[1,3]/ft1[1,3] < bai && abs(ft3[1,2]-ft1[1,2]) < dis){
				ca <- "DD4231"
			} else if(ft2[1,3]/ft3[1,3] < bai && abs(ft2[1,2]-ft3[1,2]) < dis && ft3[1,3]/ft1[1,3] < bai && abs(ft3[1,2]-ft1[1,2]) < dis){
				ca <- "DD231"
			} else if(ft4[1,3]/ft2[1,3] < bai && abs(ft4[1,2]-ft2[1,2]) < dis && ft2[1,3]/ft3[1,3] < bai && abs(ft2[1,2]-ft3[1,2]) < dis){
				ca <- "DD423"
			} else if(ft4[1,3]/ft2[1,3] < bai && abs(ft4[1,2]-ft2[1,2]) < dis && ft3[1,3]/ft1[1,3] < bai && abs(ft3[1,2]-ft1[1,2]) < dis){
				ca <- "DD42_31"
			} else if(ft4[1,3]/ft2[1,3] < bai && abs(ft4[1,2]-ft2[1,2]) < dis){
				ca <- "DD42"
			} else if(ft2[1,3]/ft3[1,3] < bai && abs(ft2[1,2]-ft3[1,2]) < dis){
				ca <- "DD23"
			} else if(ft3[1,3]/ft1[1,3] < bai && abs(ft3[1,2]-ft1[1,2]) < dis){
				ca <- "DD31"
			} else {
				ca <- "DD"
			}
		}

## ft4, ft3, ft1, ft2

		if(ft4[1,1] < ft3[1,1] && ft3[1,1] < ft1[1,1] && ft1[1,1] < ft2[1,1]){
			if(ft4[1,3]/ft3[1,3] < bai && abs(ft4[1,2]-ft3[1,2]) < dis && ft3[1,3]/ft1[1,3] < bai && abs(ft3[1,2]-ft1[1,2]) < dis && ft1[1,3]/ft2[1,3] < bai && abs(ft1[1,2]-ft2[1,2]) < dis){
				ca <- "DE4312"
			} else if(ft3[1,3]/ft1[1,3] < bai && abs(ft3[1,2]-ft1[1,2]) < dis && ft1[1,3]/ft2[1,3] < bai && abs(ft1[1,2]-ft2[1,2]) < dis){
				ca <- "DE312"
			} else if(ft4[1,3]/ft3[1,3] < bai && abs(ft4[1,2]-ft3[1,2]) < dis && ft3[1,3]/ft1[1,3] < bai && abs(ft3[1,2]-ft1[1,2]) < dis){
				ca <- "DE431"
			} else if(ft4[1,3]/ft3[1,3] < bai && abs(ft4[1,2]-ft3[1,2]) < dis && ft1[1,3]/ft2[1,3] < bai && abs(ft1[1,2]-ft2[1,2]) < dis){
				ca <- "DE43_12"
			} else if(ft4[1,3]/ft3[1,3] < bai && abs(ft4[1,2]-ft3[1,2]) < dis){
				ca <- "DE43"
			} else if(ft3[1,3]/ft1[1,3] < bai && abs(ft3[1,2]-ft1[1,2]) < dis){
				ca <- "DE31"
			} else if(ft1[1,3]/ft2[1,3] < bai && abs(ft1[1,2]-ft2[1,2]) < dis){
				ca <- "DE12"
			} else {
				ca <- "DE"
			}
		}

## ft4, ft3, ft2, ft1

		if(ft4[1,1] < ft3[1,1] && ft3[1,1] < ft2[1,1] && ft2[1,1] < ft1[1,1]){
			if(ft4[1,3]/ft3[1,3] < bai && abs(ft4[1,2]-ft3[1,2]) < dis && ft3[1,3]/ft2[1,3] < bai && abs(ft3[1,2]-ft2[1,2]) < dis && ft2[1,3]/ft1[1,3] < bai && abs(ft2[1,2]-ft1[1,2]) < dis){
				ca <- "DF4321"
			} else if(ft3[1,3]/ft2[1,3] < bai && abs(ft3[1,2]-ft2[1,2]) < dis && ft2[1,3]/ft1[1,3] < bai && abs(ft2[1,2]-ft1[1,2]) < dis){
				ca <- "DF321"
			} else if(ft4[1,3]/ft3[1,3] < bai && abs(ft4[1,2]-ft3[1,2]) < dis && ft3[1,3]/ft2[1,3] < bai && abs(ft3[1,2]-ft2[1,2]) < dis){
				ca <- "DF432"
			} else if(ft4[1,3]/ft3[1,3] < bai && abs(ft4[1,2]-ft3[1,2]) < dis && ft2[1,3]/ft1[1,3] < bai && abs(ft2[1,2]-ft1[1,2]) < dis){
				ca <- "DF43_21"
			} else if(ft4[1,3]/ft3[1,3] < bai && abs(ft4[1,2]-ft3[1,2]) < dis){
```

```
				ca <- "DF43"
			} else if(ft3[1,3]/ft2[1,3] < bai && abs(ft3[1,2]-ft2[1,2]) < dis){
				ca <- "DF32"
			} else if(ft2[1,3]/ft1[1,3] < bai && abs(ft2[1,2]-ft1[1,2]) < dis){
				ca <- "DF21"
			} else {
				ca <- "DF"
			}
		}

#### extract a separation point

		if(ca == "AA"){

			tminmaxt <- minmaxt[(ft1[1,1]+1):(ft4[1,1]-1), ,drop=F]
			tminmaxt <- tminmaxt[tminmaxt[,4] == 0, ,drop=F]
			tminmaxt <- tminmaxt[order(tminmaxt[,1], decreasing=T),,drop=F]
			lt <- nrow(tminmaxt)
			if(lt == 1){
				tht <- tminmaxt[1, ,drop=F]
			} else if(lt == 2){
				if(tminmaxt[2,3]/tminmaxt[1,3] < 1.5 && tminmaxt[2,1] > tminmaxt[1,1]){
					tht <- tminmaxt[2, ,drop=F]
				} else {
					tht <- tminmaxt[1, ,drop=F]
				}
			} else if(lt >= 3){
				sl <- cbind(1:lt)
				wa <-c()
				for( s in 1:lt){
					if(s == 1){
						sp <- 0
					} else {
						sp <- (tminmaxt[s - 1,3] - tminmaxt[s,3])/(tminmaxt[s - 1,2] - tminmaxt[s,2])
					}
					wa <- rbind(wa,sp)
				}
				sl <- cbind(sl,wa)
				sl <- sl[order(sl[,2], decreasing=T), ,drop=F]
				if(sl[1,2] == 0){
					if(tminmaxt[sl[2,1],2] > 10){
						tht <- tminmaxt[sl[3,1] , ,drop=F]
					} else {
						tht <- tminmaxt[sl[2,1] , ,drop=F]
					}
				} else {
					tht <- tminmaxt[sl[1,1] , ,drop=F]
				}
			}
		}

		if(ca == "AA12" ){

			if (ft3[1,3]/ft4[1,3] < 1.6){
				tminmaxt <- minmaxt[(ft2[1,1]+1):(ft3[1,1]-1), ,drop=F]
				tminmaxt <- tminmaxt[order(tminmaxt[,3], decreasing=F),,drop=F]
				tht <- tminmaxt[1, ,drop=F]
			} else {
				tminmaxt <- minmaxt[(ft2[1,1]+1):(ft4[1,1]-1), ,drop=F]
				tminmaxt <- tminmaxt[order(tminmaxt[,3], decreasing=F),,drop=F]
				tht <- tminmaxt[1, ,drop=F]
			}
		}

		if(ca == "AA12_34" ){

			tminmaxt <- minmaxt[(ft1[1,1]+1):(ft4[1,1]-1), ,drop=F]
			tminmaxt <- tminmaxt[tminmaxt[,4] == 0, ,drop=F]
			tminmaxt <- tminmaxt[order(tminmaxt[,1], decreasing=T),,drop=F]
			lt <- nrow(tminmaxt)
			if(lt == 1){
				tht <- tminmaxt[1, ,drop=F]
			} else if(lt == 2){
				if(tminmaxt[2,3]/tminmaxt[1,3] < 1.5 && tminmaxt[2,1] > tminmaxt[1,1]){
					tht <- tminmaxt[2, ,drop=F]
				} else {
					tht <- tminmaxt[1, ,drop=F]
				}
			} else if(lt >= 3){
				sl <- cbind(1:lt)
				wa <-c()
				for( s in 1:lt){
					if(s == 1){
						sp <- 0
					} else {
						sp <- (tminmaxt[s - 1,3] - tminmaxt[s,3])/(tminmaxt[s - 1,2] - tminmaxt[s,2])
					}
```

```
								wa <- rbind(wa,sp)
						}
						sl <- cbind(sl,wa)
						sl <- sl[order(sl[,2], decreasing=T), ,drop=F]
						if(sl[1,2] == 0){
								if(tminmaxt[sl[2,1],2] > 10){
										tht <- tminmaxt[sl[3,1] , ,drop=F]
								} else {
										tht <- tminmaxt[sl[2,1] , ,drop=F]
								}
						} else {
								tht <- tminmaxt[sl[1,1] , ,drop=F]
						}
						if((minmaxt[tminmaxt[sl[1,1],1]+2,3]  -  minmaxt[tminmaxt[sl[1,1],1]+1,3])/(minmaxt[tminmaxt[sl[1,1],1]+2,2]  -
minmaxt[tminmaxt[sl[1,1],1]+1,2]) > -0.0001){
								tht <- tminmaxt[sl[2,1] , ,drop=F]
						}
				}
		}

		if(ca == "AA23"){

				if(ft4[1,2] > 5){
						tminmaxt <- minmaxt[(ft1[1,1]+1):(ft3[1,1]-1), ,drop=F]
						tminmaxt <- tminmaxt[order(tminmaxt[,3], decreasing=F),,drop=F]
						tht <- tminmaxt[1, ,drop=F]
				} else {
						tminmaxt <- minmaxt[(ft1[1,1]+1):(ft4[1,1]-1), ,drop=F]
						tminmaxt <- tminmaxt[order(tminmaxt[,3], decreasing=F),,drop=F]
						tht <- tminmaxt[1, ,drop=F]
				}
		}

		if(ca == "AA34" ){

				if(ft2[1,3]/ft3[1,3] < 1.1){
						tminmaxt <- minmaxt[(ft1[1,1]+1):(ft2[1,1]-1), ,drop=F]
						tminmaxt <- tminmaxt[order(tminmaxt[,3], decreasing=F),,drop=F]
						tht <- tminmaxt[1, ,drop=F]
				} else {
						tminmaxt <- minmaxt[(ft1[1,1]+1):(ft4[1,1]-1), ,drop=F]
						tminmaxt <- tminmaxt[order(tminmaxt[,3], decreasing=F),,drop=F]
						tht <- tminmaxt[1, ,drop=F]
				}
		}

		if(ca == "AA234"){

				tminmaxt <- minmaxt[(ft1[1,1]):(ft4[1,1]), ,drop=F]
				tminmaxt <- tminmaxt[tminmaxt[,4] == 0, ,drop=F]
				tminmaxt <- tminmaxt[order(tminmaxt[,1], decreasing=T),,drop=F]
				lt <- nrow(tminmaxt)
				if(lt == 1){
						tht <- tminmaxt[1, ,drop=F]
				} else if(lt == 2){
						if(tminmaxt[2,3]/tminmaxt[1,3] < 1.5 && tminmaxt[2,1] > tminmaxt[1,1]){
								tht <- tminmaxt[2, ,drop=F]
						} else {
								tht <- tminmaxt[1, ,drop=F]
						}
				} else if(lt >= 3){
						tminmaxt <- tminmaxt[order(tminmaxt[,1], decreasing=T),,drop=F]
						sl <- cbind(1:lt)
						wa <-c()
						for( s in 1:lt){
								if(s == 1){
										sp <- 0
								} else {
										sp <- (tminmaxt[s - 1,3] - tminmaxt[s,3])/(tminmaxt[s - 1,2] - tminmaxt[s,2])
								}
								wa <- rbind(wa,sp)
						}
						sl <- cbind(sl,wa)
						sl <- sl[order(sl[,2], decreasing=T), ,drop=F]
						if(sl[1,2] == 0){
								tht <- tminmaxt[sl[2,1] , ,drop=F]
						} else {
								tht <- tminmaxt[sl[1,1] , ,drop=F]
						}
						tminmaxt <- tminmaxt[order(tminmaxt[,3], decreasing=F),,drop=F]
						if(ft1[1,2]  <  tminmaxt[1,2]  &&  tminmaxt[1,2]  <  ft2[1,2]  &&  (tminmaxt[2,3]  -  tminmaxt[1,3])/(tminmaxt[2,2]-
tminmaxt[1,2]) < 0.00001){
								tht <- tminmaxt[1, ,drop=F]
						}
				}
		}
```

```
if(ca == "AB12"){

        tminmaxt <- minmaxt[(ft1[1,1]+1):(ft3[1,1]-1), ,drop=F]
        tminmaxt <- tminmaxt[order(tminmaxt[,3], decreasing=F),,drop=F]
        tht <- tminmaxt[1, ,drop=F]
}

if(ca == "AB24"){

        tminmaxt <- minmaxt[(ft1[1,1]+1):(ft3[1,1]-1), ,drop=F]
        tminmaxt <- tminmaxt[order(tminmaxt[,3], decreasing=F),,drop=F]
        tht <- tminmaxt[1, ,drop=F]
}

if(ca == "AB243"){

        tminmaxt <- minmaxt[(ft1[1,1]+1):(ft2[1,1]-1), ,drop=F]
        tminmaxt <- tminmaxt[order(tminmaxt[,3], decreasing=F),,drop=F]
        tht <- tminmaxt[1, ,drop=F]
}

if(ca == "AB43"){

        tminmaxt <- minmaxt[(ft1[1,1]):(ft4[1,1]), ,drop=F]
        tminmaxt <- tminmaxt[tminmaxt[,4] == 0, ,drop=F]
        tminmaxt <- tminmaxt[order(tminmaxt[,1], decreasing=T),,drop=F]
        lt <- nrow(tminmaxt)
        if(lt == 1){
                tht <- tminmaxt[1, ,drop=F]
        } else if(lt == 2){
                if((ft2[1,3] - minmaxt[ft2[1,1]-2, 3])/(ft2[1,2] - minmaxt[ft2[1,1]-2, 2]) < 0.0007 || (ft2[1,3] - minmaxt[ft2[1,1]-2,
3])/(ft2[1,2] - minmaxt[ft2[1,1]-2, 2]) > 0.0009){
                        tht <- tminmaxt[2, ,drop=F]
                } else {
                        tht <- tminmaxt[1, ,drop=F]
                }
        } else if(lt >= 3){
                sl <- cbind(1:lt)
                wa <-c()
                for( s in 1:lt){
                        if(s == 1){
                                sp <- 0
                        } else {
                                sp <- (tminmaxt[s - 1,3] - tminmaxt[s,3])/(tminmaxt[s - 1,2] - tminmaxt[s,2])
                        }
                        wa <- rbind(wa,sp)
                }
                sl <- cbind(sl,wa)
                sl <- sl[order(sl[,2], decreasing=T), ,drop=F]
                if(sl[1,2] == 0){
                        tht <- tminmaxt[sl[2,1] , ,drop=F]
                } else {
                        tht <- tminmaxt[sl[1,1] , ,drop=F]
                }
                if(sl[1,2] == 0 && sl[2,2] <0 ){
                        tht <- tminmaxt[sl[1,1] , ,drop=F]
                }
        }
}

if(ca == "AB12_43"){

        tminmaxt <- minmaxt[(ft1[1,1]+1):(ft3[1,1]-1), ,drop=F]
        tminmaxt <- tminmaxt[order(tminmaxt[,3], decreasing=F),,drop=F]
        tht <- tminmaxt[1, ,drop=F]
}

if(ca == "AB"){

        tminmaxt <- minmaxt[(ft1[1,1]+1):(ft3[1,1]-1), ,drop=F]
        tminmaxt <- tminmaxt[tminmaxt[,4] == 0, ,drop=F]
        tminmaxt <- tminmaxt[order(tminmaxt[,1], decreasing=T),,drop=F]
        lt <- nrow(tminmaxt)
        if(lt == 1){
                tht <- tminmaxt[1, ,drop=F]
        } else if(lt == 2){
                if((ft2[1,3] - minmaxt[ft2[1,1]-2, 3])/(ft2[1,2] - minmaxt[ft2[1,1]-2, 2]) < 0.0007 || (ft2[1,3] - minmaxt[ft2[1,1]-2,
3])/(ft2[1,2] - minmaxt[ft2[1,1]-2, 2]) > 0.0009){
                        tht <- tminmaxt[2, ,drop=F]
                } else {
                        tht <- tminmaxt[1, ,drop=F]
                }
        } else if(lt >= 3){
                sl <- cbind(1:lt)
                wa <-c()
                for( s in 1:lt){
                        if(s == 1){
```

```
						sp <- 0
					} else {
						sp <- (tminmaxt[s - 1,3] - tminmaxt[s,3])/(tminmaxt[s - 1,2] - tminmaxt[s,2])
					}
					wa <- rbind(wa,sp)
				}
				sl <- cbind(sl,wa)
				sl <- sl[order(sl[,2], decreasing=T), ,drop=F]
				if(sl[1,2] == 0){
					tht <- tminmaxt[1 , ,drop=F]
				} else {
					if(minmaxt[tminmaxt[sl[1,1],1] + 1,3]/tminmaxt[sl[1,1],3] > 1.1){
						tht <- tminmaxt[sl[1,1] , ,drop=F]
					} else {
						tht <- minmaxt[tminmaxt[sl[1,1], 1] + 2 , ,drop=F]
					}
				}
			}
		}

		if(ca == "AC"){

			tminmaxt <- minmaxt[(ft1[1,1]+1):(ft2[1,1]-1), ,drop=F]
			tminmaxt <- tminmaxt[tminmaxt[,4] == 0, ,drop=F]
			tminmaxt <- tminmaxt[order(tminmaxt[,1], decreasing=T),,drop=F]
			lt <- nrow(tminmaxt)
			if(lt == 1){
				tht <- tminmaxt[1, ,drop=F]
			} else if(lt == 2){
				if((ft2[1,3] - minmaxt[ft2[1,1]-2, 3])/(ft2[1,2] - minmaxt[ft2[1,1]-2, 2]) < 0.0007 || (ft2[1,3] - minmaxt[ft2[1,1]-2,
3])/(ft2[1,2] - minmaxt[ft2[1,1]-2, 2]) > 0.0009){
					if(minmaxt[ft2[1,1]-2,3]/minmaxt[ft2[1,1]-1,3] < 1.1){
						tht <- tminmaxt[1, ,drop=F]
					} else {
						tht <- tminmaxt[2, ,drop=F]
					}
				} else {
					tht <- tminmaxt[1, ,drop=F]
				}
			} else if(lt >= 3){
				sl <- cbind(1:lt)
				wa <-c()
				for( s in 1:lt){
					if(s == 1){
						sp <- 0
					} else {
						sp <- (tminmaxt[s - 1,3] - tminmaxt[s,3])/(tminmaxt[s - 1,2] - tminmaxt[s,2])
					}
					wa <- rbind(wa,sp)
				}
				sl <- cbind(sl,wa)
				sl <- sl[order(sl[,2], decreasing=T), ,drop=F]
				if(sl[1,2] == 0){
					tht <- tminmaxt[sl[2,1] , ,drop=F]
				} else {
					tht <- tminmaxt[sl[1,1] , ,drop=F]
				}
			}
		}

		if(ca == "AC12"){

			tminmaxt <- minmaxt[(ft1[1,1]+1):(ft2[1,1]-1), ,drop=F]
			tminmaxt <- tminmaxt[order(tminmaxt[,3], decreasing=F),,drop=F]
			tht <- tminmaxt[1, ,drop=F]
		}

		if(ca == "AC24"){

			tminmaxt <- minmaxt[(ft1[1,1]+1):(ft2[1,1]-1), ,drop=F]
			tminmaxt <- tminmaxt[order(tminmaxt[,3], decreasing=F),,drop=F]
			tht <- tminmaxt[1, ,drop=F]
		}

		if(ca == "AC32"){

			tminmaxt <- minmaxt[(ft1[1,1]+1):(ft2[1,1]-1), ,drop=F]
			tminmaxt <- tminmaxt[tminmaxt[,4] == 0, ,drop=F]
			tminmaxt <- tminmaxt[order(tminmaxt[,1], decreasing=T),,drop=F]
			lt <- nrow(tminmaxt)
			if(lt == 1){
				tht <- tminmaxt[1, ,drop=F]
			} else if(lt == 2){
				if((ft2[1,3] - minmaxt[ft2[1,1]-2, 3])/(ft2[1,2] - minmaxt[ft2[1,1]-2, 2]) < 0.0007 || (ft2[1,3] - minmaxt[ft2[1,1]-2,
3])/(ft2[1,2] - minmaxt[ft2[1,1]-2, 2]) > 0.0009){
					tht <- tminmaxt[2, ,drop=F]
				} else {
```

```
                        tht <- tminmaxt[1, ,drop=F]
                }
        } else if(lt >= 3){
                sl <- cbind(1:lt)
                wa <-c()
                for( s in 1:lt){
                        if(s == 1){
                                sp <- 0
                        } else {
                                sp <- (tminmaxt[s - 1,3] - tminmaxt[s,3])/(tminmaxt[s - 1,2] - tminmaxt[s,2])
                        }
                        wa <- rbind(wa,sp)
                }
                sl <- cbind(sl,wa)
                sl <- sl[order(sl[,2], decreasing=T), ,drop=F]
                if(sl[1,2] == 0){
                        tht <- tminmaxt[sl[2,1] , ,drop=F]
                } else {
                        tht <- tminmaxt[sl[1,1] , ,drop=F]
                }
        }
}

if(ca == "AC324"){

        tminmaxt <- minmaxt[(ft1[1,1]+1):(ft2[1,1]-1), ,drop=F]
        tminmaxt <- tminmaxt[order(tminmaxt[,3], decreasing=F),,drop=F]
        tht <- tminmaxt[1, ,drop=F]
}

if(ca == "AD"){

        tminmaxt <- minmaxt[(ft1[1,1]+1):(ft2[1,1]-1), ,drop=F]
        tminmaxt <- tminmaxt[tminmaxt[,4] == 0, ,drop=F]
        tminmaxt <- tminmaxt[order(tminmaxt[,1], decreasing=T),,drop=F]
        lt <- nrow(tminmaxt)
        if(lt == 1){
                tht <- tminmaxt[1, ,drop=F]
        } else if(lt == 2){
                if((ft2[1,3] - minmaxt[ft2[1,1]-2, 3])/(ft2[1,2] - minmaxt[ft2[1,1]-2, 2]) < 0.0007 || (ft2[1,3] - minmaxt[ft2[1,1]-2,
3])/(ft2[1,2] - minmaxt[ft2[1,1]-2, 2]) > 0.0009){
                        tht <- tminmaxt[2, ,drop=F]
                } else {
                        tht <- tminmaxt[1, ,drop=F]
                }
        } else if(lt >= 3){
                sl <- cbind(1:lt)
                wa <-c()
                for( s in 1:lt){
                        if(s == 1){
                                sp <- 0
                        } else {
                                sp <- (tminmaxt[s - 1,3] - tminmaxt[s,3])/(tminmaxt[s - 1,2] - tminmaxt[s,2])
                        }
                        wa <- rbind(wa,sp)
                }
                sl <- cbind(sl,wa)
                sl <- sl[order(sl[,2], decreasing=T), ,drop=F]
                if(sl[1,2] == 0){
                        tht <- tminmaxt[sl[2,1] , ,drop=F]
                } else {
                        tht <- tminmaxt[sl[1,1] , ,drop=F]
                }
                if(minmaxt[ft2[1,1]-2,3]/minmaxt[ft2[1,1]-1,3] < 1.01){
                        tht <- minmaxt[ft2[1,1]-1, ,drop=F]
                }
        }
}

if(ca == "AD342"){

        if ( ft3[1,3]/ft4[1,3] < 1.1){
                tminmaxt <- minmaxt[(ft1[1,1]+1):(ft3[1,1]-1), ,drop=F]
                tminmaxt <- tminmaxt[order(tminmaxt[,3], decreasing=F),,drop=F]
                tht <- tminmaxt[1, ,drop=F]
        } else {
                tminmaxt <- minmaxt[(ft1[1,1]+1):(ft2[1,1]-1), ,drop=F]
                tminmaxt <- tminmaxt[order(tminmaxt[,3], decreasing=F),,drop=F]
                tht <- tminmaxt[1, ,drop=F]
        }
}

if(ca == "AD34"){

        tminmaxt <- minmaxt[(ft1[1,1]+1):(ft2[1,1]-1), ,drop=F]
        tminmaxt <- tminmaxt[order(tminmaxt[,3], decreasing=F),,drop=F]
        tht <- tminmaxt[1, ,drop=F]
```

```
}

if(ca == "AE423"){

tminmaxt <- minmaxt[(ft1[1,1]+1):(ft2[1,1]-1), ,drop=F]
tminmaxt <- tminmaxt[tminmaxt[,4] == 0, ,drop=F]
tminmaxt <- tminmaxt[order(tminmaxt[,1], decreasing=T),,drop=F]
lt <- nrow(tminmaxt)
if(lt == 1){
tht <- tminmaxt[1, ,drop=F]
} else if(lt == 2){
if((ft2[1,3] - minmaxt[ft2[1,1]-2, 3])/(ft2[1,2] - minmaxt[ft2[1,1]-2, 2]) < 0.0007 || (ft2[1,3] - minmaxt[ft2[1,1]-2, 3])/(ft2[1,2] - minmaxt[ft2[1,1]-2, 2]) > 0.0009){
if (abs(tminmaxt[1,3]-minmaxt[tminmaxt[2,1]+1,3])/(tminmaxt[1,2]-minmaxt[tminmaxt[2,1]+1,2]) < 2e-5){
tht <- tminmaxt[1, ,drop=F]
} else {
tht <- tminmaxt[2, ,drop=F]
}
} else {
tht <- tminmaxt[1, ,drop=F]
}
} else if(lt >= 3){
sl <- cbind(1:lt)
wa <-c()
for( s in 1:lt){
if(s == 1){
sp <- 0
} else {
sp <- (tminmaxt[s - 1,3] - tminmaxt[s,3])/(tminmaxt[s - 1,2] - tminmaxt[s,2])
}
wa <- rbind(wa,sp)
}
sl <- cbind(sl,wa)
sl <- sl[order(sl[,2], decreasing=T), ,drop=F]
if(sl[1,2] == 0){
tht <- tminmaxt[sl[2,1] , ,drop=F]
} else {
tht <- tminmaxt[sl[1,1] , ,drop=F]
}
if((minmaxt[tminmaxt[sl[1,1],1]+2,3] - minmaxt[tminmaxt[sl[1,1],1]+1,3])/(minmaxt[tminmaxt[sl[1,1],1]+2,2] - minmaxt[tminmaxt[sl[1,1],1]+1,2]) > -0.0001){
tminmaxt <- tminmaxt[order(tminmaxt[,3], decreasing=F),,drop=F]
tht <- tminmaxt[1 , ,drop=F]
}
}
}

if(ca == "AE42"){

tminmaxt <- minmaxt[(ft1[1,1]+1):(ft3[1,1]-1), ,drop=F]
tminmaxt <- tminmaxt[tminmaxt[,4] == 0, ,drop=F]
tminmaxt <- tminmaxt[order(tminmaxt[,1], decreasing=T),,drop=F]
lt <- nrow(tminmaxt)
if(lt == 1){
tht <- tminmaxt[1, ,drop=F]
} else if(lt == 2){
if((ft2[1,3] - minmaxt[ft2[1,1]-2, 3])/(ft2[1,2] - minmaxt[ft2[1,1]-2, 2]) < 0.0007 || (ft2[1,3] - minmaxt[ft2[1,1]-2, 3])/(ft2[1,2] - minmaxt[ft2[1,1]-2, 2]) > 0.0009){
tht <- tminmaxt[2, ,drop=F]
} else {
tht <- tminmaxt[1, ,drop=F]
}
} else if(lt >= 3){
sl <- cbind(1:lt)
wa <-c()
for( s in 1:lt){
if(s == 1){
sp <- 0
} else {
sp <- (tminmaxt[s - 1,3] - tminmaxt[s,3])/(tminmaxt[s - 1,2] - tminmaxt[s,2])
}
wa <- rbind(wa,sp)
}
sl <- cbind(sl,wa)
sl <- sl[order(sl[,2], decreasing=T), ,drop=F]
if(sl[1,2] == 0){
tht <- tminmaxt[sl[2,1] , ,drop=F]
} else {
tht <- tminmaxt[sl[1,1] , ,drop=F]
}
if((tminmaxt[sl[1,1]-1,3] - tminmaxt[sl[1,1],3])/(tminmaxt[sl[1,1]-1,2] - tminmaxt[sl[1,1],2]) < 0.0003){
if(tminmaxt[sl[1,1],2] < tminmaxt[sl[1,1]-1,2] && tminmaxt[sl[1,1]-1,3]/tminmaxt[sl[1,1],3] < 1.25){
tht <- tminmaxt[sl[1,1]-1, ,drop=F]
} else {
tht <- tminmaxt[sl[1,1], ,drop=F]
```

```
							}
						}
					}
				}

				if(ca == "AE23"){

					tminmaxt <- minmaxt[(ft1[1,1]+1):(ft3[1,1]-1), ,drop=F]
					tminmaxt <- tminmaxt[tminmaxt[,4] == 0, ,drop=F]
					tminmaxt <- tminmaxt[order(tminmaxt[,1], decreasing=T),,drop=F]
					lt <- nrow(tminmaxt)
					if(lt == 1){
						tht <- tminmaxt[1, ,drop=F]
					} else if(lt == 2){
						if((ft2[1,3] - minmaxt[ft2[1,1]-2, 3])/(ft2[1,2] - minmaxt[ft2[1,1]-2, 2]) < 0.0007 || (ft2[1,3] - minmaxt[ft2[1,1]-2,
3])/(ft2[1,2] - minmaxt[ft2[1,1]-2, 2]) > 0.0009){
							tht <- tminmaxt[2, ,drop=F]
						} else {
							tht <- tminmaxt[1, ,drop=F]
						}
					} else if(lt >= 3){
						sl <- cbind(1:lt)
						wa <-c()
						for( s in 1:lt){
							if(s == 1){
								sp <- 0
							} else {
								sp <- (tminmaxt[s - 1,3] - tminmaxt[s,3])/(tminmaxt[s - 1,2] - tminmaxt[s,2])
							}
							wa <- rbind(wa,sp)
						}
						sl <- cbind(sl,wa)
						sl <- sl[order(sl[,2], decreasing=T), ,drop=F]
						if(sl[1,2] == 0){
							tht <- tminmaxt[sl[2,1] , ,drop=F]
						} else {
							tht <- tminmaxt[sl[1,1] , ,drop=F]
						}
					}
				}

				if(ca == "AE"){

					tminmaxt <- minmaxt[(ft1[1,1]+1):(ft3[1,1]-1), ,drop=F]
					tminmaxt <- tminmaxt[order(tminmaxt[,3], decreasing=F),,drop=F]
					if(tminmaxt[2,3]/tminmaxt[1,3] < 1.25 && tminmaxt[1,1] < tminmaxt[2,1]){
						tht <- tminmaxt[2, ,drop=F]
					} else {
						tht <- tminmaxt[1, ,drop=F]
					}
				}

				if(ca == "AF32"){

					tminmaxt <- minmaxt[(ft1[1,1]+1):(ft2[1,1]-1), ,drop=F]
					tminmaxt <- tminmaxt[order(tminmaxt[,3], decreasing=F),,drop=F]
					if(tminmaxt[2,3]/tminmaxt[1,3] < 1.25 && tminmaxt[1,1] < tminmaxt[2,1]){
						tht <- tminmaxt[2, ,drop=F]
					} else {
						tht <- tminmaxt[1, ,drop=F]
					}
				}

				if(ca == "AF43"){

					tminmaxt <- minmaxt[(ft1[1,1]+1):(ft2[1,1]-1), ,drop=F]
					tminmaxt <- tminmaxt[tminmaxt[,4] == 0, ,drop=F]
					tminmaxt <- tminmaxt[order(tminmaxt[,1], decreasing=T),,drop=F]
					lt <- nrow(tminmaxt)
					if(lt == 1){
						tht <- tminmaxt[1, ,drop=F]
					} else if(lt == 2){
						if((ft2[1,3] - minmaxt[ft2[1,1]-2, 3])/(ft2[1,2] - minmaxt[ft2[1,1]-2, 2]) < 0.0007 || (ft2[1,3] - minmaxt[ft2[1,1]-2,
3])/(ft2[1,2] - minmaxt[ft2[1,1]-2, 2]) > 0.0009){
							tht <- tminmaxt[2, ,drop=F]
						} else {
							tht <- tminmaxt[1, ,drop=F]
						}
					} else if(lt >= 3){
						sl <- cbind(1:lt)
						wa <-c()
						for( s in 1:lt){
							if(s == 1){
								sp <- 0
							} else {
								sp <- (tminmaxt[s - 1,3] - tminmaxt[s,3])/(tminmaxt[s - 1,2] - tminmaxt[s,2])
							}
```

```
					wa <- rbind(wa,sp)
				}
				sl <- cbind(sl,wa)
				sl <- sl[order(sl[,2], decreasing=T), ,drop=F]
				if(sl[1,2] == 0){
					tht <- tminmaxt[sl[2,1] , ,drop=F]
				} else {
					tht <- tminmaxt[sl[1,1] , ,drop=F]
				}
			}
		}

		if(ca == "AF432"){

			tminmaxt <- minmaxt[(ft1[1,1]):(ft4[1,1]), ,drop=F]
			tminmaxt <- tminmaxt[tminmaxt[,4] == 0, ,drop=F]
			tminmaxt <- tminmaxt[order(tminmaxt[,1], decreasing=T),,drop=F]
			lt <- nrow(tminmaxt)
			if(lt == 1){
				tht <- tminmaxt[1, ,drop=F]
			} else if(lt == 2){
				if((ft2[1,3] - minmaxt[ft2[1,1]-2, 3])/(ft2[1,2] - minmaxt[ft2[1,1]-2, 2]) < 0.0007 || (ft2[1,3] - minmaxt[ft2[1,1]-2,
3])/(ft2[1,2] - minmaxt[ft2[1,1]-2, 2]) > 0.0009){
					tht <- tminmaxt[2, ,drop=F]
				} else {
					tht <- tminmaxt[1, ,drop=F]
				}
			} else if(lt >= 3){
				sl <- cbind(1:lt)
				wa <-c()
				for( s in 1:lt){
					if(s == 1){
						sp <- 0
					} else {
						sp <- (tminmaxt[s - 1,3] - tminmaxt[s,3])/(tminmaxt[s - 1,2] - tminmaxt[s,2])
					}
					wa <- rbind(wa,sp)
				}
				sl <- cbind(sl,wa)
				sl <- sl[order(sl[,2], decreasing=T), ,drop=F]
				if(sl[1,2] == 0){
					tht <- tminmaxt[sl[2,1] , ,drop=F]
				} else {
					tht <- tminmaxt[sl[1,1] , ,drop=F]
				}
			}
		}

		if(ca == "AF"){

			tminmaxt <- minmaxt[(ft1[1,1]+1):(ft2[1,1]-1), ,drop=F]
			tminmaxt <- tminmaxt[tminmaxt[,4] == 0, ,drop=F]
			tminmaxt <- tminmaxt[order(tminmaxt[,1], decreasing=T),,drop=F]
			lt <- nrow(tminmaxt)
			if(lt == 1){
				tht <- tminmaxt[1, ,drop=F]
			} else if(lt == 2){
				if((ft2[1,3] - minmaxt[ft2[1,1]-2, 3])/(ft2[1,2] - minmaxt[ft2[1,1]-2, 2]) < 0.0007 || (ft2[1,3] - minmaxt[ft2[1,1]-2,
3])/(ft2[1,2] - minmaxt[ft2[1,1]-2, 2]) > 0.0009){
					if((tminmaxt[1,3] - tminmaxt[2,3])/(tminmaxt[1,2] - tminmaxt[2,2]) > 0){
						tht <- tminmaxt[2, ,drop=F]
					} else {
						tht <- tminmaxt[1, ,drop=F]
					}
				} else {
					tht <- tminmaxt[1, ,drop=F]
				}
			} else if(lt >= 3){
				sl <- cbind(1:lt)
				wa <-c()
				for( s in 1:lt){
					if(s == 1){
						sp <- 0
					} else {
						sp <- (tminmaxt[s - 1,3] - tminmaxt[s,3])/(tminmaxt[s - 1,2] - tminmaxt[s,2])
					}
					wa <- rbind(wa,sp)
				}
				sl <- cbind(sl,wa)
				sl <- sl[order(sl[,2], decreasing=T), ,drop=F]
				if(sl[1,2] == 0){
					tht <- tminmaxt[sl[2,1] , ,drop=F]
				} else {
					tht <- tminmaxt[sl[1,1] , ,drop=F]
				}
				if(tminmaxt[sl[1,1],3]/tminmaxt[sl[2,1],3] < 1.1 ){
					tht <- tminmaxt[sl[2,1], ,drop=F]
```

```
				}
			}
		}

		if(ca == "BA21"){

			tminmaxt <- minmaxt[(ft1[1,1]+1):(ft4[1,1]-1), ,drop=F]
			tminmaxt <- tminmaxt[order(tminmaxt[,3], decreasing=F),,drop=F]
				tminmaxt <- tminmaxt[tminmaxt[,4] == 0, ,drop=F]
				tminmaxt <- tminmaxt[order(tminmaxt[,2], decreasing=F),,drop=F]
				if(tminmaxt[1,3]/tminmaxt[2,3] > 3 ){
					tht <- tminmaxt[2, ,drop=F]
				} else {
					tht <- tminmaxt[1, ,drop=F]
				}
		}

		if(ca == "BA21_34"){

			tminmaxt <- minmaxt[(ft2[1,1]+1):(ft4[1,1]-1), ,drop=F]
			tminmaxt <- tminmaxt[tminmaxt[,4] == 0, ,drop=F]
			tminmaxt <- tminmaxt[order(tminmaxt[,1], decreasing=T),,drop=F]
			lt <- nrow(tminmaxt)
			if(lt == 1){
				tht <- tminmaxt[1, ,drop=F]
			} else if(lt == 2){
				if((ft2[1,3] - minmaxt[ft2[1,1]-2, 3])/(ft2[1,2] - minmaxt[ft2[1,1]-2, 2]) < 0.0007 || (ft2[1,3] - minmaxt[ft2[1,1]-2,
3])/(ft2[1,2] - minmaxt[ft2[1,1]-2, 2]) > 0.0009){
					if((tminmaxt[1,3] - tminmaxt[2,3])/(tminmaxt[1,2] - tminmaxt[2,2]) > 0){
						tht <- tminmaxt[2, ,drop=F]
					} else {
						tht <- tminmaxt[1, ,drop=F]
					}
				} else {
					tht <- tminmaxt[1, ,drop=F]
				}
			} else if(lt >= 3){
				sl <- cbind(1:lt)
				wa <-c()
				for( s in 1:lt){
					if(s == 1){
						sp <- 0
					} else {
						sp <- (tminmaxt[s - 1,3] - tminmaxt[s,3])/(tminmaxt[s - 1,2] - tminmaxt[s,2])
					}
					wa <- rbind(wa,sp)
				}
				sl <- cbind(sl,wa)
				sl <- sl[order(sl[,2], decreasing=T), ,drop=F]
				if(sl[1,2] == 0){
					tht <- tminmaxt[sl[2,1] , ,drop=F]
				} else {
					tht <- tminmaxt[sl[1,1] , ,drop=F]
				}
				tminmaxt <- tminmaxt[order(tminmaxt[,3], decreasing=F),,drop=F]
				if(tht[1,3] > tminmaxt[1,3] && tht[1,2] > tminmaxt[1,2]){
					tht <- tminmaxt[1, ,drop=F]
				}
			}
		}

		if(ca == "BA34"){

			tminmaxt <- minmaxt[(ft2[1,1]+1):(ft3[1,1]-1), ,drop=F]
			tminmaxt <- tminmaxt[order(tminmaxt[,3], decreasing=F),,drop=F]
			tht <- tminmaxt[1, ,drop=F]
		}

		if(ca == "BA213"){

			tminmaxt <- minmaxt[(ft3[1,1]+1):(ft4[1,1]-1), ,drop=F]
			tminmaxt <- tminmaxt[order(tminmaxt[,3], decreasing=F),,drop=F]
			tht <- tminmaxt[1, ,drop=F]
		}

		if(ca == "BA"){

			tminmaxt <- minmaxt[(ft1[1,1]+1):(ft3[1,1]-1), ,drop=F]
			tminmaxt <- tminmaxt[order(tminmaxt[,3], decreasing=F),,drop=F]
			tht <- tminmaxt[1, ,drop=F]
		}

		if(ca == "BB231"){

			tminmaxt <- minmaxt[(ft1[1,1]+1):(ft4[1,1]-1), ,drop=F]
			tminmaxt <- tminmaxt[order(tminmaxt[,3], decreasing=F),,drop=F]
			tht <- tminmaxt[1, ,drop=F]
```

```
}

if(ca == "BD21"){

		if(ft3[1,2] > 0){
				tminmaxt <- minmaxt[(ft2[1,1]+1):(ft4[1,1]-1), ,drop=F]
				tminmaxt <- tminmaxt[order(tminmaxt[,3], decreasing=F),,drop=F]
				tht <- tminmaxt[1, ,drop=F]
		} else		if(ft3[1,2] < 0){
				tminmaxt <- minmaxt[(ft2[1,1]+1):(ft3[1,1]-1), ,drop=F]
				tminmaxt <- tminmaxt[order(tminmaxt[,3], decreasing=F),,drop=F]
				tht <- tminmaxt[1, ,drop=F]
		}
}

if(ca == "BD21_43"){

		tminmaxt <- minmaxt[(ft2[1,1]+1):(ft3[1,1]-1), ,drop=F]
		tminmaxt <- tminmaxt[order(tminmaxt[,3], decreasing=F),,drop=F]
		tht <- tminmaxt[1, ,drop=F]
}

if(ca == "BA34"){

		tminmaxt <- minmaxt[(ft2[1,1]+1):(ft3[1,1]-1), ,drop=F]
		tminmaxt <- tminmaxt[order(tminmaxt[,3], decreasing=F),,drop=F]
		tht <- tminmaxt[1, ,drop=F]
}

if(ca == "BC"){

		tminmaxt <- minmaxt[(ft2[1,1]+1):(ft1[1,1]-1), ,drop=F]
		tminmaxtt <- tminmaxt[order(tminmaxt[,3], decreasing=F),,drop=F]
		tminmaxty <- tminmaxt[tminmaxt[,4] == 0, ,drop=F]
		tminmaxtyt <- tminmaxty[order(tminmaxty[,2], decreasing=T),,drop=F]
		if(tminmaxt[nrow(tminmaxt),1] - tminmaxtt[1,1] == 0){
				tht <- tminmaxtt[1, ,drop=F]
		} else {
				tht <- tminmaxtyt[1, ,drop=F]
		}
}

if(ca == "BC23"){

		tminmaxt <- minmaxt[(ft3[1,1]+1):(ft1[1,1]-1), ,drop=F]
		tminmaxt <- tminmaxt[order(tminmaxt[,3], decreasing=F),,drop=F]
		tht <- tminmaxt[1, ,drop=F]
}

if(ca == "CA321"){

		tminmaxt <- minmaxt[(ft1[1,1]+1):(ft4[1,1]-1), ,drop=F]
		if(nrow(tminmaxt) >= 3){
				tminmaxt <- tminmaxt[order(tminmaxt[,3], decreasing=F),,drop=F]
						if(tminmaxt[2,3]/tminmaxt[1,3] < 1.5 && tminmaxt[2,3] > tminmaxt[1,3]){
								tht <- tminmaxt[2, ,drop=F]
						} else {
								tht <- tminmaxt[1, ,drop=F]
						}
		} else {
				tht <- tminmaxt[1, ,drop=F]
		}

}

if(ca == "CD"){

		tminmaxt <- minmaxt[(ft2[1,1]+1):(ft1[1,1]-1), ,drop=F]
		tminmaxt <- tminmaxt[order(tminmaxt[,3], decreasing=F),,drop=F]
		tht <- tminmaxt[1, ,drop=F]
}

if(ca == "CF"){

		tminmaxt <- minmaxt[(ft1[1,1]+1):(ft2[1,1]-1), ,drop=F]
		tminmaxt <- tminmaxt[order(tminmaxt[,3], decreasing=F),,drop=F]
		tht <- tminmaxt[1, ,drop=F]
}

if(ca == "CF34" && ft4[1,2] < -100){

		tminmaxt <- minmaxt[(ft1[1,1]+1):(ft2[1,1]-1), ,drop=F]
		tminmaxt <- tminmaxt[order(tminmaxt[,3], decreasing=F),,drop=F]
		tht <- tminmaxt[1, ,drop=F]
}

if(ca == "DA" ){
```

```
				if(ft2[1,3]/ft3[1,3] > 4){
					tminmaxt <- minmaxt[(ft1[1,1]+1):(ft2[1,1]-1), ,drop=F]
					tminmaxt <- tminmaxt[order(tminmaxt[,3], decreasing=F),,drop=F]
					tht <- tminmaxt[1, ,drop=F]
				} else {
					tminmaxt <- minmaxt[(ft1[1,1]+1):(ft3[1,1]-1), ,drop=F]
					tminmaxt <- tminmaxt[order(tminmaxt[,3], decreasing=F),,drop=F]
					tht <- tminmaxt[1, ,drop=F]
				}
			}

			if(ca == "DA12" ){

				tminmaxt <- minmaxt[(ft1[1,1]+1):(ft3[1,1]-1), ,drop=F]
				tminmaxt <- tminmaxt[order(tminmaxt[,3], decreasing=F),,drop=F]
				tht <- tminmaxt[1, ,drop=F]
			}

			if(ca == "DA23"){

				tminmaxt <- minmaxt[(ft1[1,1]+1):(ft2[1,1]-1), ,drop=F]
				tminmaxt <- tminmaxt[order(tminmaxt[,3], decreasing=F),,drop=F]
				tht <- tminmaxt[1, ,drop=F]
			}

			if(ca == "DB"){

				tminmaxt <- minmaxt[(ft1[1,1]+1):(ft2[1,1]-1), ,drop=F]
				tminmaxt <- tminmaxt[tminmaxt[,4] == 0, ,drop=F]
				tminmaxt <- tminmaxt[order(tminmaxt[,1], decreasing=T),,drop=F]
				lt <- nrow(tminmaxt)
				if(lt == 1){
					tht <- tminmaxt[1, ,drop=F]
				} else if(lt == 2){
					if( (tminmaxt[1,3] - tminmaxt[2,3]) /   (tminmaxt[1,2] - tminmaxt[2,2]) < 0 ){
						if(tminmaxt[1,3] < tminmaxt[2,3] ){
							tht <- tminmaxt[1, ,drop=F]
						} else {
							tht <- tminmaxt[2, ,drop=F]

						}
					} else if( minmaxt[tminmaxt[2,1] + 1, 3] / tminmaxt[2,3] > 1.6){
						if(tminmaxt[1,3] < tminmaxt[2,3] ){
							tht <- tminmaxt[1, ,drop=F]
						} else {
							tht <- tminmaxt[2, ,drop=F]

						}
					} else if( (minmaxt[tminmaxt[2,1] + 1, 3] - tminmaxt[2,3]) / ( minmaxt[tminmaxt[2,1] + 1, 2] - tminmaxt[2,2]) <
0.00004 && (tminmaxt[1,3] - minmaxt[tminmaxt[2,1] + 1, 3]) / (tminmaxt[1,2] - minmaxt[tminmaxt[2,1] + 1, 2]) > -0.00002 &&  (tminmaxt[1,3] -
minmaxt[tminmaxt[2,1] + 1, 3]) / (tminmaxt[1,2] - minmaxt[tminmaxt[2,1] + 1, 2]) < -0.00001){
						if(tminmaxt[1,3] < tminmaxt[2,3] ){
							tht <- tminmaxt[1, ,drop=F]
						} else {
							tht <- tminmaxt[2, ,drop=F]

						}
					} else if( minmaxt[tminmaxt[2,1] + 1, 3] / tminmaxt[2,3] > 1.2 && minmaxt[tminmaxt[2,1] + 1, 3] / tminmaxt[2,3]
< 1.3){
						if(tminmaxt[1,3] < tminmaxt[2,3] ){
							tht <- tminmaxt[1, ,drop=F]
						} else {
							tht <- tminmaxt[2, ,drop=F]

						}
					} else if( (tminmaxt[1,3] - tminmaxt[2,3]) /   (tminmaxt[1,2] - tminmaxt[2,2]) > 0 ){
						if(tminmaxt[1,3] < tminmaxt[2,3] ){
							tht <- tminmaxt[2, ,drop=F]
						} else {
							tht <- tminmaxt[1, ,drop=F]

						}
					} else {
						if(tminmaxt[1,3] < tminmaxt[2,3] ){
							tht <- tminmaxt[2, ,drop=F]
						} else {
							tht <- tminmaxt[1, ,drop=F]

						}
					}
				} else if(lt >= 3){
					sl <- cbind(1:lt)
					wa <-c()
					for( s in 1:lt){
						if(s == 1){
							sp <- 0
```

```
                        } else {
                            sp <- (tminmaxt[s - 1,3] - tminmaxt[s,3])/(tminmaxt[s - 1,2] - tminmaxt[s,2])
                        }
                        wa <- rbind(wa,sp)
                    }
                    sl <- cbind(sl,wa)
                    sl <- sl[order(sl[,2], decreasing=T), ,drop=F]
                    if(sl[1,2] == 0){
                        tht <- tminmaxt[sl[2,1] , ,drop=F]
                    } else {
                        tht <- tminmaxt[sl[1,1] , ,drop=F]
                    }
            }
    }

    if(ca == "DB32" ){

            tminmaxt <- minmaxt[(ft1[1,1]+1):(ft2[1,1]-1), ,drop=F]
            tminmaxt <- tminmaxt[tminmaxt[,4] == 0, ,drop=F]
            tminmaxt <- tminmaxt[order(tminmaxt[,1], decreasing=T),,drop=F]
            lt <- nrow(tminmaxt)
            if(lt == 1){
                    tht <- tminmaxt[1, ,drop=F]
            } else if(lt == 2){
                    if((ft2[1,3] - minmaxt[ft2[1,1]-1, 3])/(ft2[1,2] - minmaxt[ft2[1,1]-1, 2]) < 0.0006 || (ft2[1,3] - minmaxt[ft2[1,1]-1,
3])/(ft2[1,2] - minmaxt[ft2[1,1]-1, 2]) > 0.0009){
                        tht <- tminmaxt[2, ,drop=F]
                    } else {
                        tht <- tminmaxt[1, ,drop=F]
                    }
            } else if(lt >= 3){
                    sl <- cbind(1:lt)
                    wa <-c()
                    for( s in 1:lt){
                        if(s == 1){
                            sp <- 0
                        } else {
                            sp <- (tminmaxt[s - 1,3] - tminmaxt[s,3])/(tminmaxt[s - 1,2] - tminmaxt[s,2])
                        }
                        wa <- rbind(wa,sp)
                    }
                    sl <- cbind(sl,wa)
                    sl <- sl[order(sl[,2], decreasing=T), ,drop=F]
                    if(sl[1,2] == 0){
                        tht <- tminmaxt[sl[2,1] , ,drop=F]
                    } else {
                        tht <- tminmaxt[sl[1,1] , ,drop=F]
                    }
            }
    }

    if(ca == "DC21"){

            tminmaxt <- minmaxt[(ft1[1,1]+1):(ft3[1,1]-1), ,drop=F]
            tminmaxt <- tminmaxt[order(tminmaxt[,3], decreasing=F),,drop=F]
            tht <- tminmaxt[1, ,drop=F]
    }

    if(ca == "DC"){

            if(ft4[1,2] < -100 && ft2[1,2] < -50){
                    tminmaxt <- minmaxt[(ft1[1,1]+1):(ft3[1,1]-1), ,drop=F]
                    tminmaxt <- tminmaxt[order(tminmaxt[,3], decreasing=F),,drop=F]
                    tht <- tminmaxt[1, ,drop=F]
            }    else if(ft4[1,2] < -100){
                    tminmaxt <- minmaxt[(ft2[1,1]+1):(ft1[1,1]-1), ,drop=F]
                    tminmaxt <- tminmaxt[order(tminmaxt[,3], decreasing=F),,drop=F]
                    tht <- tminmaxt[1, ,drop=F]
            }
    }

    if(ca == "DD23"){

            tminmaxt <- minmaxt[(ft2[1,1]+1):(ft1[1,1]-1), ,drop=F]
            tminmaxt <- tminmaxt[order(tminmaxt[,3], decreasing=F),,drop=F]
            tht <- tminmaxt[1, ,drop=F]
    }

    if(ca == "DD31"){

            tminmaxt <- minmaxt[(ft2[1,1]+1):(ft1[1,1]-1), ,drop=F]
            tminmaxt <- tminmaxt[order(tminmaxt[,3], decreasing=F),,drop=F]
            tht <- tminmaxt[1, ,drop=F]
    }

    if(ca == "DD"){
```

```
			tminmaxt <- minmaxt[(ft2[1,1]+1):(ft1[1,1]-1), ,drop=F]
			tminmaxt <- tminmaxt[tminmaxt[,4] == 0, ,drop=F]
			tminmaxt <- tminmaxt[order(tminmaxt[,1], decreasing=T),,drop=F]
			lt <- nrow(tminmaxt)
			if(lt == 1){
				tht <- tminmaxt[1, ,drop=F]
			} else if(lt == 2){
				if((ft2[1,3] - minmaxt[ft2[1,1]+1, 3])/(ft2[1,2] - minmaxt[ft2[1,1]+1, 2]) < 0.0007 || (ft2[1,3] - minmaxt[ft2[1,1]+1, 
3])/(ft2[1,2] - minmaxt[ft2[1,1]+1, 2]) > 0.0009){
					if((ft2[1,3] - minmaxt[ft2[1,1]+1, 3])/(ft2[1,2] - minmaxt[ft2[1,1]+1, 2]) < 0){
						tht <- tminmaxt[1, ,drop=F]
					} else {
						tht <- tminmaxt[2, ,drop=F]
					}
				} else {
					tht <- tminmaxt[1, ,drop=F]
				}
			} else if(lt >= 3){
				sl <- cbind(1:lt)
				wa <-c()
				for( s in 1:lt){
					if(s == 1){
						sp <- 0
					} else {
						sp <- (tminmaxt[s - 1,3] - tminmaxt[s,3])/(tminmaxt[s - 1,2] - tminmaxt[s,2])
					}
					wa <- rbind(wa,sp)
				}
				sl <- cbind(sl,wa)
				sl <- sl[order(sl[,2], decreasing=T), ,drop=F]
				if(sl[1,2] == 0){
					tht <- tminmaxt[sl[2,1] , ,drop=F]
				} else {
					tht <- tminmaxt[sl[1,1] , ,drop=F]
				}
			}
		}

		if(ca == "DE"){

			tminmaxt <- minmaxt[(ft1[1,1]+1):(ft2[1,1]-1), ,drop=F]
			tminmaxt <- tminmaxt[order(tminmaxt[,3], decreasing=F),,drop=F]
			tht <- tminmaxt[1, ,drop=F]
		}

		if(ca == "DE31"){

			tminmaxt <- minmaxt[(ft1[1,1]+1):(ft2[1,1]-1), ,drop=F]
			tminmaxt <- tminmaxt[order(tminmaxt[,3], decreasing=F),,drop=F]
			tht <- tminmaxt[1, ,drop=F]
		}

		if(ca == "DE43"){

			tminmaxt <- minmaxt[(ft1[1,1]+1):(ft2[1,1]-1), ,drop=F]
			tminmaxt <- tminmaxt[order(tminmaxt[,3], decreasing=F),,drop=F]
			tht <- tminmaxt[1, ,drop=F]
		}

		if(ca == "DE431"){

			tminmaxt <- minmaxt[(ft1[1,1]+1):(ft2[1,1]-1), ,drop=F]
			tminmaxt <- tminmaxt[order(tminmaxt[,3], decreasing=F),,drop=F]
			tht <- tminmaxt[1, ,drop=F]
		}

		if(ca == "DF432" ){

			tminmaxt <- minmaxt[(ft2[1,1]+1):(ft1[1,1]-1), ,drop=F]
			tminmaxt <- tminmaxt[order(tminmaxt[,3], decreasing=F),,drop=F]
			tht <- tminmaxt[1, ,drop=F]
		}

		if(ca == "DF32" ){

			tminmaxt <- minmaxt[(ft2[1,1]+1):(ft1[1,1]-1), ,drop=F]
			tminmaxt <- tminmaxt[order(tminmaxt[,3], decreasing=F),,drop=F]
			tht <- tminmaxt[1, ,drop=F]
		}

		if(ca == "DF43" ){

			tminmaxt <- minmaxt[(ft2[1,1]+1):(ft1[1,1]-1), ,drop=F]
			tminmaxt <- tminmaxt[order(tminmaxt[,3], decreasing=F),,drop=F]
			tht <- tminmaxt[1, ,drop=F]
		}
```

```
if(ca == "DF21" ){

            tminmaxt <- minmaxt[(ft3[1,1]+1):(ft1[1,1]-1), ,drop=F]
            tminmaxt <- tminmaxt[order(tminmaxt[,3], decreasing=F),,drop=F]
            tht <- tminmaxt[1, ,drop=F]
}

if(ca == "DF" ){

            tminmaxt <- minmaxt[(ft2[1,1]+1):(ft1[1,1]-1), ,drop=F]
            tminmaxt <- tminmaxt[order(tminmaxt[,3], decreasing=F),,drop=F]
            tht <- tminmaxt[1, ,drop=F]
}

if(ca == "AA123" || ca == "AA1234" ){
            tminmaxt <- minmaxt[(ft1[1,1]+1):(ft4[1,1]-1), ,drop=F]
            tminmaxt <- tminmaxt[order(tminmaxt[,3], decreasing=F),,drop=F]
            tht <- tminmaxt[1, ,drop=F]
}

if(ca == "AB124" || ca == "AB1243" ){
            tminmaxt <- minmaxt[(ft1[1,1]+1):(ft3[1,1]-1), ,drop=F]
            tminmaxt <- tminmaxt[order(tminmaxt[,3], decreasing=F),,drop=F]
            tht <- tminmaxt[1, ,drop=F]
}

if(ca =="AC13" || ca == "AC13_24" || ca =="AC132" || ca =="AC1324"){
            tminmaxt <- minmaxt[(ft1[1,1]+1):(ft2[1,1]-1), ,drop=F]
            tminmaxt <- tminmaxt[order(tminmaxt[,3], decreasing=F),,drop=F]
            tht <- tminmaxt[1, ,drop=F]
}

if(ca == "AD13" || ca == "AD13_42" || ca == "AD134" || ca == "AD1342" || ca == "AD42" ){
            tminmaxt <- minmaxt[(ft1[1,1]+1):(ft2[1,1]-1), ,drop=F]
            tminmaxt <- tminmaxt[order(tminmaxt[,3], decreasing=F),,drop=F]
            tht <- tminmaxt[1, ,drop=F]
}

if(ca == "AE1342" || ca == "AE14" || ca == "AE14_23" || ca == "AE142" ){
            tminmaxt <- minmaxt[(ft1[1,1]+1):(ft2[1,1]-1), ,drop=F]
            tminmaxt <- tminmaxt[order(tminmaxt[,3], decreasing=F),,drop=F]
            tht <- tminmaxt[1, ,drop=F]
}

if(ca == "AF14" || ca == "AF14_32" || ca == "AF143" || ca == "AF1432" ){
            tminmaxt <- minmaxt[(ft1[1,1]+1):(ft2[1,1]-1), ,drop=F]
            tminmaxt <- tminmaxt[order(tminmaxt[,3], decreasing=F),,drop=F]
            tht <- tminmaxt[1, ,drop=F]
}

if(ca == "BA13" || ca == "BA134" || ca == "BA2134" ){
            tminmaxt <- minmaxt[(ft1[1,1]+1):(ft4[1,1]-1), ,drop=F]
            tminmaxt <- tminmaxt[order(tminmaxt[,3], decreasing=F),,drop=F]
            tht <- tminmaxt[1, ,drop=F]
}

if(ca == "BB" || ca == "BB14" || ca == "BB23" || ca == "BB23_14" || ca == "BB2314" || ca == "BB31" || ca == "BB314" ){
            tminmaxt <- minmaxt[(ft1[1,1]+1):(ft4[1,1]-1), ,drop=F]
            tminmaxt <- tminmaxt[order(tminmaxt[,3], decreasing=F),,drop=F]
            tht <- tminmaxt[1, ,drop=F]
}

if(ca == "BC23_41" || ca == "BC234" || ca == "BC2341" || ca == "BC34" || ca == "BC341" || ca == "BC41" ){
            tminmaxt <- minmaxt[(ft2[1,1]+1):(ft1[1,1]-1), ,drop=F]
            tminmaxt <- tminmaxt[order(tminmaxt[,3], decreasing=F),,drop=F]
            tht <- tminmaxt[1, ,drop=F]
}

if(ca == "BD" || ca == "BD14" || ca == "BD143" || ca == "BD214" || ca == "BD2143" || ca == "BD43" ){
            tminmaxt <- minmaxt[(ft2[1,1]+1):(ft3[1,1]-1), ,drop=F]
            tminmaxt <- tminmaxt[order(tminmaxt[,3], decreasing=F),,drop=F]
            tht <- tminmaxt[1, ,drop=F]
}

if(ca == "BE" || ca == "BE13" || ca == "BE24" || ca == "BE24_13" || ca == "BE241" || ca == "BE2413" || ca == "BE41" || ca == "BE413" ){
            tminmaxt <- minmaxt[(ft2[1,1]+1):(ft1[1,1]-1), ,drop=F]
            tminmaxt <- tminmaxt[order(tminmaxt[,3], decreasing=F),,drop=F]
            tht <- tminmaxt[1, ,drop=F]
}

if(ca == "BF" || ca == "BF24" || ca == "BF24_31" || ca == "BF243" || ca == "BF2431" || ca == "BF31" || ca == "BF43" || ca == "BF431" ){
            tminmaxt <- minmaxt[(ft2[1,1]+1):(ft1[1,1]-1), ,drop=F]
            tminmaxt <- tminmaxt[order(tminmaxt[,3], decreasing=F),,drop=F]
            tht <- tminmaxt[1, ,drop=F]
}

if(ca == "CA" || ca == "CA14" || ca == "CA21" || ca == "CA214" || ca == "CA32" || ca == "CA32_14" || ca == "CA3214" ){
```

```
			tminmaxt <- minmaxt[(ft2[1,1]+1):(ft4[1,1]-1), ,drop=F]
			tminmaxt <- tminmaxt[order(tminmaxt[,3], decreasing=F),,drop=F]
			tht <- tminmaxt[1, ,drop=F]
		}

		if(ca == "CB" || ca == "CB12" || ca == "CB124" || ca == "CB24" || ca == "CB31" || ca == "CB31_24" || ca == "CB312" || ca == "CB3124" ){
			tminmaxt <- minmaxt[(ft1[1,1]+1):(ft2[1,1]-1), ,drop=F]
			tminmaxt <- tminmaxt[order(tminmaxt[,3], decreasing=F),,drop=F]
			tht <- tminmaxt[1, ,drop=F]
		}

		if(ca == "CC" || ca == "CC14" || ca == "CC142" || ca == "CC31" || ca == "CC31_42" || ca == "CC314" || ca == "CC3142" || ca == "CC42" ){
			tminmaxt <- minmaxt[(ft1[1,1]+1):(ft2[1,1]-1), ,drop=F]
			tminmaxt <- tminmaxt[order(tminmaxt[,3], decreasing=F),,drop=F]
			tht <- tminmaxt[1, ,drop=F]
		}

		if(ca == "CD24" || ca == "CD241" || ca == "CD3142" || ca == "CD32" || ca == "CD32_41" || ca == "CD324" || ca == "CD41" ){
			tminmaxt <- minmaxt[(ft2[1,1]+1):(ft1[1,1]-1), ,drop=F]
			tminmaxt <- tminmaxt[order(tminmaxt[,3], decreasing=F),,drop=F]
			tht <- tminmaxt[1, ,drop=F]
		}

		if(ca == "CE" || ca == "CE21" || ca == "CE34" || ca == "CE34_21" || ca == "CE342" || ca == "CE3421" || ca == "CE42" || ca == "CE421" ){
			tminmaxt <- minmaxt[(ft2[1,1]+1):(ft1[1,1]-1), ,drop=F]
			tminmaxt <- tminmaxt[order(tminmaxt[,3], decreasing=F),,drop=F]
			tht <- tminmaxt[1, ,drop=F]
		}

		if(ca == "CF12" || ca == "CF34_12" || ca == "CF341" || ca == "CF3412" || ca == "CF41" || ca == "CF412" ){
			tminmaxt <- minmaxt[(ft1[1,1]+1):(ft2[1,1]-1), ,drop=F]
			tminmaxt <- tminmaxt[order(tminmaxt[,3], decreasing=F),,drop=F]
			tht <- tminmaxt[1, ,drop=F]
		}

		if(ca == "DA123" || ca == "DA41" || ca == "DA41_23" || ca == "DA412" || ca == "DA4123" ){
			tminmaxt <- minmaxt[(ft1[1,1]+1):(ft3[1,1]-1), ,drop=F]
			tminmaxt <- tminmaxt[order(tminmaxt[,3], decreasing=F),,drop=F]
			tht <- tminmaxt[1, ,drop=F]
		}

		if(ca == "DB13" || ca == "DB132" || ca == "DB41" || ca == "DB41_32" || ca == "DB413" || ca == "DB4132" ){
			tminmaxt <- minmaxt[(ft1[1,1]+1):(ft2[1,1]-1), ,drop=F]
			tminmaxt <- tminmaxt[order(tminmaxt[,3], decreasing=F),,drop=F]
			tht <- tminmaxt[1, ,drop=F]
		}

		if(ca == "DC13" || ca == "DC213" || ca == "DC42" || ca == "DC42_13" || ca == "DC421" || ca == "DC4213" ){
			tminmaxt <- minmaxt[(ft1[1,1]+1):(ft3[1,1]-1), ,drop=F]
			tminmaxt <- tminmaxt[order(tminmaxt[,3], decreasing=F),,drop=F]
			tht <- tminmaxt[1, ,drop=F]
		}

		if(ca == "DD231" || ca == "DD42" || ca == "DD42_31" || ca == "DD423" || ca == "DD4231" ){
			tminmaxt <- minmaxt[(ft2[1,1]+1):(ft1[1,1]-1), ,drop=F]
			tminmaxt <- tminmaxt[order(tminmaxt[,3], decreasing=F),,drop=F]
			tht <- tminmaxt[1, ,drop=F]
		}

		if(ca == "DE12" || ca == "DE312" || ca == "DE43_12" || ca == "DE4312" ){
			tminmaxt <- minmaxt[(ft1[1,1]+1):(ft2[1,1]-1), ,drop=F]
			tminmaxt <- tminmaxt[order(tminmaxt[,3], decreasing=F),,drop=F]
			tht <- tminmaxt[1, ,drop=F]
		}

		if(ca == "DF321" || ca == "DF43_21" || ca == "DF4321" ){
			tminmaxt <- minmaxt[(ft2[1,1]+1):(ft1[1,1]-1), ,drop=F]
			tminmaxt <- tminmaxt[order(tminmaxt[,3], decreasing=F),,drop=F]
			tht <- tminmaxt[1, ,drop=F]
		}


} else if(nrow(temax) == 3){

## ft1, ft2, ft3

		if(ft1[1,1] < ft2[1,1] && ft2[1,1] < ft3[1,1]){
			if(ft1[1,3]/ft2[1,3] < bai && abs(ft1[1,2]-ft2[1,2]) < dis && ft2[1,3]/ft3[1,3] < bai && abs(ft2[1,2]-ft3[1,2]) < dis){
				ca <- "a123"
			} else if(ft2[1,3]/ft3[1,3] < bai && abs(ft2[1,2]-ft3[1,2]) < dis){
				ca <- "a23"
			} else			if(ft1[1,3]/ft2[1,3] < bai && abs(ft1[1,2]-ft2[1,2]) < dis){
				ca <- "a12"
			} else {
				ca <- "a"
			}
		}
```

```
## ft1, ft3, ft2

	if(ft1[1,1] < ft3[1,1] && ft3[1,1] < ft2[1,1]){
		if(ft1[1,3]/ft3[1,3] < bai && abs(ft1[1,2]-ft3[1,2]) < dis && ft3[1,3]/ft2[1,3] < bai && abs(ft3[1,2]-ft2[1,2]) < dis){
			ca <- "b132"
		} else if(ft3[1,3]/ft2[1,3] < bai && abs(ft3[1,2]-ft2[1,2]) < dis){
			ca <- "b32"
		} else				if(ft1[1,3]/ft3[1,3] < bai && abs(ft1[1,2]-ft3[1,2]) < dis){
			ca <- "b13"
		} else {
			ca <- "b"
		}
	}

## ft2, ft1, ft3

	if(ft2[1,1] < ft1[1,1] && ft1[1,1] < ft3[1,1]){
		if(ft2[1,3]/ft1[1,3] < bai && abs(ft2[1,2]-ft1[1,2]) < dis && ft1[1,3]/ft3[1,3] < bai && abs(ft1[1,2]-ft3[1,2]) < dis){
			ca <- "c213"

		} else if(ft1[1,3]/ft3[1,3] < bai && abs(ft1[1,2]-ft3[1,2]) < dis){
			ca <- "c13"
		} else				if(ft2[1,3]/ft1[1,3] < bai && abs(ft2[1,2]-ft1[1,2]) < dis){
			ca <- "c21"
		} else {
			ca <- "c"
		}
	}

## ft2, ft3, ft1

	if(ft2[1,1] < ft3[1,1] && ft3[1,1] < ft1[1,1]){
		if(ft2[1,3]/ft3[1,3] < bai && abs(ft2[1,2]-ft3[1,2]) < dis && ft3[1,3]/ft1[1,3] < bai && abs(ft3[1,2]-ft1[1,2]) < dis){
			ca <- "d231"

		} else if(ft3[1,3]/ft1[1,3] < bai && abs(ft3[1,2]-ft1[1,2]) < dis){
			ca <- "d31"
		} else if(ft2[1,3]/ft3[1,3] < bai && abs(ft2[1,2]-ft3[1,2]) < dis){
			ca <- "d23"
		} else {
			ca <- "d"
		}
	}

## ft3, ft2, ft1

	if(ft3[1,1] < ft2[1,1] && ft2[1,1] < ft1[1,1]){
		if(ft3[1,3]/ft2[1,3] < bai && abs(ft3[1,2]-ft2[1,2]) < dis && ft2[1,3]/ft1[1,3] < bai && abs(ft2[1,2]-ft1[1,2]) < dis){
			ca <- "e321"

		} else if(ft2[1,3]/ft1[1,3] < bai && abs(ft2[1,2]-ft1[1,2]) < dis){
			ca <- "e21"
		} else if(ft3[1,3]/ft2[1,3] < bai && abs(ft3[1,2]-ft2[1,2]) < dis){
			ca <- "e32"
		} else {
			ca <- "e"
		}
	}

## ft3, ft1, ft2

	if(ft3[1,1] < ft1[1,1] && ft1[1,1] < ft2[1,1]){
		if(ft3[1,3]/ft1[1,3] < bai && abs(ft3[1,2]-ft1[1,2]) < dis && ft1[1,3]/ft2[1,3] < bai && abs(ft1[1,2]-ft2[1,2]) < dis){
			ca <- "f312"
		} else if(ft1[1,3]/ft2[1,3] < bai && abs(ft1[1,2]-ft2[1,2]) < dis){
			ca <- "f12"
		} else if(ft3[1,3]/ft1[1,3] < bai && abs(ft3[1,2]-ft1[1,2]) < dis){
			ca <- "f31"
		} else {
			ca <- "f"
		}
	}

#### extract a separation point by patterns


		if(ca == "b"){
			if(ft3[1,2] - ft1[1,2] > 35){
				tminmaxt <- minmaxt[(ft1[1,1]+1):(ft3[1,1]-1), ,drop=F]
				tminmaxt <- tminmaxt[order(tminmaxt[,3], decreasing=F),,drop=F]
				tht <- tminmaxt[1, ,drop=F]
			} else {
				if(minmaxt[ft2[1,1] - 1,3] < minmaxt[ft3[1,1] - 1,3]){
					tht<- minmaxt[ft2[1,1] - 1, ,drop=F]
				} else {
					tht<- minmaxt[ft3[1,1] - 1, ,drop=F]
```

```
			}
		}
	}

	if(ca == "d23"){
		if(ft2[1,2] > -60){
			tht <- minmaxt[ft3[1,1] + 1, ,drop=F]
		} else {
			if(ft1[1,1] != 1 && ft1[1,1] != nrow(minmaxt) ){
				tht <- minmaxt[ft1[1,1] + 1, ,drop=F]
			} else {
				tht <- c()
			}
		}
	}

	if(ca == "f"){
		tht <- minmaxt[ft1[1,1] + 1, ,drop=F]
	}
	if(ca == "e32"){
		if(ft3[1,2] > -40){
			tht <- minmaxt[ft2[1,1] + 1, ,drop=F]
		} else {

			if(ft1[1,1] != 1 && ft1[1,1] != nrow(minmaxt) ){
				tht <- minmaxt[ft1[1,1] + 1, ,drop=F]
			} else {
				tht <- c()
			}
		}
	}
	if(ca == "f12"){
		if(ft3[1,2] >-45){
			tht <- minmaxt[ft3[1,1] + 1, ,drop=F]
		} else {
			if(ft2[1,1] != 1 && ft2[1,1] != nrow(minmaxt) ){
				tht <- minmaxt[ft2[1,1] + 1, ,drop=F]
			} else {
				tht <- c()
			}
		}
	}
	if(ca == "e"){
		if(ft1[1,2] > -60){
			tht <- minmaxt[ft2[1,1] + 1, ,drop=F]
		} else {
			tht <- minmaxt[ft3[1,1] + 1, ,drop=F]
		}
	}
	if(ca == "a"){
		if(ft2[1,2] -ft1[1,2] > 50){
			tminmaxt <- minmaxt[(ft1[1,1]+1):(ft2[1,1]-1), ,drop=F]
			tminmaxt <- tminmaxt[order(tminmaxt[,3], decreasing=F),,drop=F]
			tht <- tminmaxt[1, ,drop=F]
		} else {
			if(ft1[1,2] < -10){
				if(minmaxt[ft1[1,1] + 1, 3] <= minmaxt[ft2[1,1] - 1,3] && minmaxt[ft2[1,1] - 1,3]
<= minmaxt[ft2[1,1] + 1, 3]){

					tht <- minmaxt[ft1[1,1] + 1, ,drop=F]
				} else if(minmaxt[ft2[1,1] - 1,3] <= minmaxt[ft2[1,1] + 1, 3] || ft2[1,2] > -10){
					tht <- minmaxt[ft2[1,1] - 1, ,drop=F]
				} else {
					tht <- minmaxt[ft2[1,1] + 1, ,drop=F]
				}
			} else {
				tht <- minmaxt[ft1[1,1] + 1, ,drop=F]
			}
		}
	}
	if(ca == "c"){
		if(ft3[1,2] < 10){
			tht <- minmaxt[ft1[1,1] + 1, ,drop=F]
		} else {
			tht <- minmaxt[ft1[1,1] - 1, ,drop=F]
		}
	}
	if(ca == "d31"){
		if(ft3[1,2] > -40){
			tht <- minmaxt[ft3[1,1] + 1, ,drop=F]
		} else {
			if(ft1[1,1] != 1 && ft1[1,1] != nrow(minmaxt) ){
				tht <- minmaxt[ft1[1,1] + 1, ,drop=F]
			} else {
				tht <- c()
			}
		}
	}
```

```
			if(ca == "d"){
				if(ft2[1,2] > -40){
					tht <- minmaxt[ft2[1,1] + 1, ,drop=F]
				} else if(ft3[1,2] > -40){
					tht <- minmaxt[ft3[1,1] + 1, ,drop=F]
				} else if(ft1[1,2] > -10) {
					tht <- minmaxt[ft1[1,1] - 1, ,drop=F]
				} else {
					tht <- minmaxt[ft1[1,1] + 1, ,drop=F]
				}
			}
			if(ca == "f31"){
				tht <- minmaxt[ft1[1,1] + 1, ,drop=F]
			}
			if(ca == "a23"){
				if(ft2[1,2] < -45){
					tht <- minmaxt[ft3[1,1] + 1, ,drop=F]
				} else {
					tht <- minmaxt[ft1[1,1] + 1, ,drop=F]
				}
			}
			if(ca == "a12"){
				tht <- minmaxt[ft2[1,1] + 1, ,drop=F]
			}
			if(ca == "e21"){
				tht <- minmaxt[ft3[1,1] + 1, ,drop=F]
			}
			if(ca == "c21"){
				tht <- minmaxt[ft3[1,1] - 1, ,drop=F]
			}
			if(ca == "f312"){
				tht <- minmaxt[ft2[1,1] + 1, ,drop=F]
			}
			if(ca == "b32"){
				if(minmaxt[ft1[1,1] + 1, 3] <= minmaxt[ft3[1,1] - 1,3]){
					tht <- minmaxt[ft1[1,1] + 1, ,drop=F]
				} else if(ft3[1,2] < -10){
					tht <- minmaxt[ft3[1,1] - 1, ,drop=F]
				} else {
					tht <- minmaxt[ft1[1,1] + 1, ,drop=F]
				}
			}
			if(ca == "e32"){
				tht <- minmaxt[ft1[1,1] - 1, ,drop=F]
			}
			if(ca == "c21"){
				tht <- minmaxt[ft1[1,1] + 1, ,drop=F]
			}
			if(ca == "a123"){
				if(ft1[1,2] < -60 && ft4[1,2] > -60){
					tht <- minmaxt[ft4[1,1] + 1, ,drop=F]
				} else {
					tht <- minmaxt[ft3[1,1] + 1, ,drop=F]
				}
			}
			if(ca == "c213"){
				tht <- minmaxt[ft3[1,1] + 1, ,drop=F]
			}
			if(ca == "e321"){
				if(ft1[1,2] > 0){
					tht <- minmaxt[ft1[1,1] - 1, ,drop=F]

				} else {
					tht <- minmaxt[ft1[1,1] + 1, ,drop=F]
				}
			}

			if(ca == "d231"){
				if(ft1[1,2] > -10){
					tht <- minmaxt[ft1[1,1] - 1, ,drop=F]
				} else {
					tht <- minmaxt[ft1[1,1] + 1, ,drop=F]
				}
			}

} else if(nrow(temax) == 2){
		tht <- minmaxt[3, ,drop=F]
		if(minmaxt[2,3] < 1.0e-4){
			tht <- c()
		}
} else {
		tht <- c()
}


th <- tht[1,2]
```

```
text(0,0, label=th)

an <- 1/0.9^(th)

### The value below the threshold is replaced with 0.
### In the example, file names a1_a.txt and a2_a.txt are added with "_a" after the input file name.
### The data is described in the file “a1_a.txt” in the following format.
### 0.713274
### 0.170416
### 0
### 0
### 0
### 0.379159
### 0.436011
### 0.346797
### <snip>
###
####

            xyz <- y$va0
            xyz[xyz<an]<-0

            smo <- paste(listlist$V1[mm],"_a.txt", sep="")

            write(xyz, file=smo, ncolumns=1)

            cxyz <- table(xyz>0)

### Output file name "2018cent.txt"
### Active bins and inactive bins separated by the threshold,
### the active bins, the inactive bins, the threshold, and the input file name are described in the output file "2018cent.txt".
### For example
### filename threshold active bins inactive bins
### a1.txt 0.119149149720695 24853 306018
### a2.txt 0.095528323822769 62935 206791

kotae <- c( fil,ca, an, cxyz)
write(kotae, file="2018cent.txt", append=T, ncolumns =5)

             }

dev.off()
```
